# Supplementary material for: A common NFKB1 variant detected through antibody analysis in UK Biobank predicts risk of infection and allergy
Source: Am J Hum Genet. 2024 Jan 16;111(2):295–308. doi: 10.1016/j.ajhg.2023.12.013 (PMC10870136; doi:10.1016/j.ajhg.2023.12.013)
Supplement: Document S1. Figures S1–S4 and Tables S1–S3 and S5–S17 [file mmc1.pdf]

The American Journal of Human Genetics, Volume *111*

Supplemental information

A common *NFKB1* variant detected through antibody analysis in UK Biobank predicts risk of infection and allergy

Amanda Y. Chong, Nicole Brenner, Andres Jimenez-Kaufmann, Adrian Cortes, Michael Hill, Thomas J. Littlejohns, James J. Gilchrist, Benjamin P. Fairfax, Julian C. Knight, Flavia Hodel, Jacques Fellay, Gil McVean, Andres Moreno-Estrada, Tim Waterboer, Adrian V.S. Hill, and Alexander J. Mentzer

Supplemental Figures

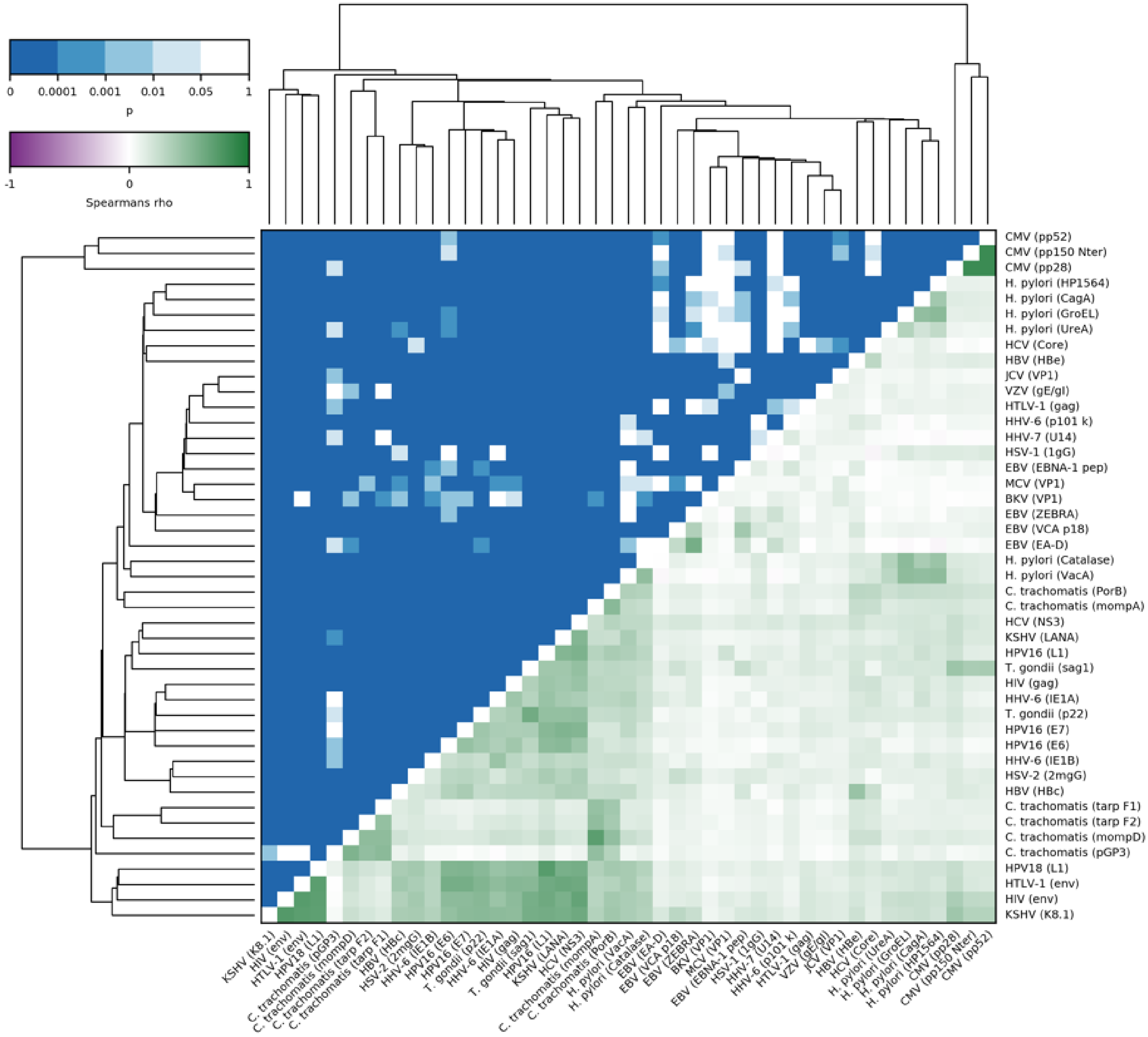

Figure S1: All antibody responses were generally positively correlated. Antibody responses to different antigens from the same pathogen tended to cluster together and were more strongly correlated with each other. No evidence of unexpected correlation between antibody traits was detected. Pairwise correlations between antibody responses are shown in the bottom triangle, p-values are indicated in the upper triangle

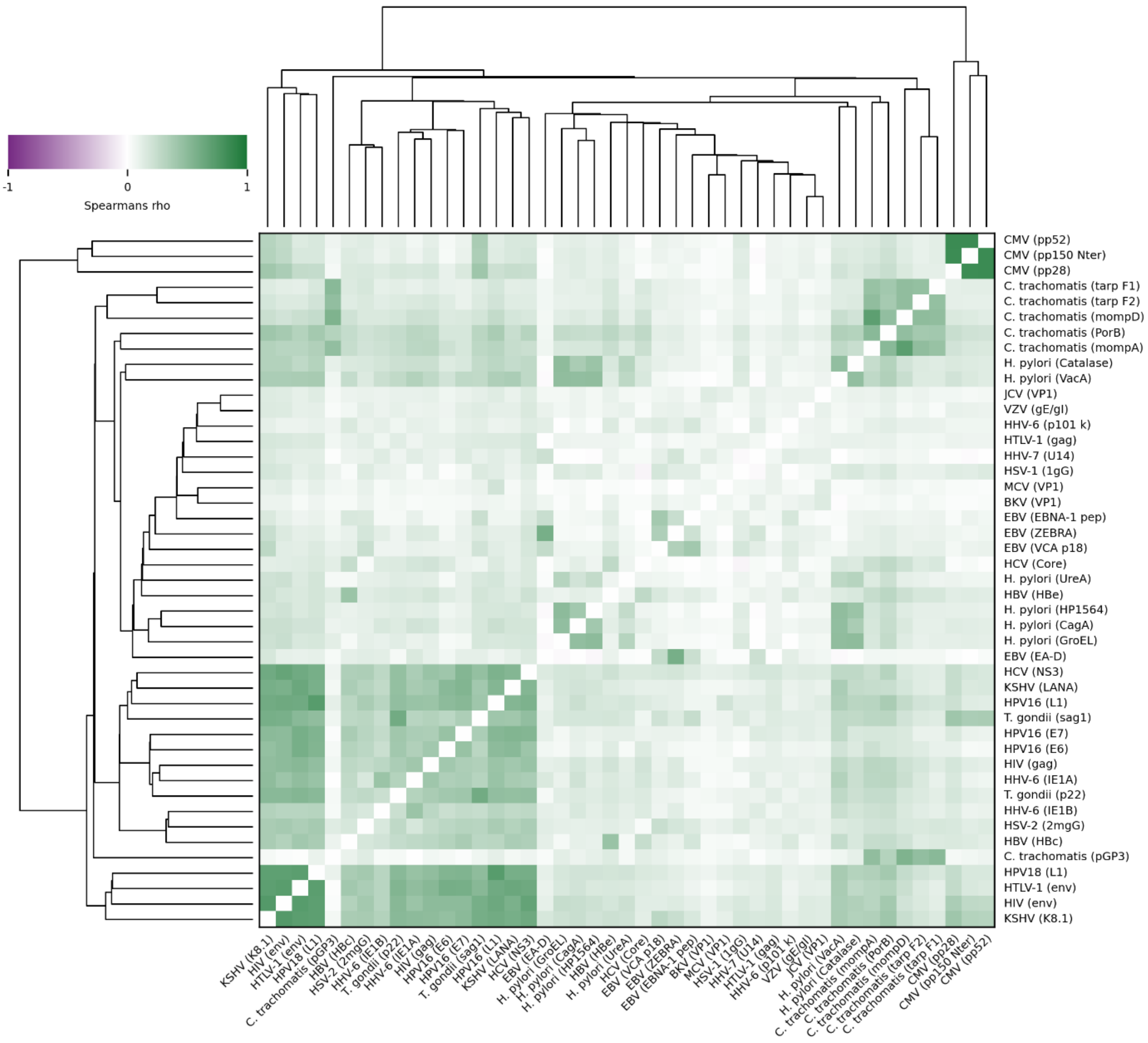

Figure S2: Antibody responses remained correlated after accounting for effects of age, sex, and lead GWAS SNPs. Pairwise correlations between antibody responses accounting for age and sex are shown in the bottom triangle, correlations accounting for age, sex, and genetic variants are indicated in the upper triangle.

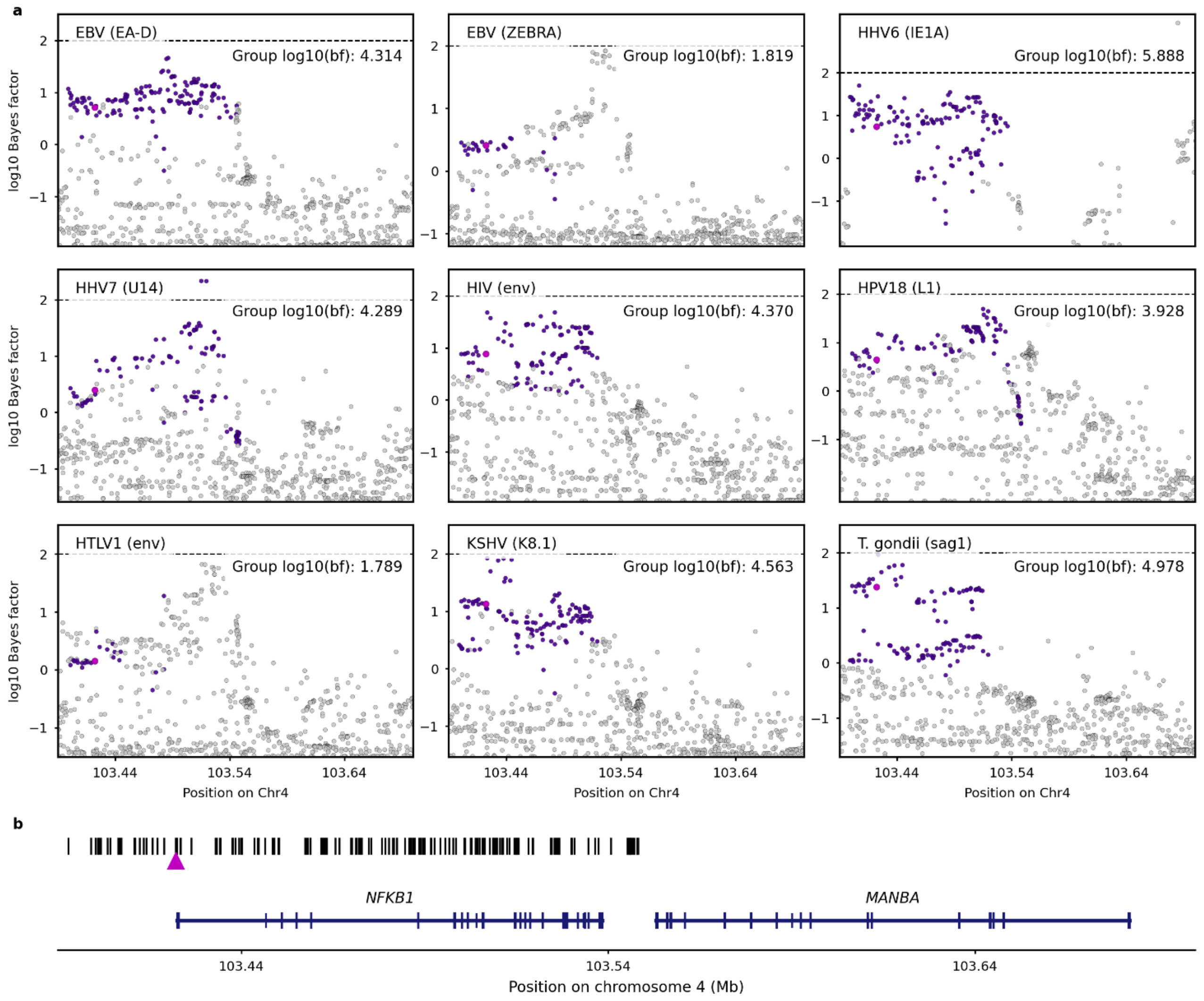

Figure S3: Associations on Chromosome 4 fine-mapped to the *NFKB1* locus. (a) Fine-mapping results for significantly associated antibody responses. Log10 Bayes factors for individual variants are shown on the y axis. Predicted causal groups of variants containing rs28362491 for each phenotype are in purple, rs28362491 is highlighted in pink. Log10 Bayes factors for the entire causal group are also shown. (b) Location of all predicted causal variants from (a) relative to *NFKB1* and *MANBA* on chromosome 4. Location of rs28362491 is indicated by a pink triangle.

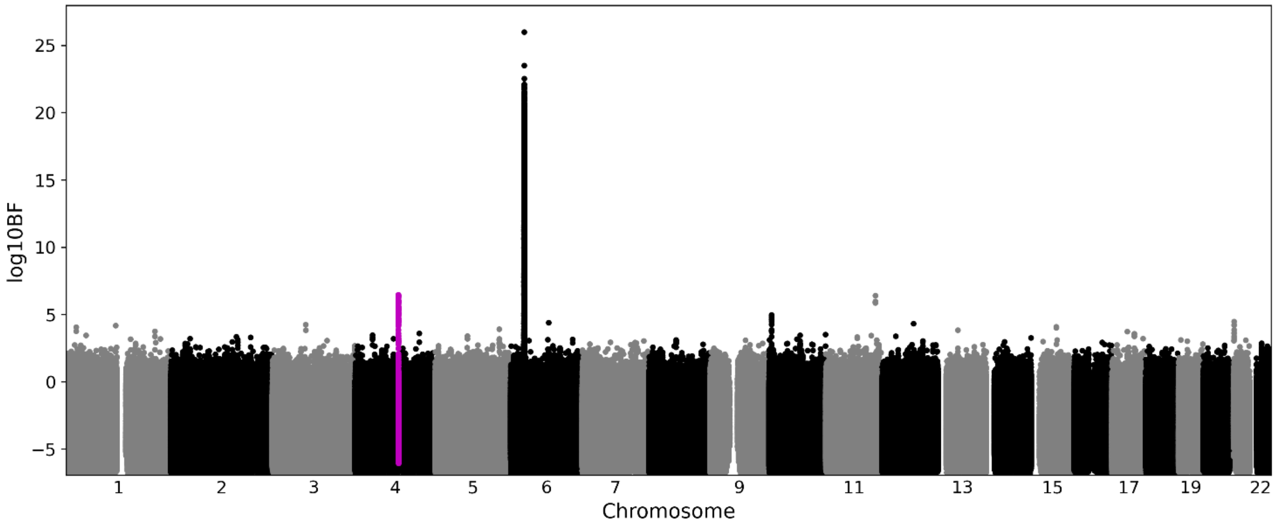

Figure S4: Bayesian-based multivariate GWAS including seven significant phenotypes supports a shared association at *NFKB1*. Included phenotypes: EBV (EAD), HHV-6 (IE1A), HHV-7 (U14), HIV (env), HPV18 (L1), KSHV (K8.1), and *T. gondii* (sag1). The *NFKB1* region is highlighted in pink.

Supplemental Tables

Table S1: Details of the 45 antigens and 20 pathogens covered in the UK Biobank serology panel

Infectious agent	Antigen	Likely Function	Reference
HSV-1	1gG	membrane glycoprotein	1
HSV-2	2mgGunique	membrane glycoprotein	1
VZV	gE/gI	envelope glycoproteins	1
EBV	VCAp18	capsid protein	1
	EBNA1	replication, latent viral infection	
	ZEBRA	replication activator	
	EA-D	replication (polymerase accessory subunit)	
CMV	pp150 (N-terminus)	tegument protein	1
	pp52	DNA binding protein	
	pp28	capsid protein	
HHV-6	IE1B	potential transactivator	Validation ongoing
	IE1A	potential transactivator	
	p101k	potential tegument protein	
HHV-7	U14	potential tegument protein	Validation ongoing
KSHV	LANA	replication and long-term persistence	Validation ongoing
	K8.1	structural glycoprotein	
HBV	HBc	core antigen	2
	HBe	soluble nucleocapsid associated antigen	
HCV	Core	structural antigen	2; 3
	NS3	protease and RNA helicase activity	
Toxoplasma gondii	p22	surface protein	2
	sag1	surface protein	
HTLV-1	Gag	structural antigen	2
	Env	structural antigen	
HIV-1	Gag	structural antigen	4
	Env	structural antigen	
BKV	VP1	major capsid protein	5-7
JCV	VP1	major capsid protein	5-7
MCV	VP1	major capsid protein	5; 7
HPV16	L1	major capsid protein	8; 9
	E6	oncogene	
	E7	oncogene	
HPV18	L1	major capsid protein	8
Chlamydia trachomatis	mompD	structural antigen	10; 11
	mompA	structural antigen	
	TarpDF1	regulation of actin recruitment	
	TarpDF2		
	PorB	porin	
	pGP3	virulence factor	
Helicobacter pylori	CagA (N-terminus)	pathogenesis	12
	VacA (C-terminus)	pathogenesis	
	HP1564	cell envelope	
	GroEL	chaperonin	
	Catalase	detoxification	
	UreA	urease alpha subunit	
-	GST	used for background subtraction	9

Table S2: Distributions of serum antibody responses in UK Biobank prior to normalisation

Antigen	No. samples	mean	SD	median	max	min
HSV-1 (1gG)	9695	3192.093	3004.911	2974	15718	1
HSV-2 (2mgG)	9695	272.784	868.506	32	10630	1
VZV (gE/gI)	9695	978.344	1195.509	520	11635	1
EBV (VCA p18)	9695	7289.483	3419.997	7473	19010	1
EBV (EBNA-1 pep)	9695	4425.043	3116.959	4324	16969	1
EBV (ZEBRA)	9695	2348.944	1965.866	1990	13637	1
EBV (EA-D)	9695	2558.274	2588.912	1735	15853	1
CMV (pp150 Nter)	9695	1602.94	2014.004	597	13233	1
CMV (pp52)	9695	3177.482	3411.886	2010	14979	1
CMV (pp28)	9695	1343.486	1657.125	491	11120	1
HHV-7 (U14)	9695	848.617	804.376	600	10321	1
KSHV (LANA)	9695	92.937	590.933	16	12897	1
KSHV (K8.1)	9695	73.157	122.948	57	4375	1
HHV-6 (IE1B)	9695	521.343	885.102	233	12488	1
HHV-6 (IE1A)	9695	314.626	375.556	201	5678	1
HHV-6 (p101 k)	9695	149.713	463.484	21	11715	1
HBV (HBc)	9695	71.24	610.159	5	14302	1
HBV (HBe)	9695	97.549	578.451	13	13906	1
HCV (Core)	9695	31.179	314.593	3	12542	1
HCV (NS3)	9695	53.024	347.801	31	13384	1
T. gondii (p22)	9695	74.733	194.781	31	7153	1
T. gondii (sag1)	9695	128.452	153.086	90	5787.8	1
HTLV-1 (gag)	9695	292.457	356.300	134	2717	1
HTLV-1 (env)	9695	33.871	41.442	27	2203	1
HIV (gag)	9695	126.77	362.583	55	10819	1
HIV (env)	9695	47.215	70.339	39	3972	1
BKV (VP1)	9695	3763.077	2706.281	3428	14978	1
JCV (VP1)	9695	792.817	1163.624	314	10975	1
MCV (VP1)	9695	2214.352	2449.434	1006	10506	1
HPV16 (L1)	9695	67.737	171.04	40	4983	1
HPV16 (E6)	9695	35.296	296.766	11	11781	1
HPV16 (E7)	9695	40.78	188.208	18	9305	1
HPV18 (L1)	9695	56.813	143.47	38	5876	1
C. trachomatis (mompD)	9695	138.612	478.048	16	8022	1
C. trachomatis (mompA)	9695	80.089	300.058	19	7870	1
C. trachomatis (tarp F2)	9695	201.311	578.011	25	9199	1
C. trachomatis (tarp F1)	9695	187.744	636.115	12	10384	1
C. trachomatis (PorB)	9695	23.29	57.587	13	2599	1
C. trachomatis (pGP3)	9695	607.845	1615.422	9	13749	1
H. pylori (CagA)	4871	994.417	2277.809	72	14106	1
H. pylori (VacA)	9695	178.468	651.847	28	10661	1
H. pylori (HP1564)	9695	500.069	1176.451	43	12055	1
H. pylori (GroEL)	9695	1021.471	2224.667	21	13150	1

H. pylori (Catalase)	9695	589.451	1874.24	41	13972	1
H. pylori (UreA)	9695	434.729	1422.169	34	14085	1

Table S3: Cohort characteristics for UK Biobank and CoLaus/PsyCoLaus serology data

**Characteristic**	**UK Biobank**	**(%)**	**CoLaus/PsyCoLaus**	**(%)**
**Age at recruitment**				
30-39	0	0.00	476	11.29
40-49	2250	23.41	1280	30.36
50-59	3145	32.72	1159	27.49
60-69	4158	43.26	991	23.51
70+	58	0.60	310	7.35
**Sex**				
Male	4236	44.07	1946	46.16
Female	5375	55.93	2270	53.84
**Reported ethnicity**				
White	9061	94.28	4216	100
Asian	234	2.43		
Black	139	1.45		
Other	134	1.39		
Not reported/missing	43	0.45		

Table S4: Phenotype definitions of ICD-10 derived phenotypes

See Excel spreadsheet

Table S5: Phenotype definitions of self-reported phenotypes

Phenotype group	Phenotype	Cases	Controls	UK Biobank code(s)
Allergy	Allergy (unspecified)	3584	369427	1374
Allergy	Asthma	57509	315502	1111
Allergy	Eczema	14021	358990	1452
Allergy	Hayfever	30537	342474	1387
Infection	Diphtheria	81	298210	1574
Infection	EBV	1035	298210	1567
Infection	*Helicobacter pylori*	1532	298210	1442
Infection	Hepatitis A	252	298210	1578
Infection	Hepatitis C	135	298210	1580
Infection	HSV	260	298210	1575
Infection	Measles	5560	298210	1568
Infection	Mumps	3384	298210	1569
Infection	Pertussis	1282	298210	1572
Infection	Polio	402	298210	1526
Infection	Rubella	1996	298210	1570
Infection	Scarlet fever	1012	298210	1677
Infection	*Staphylococcus aureus* (MRSA)	72	298210	1566
Infection	Tonsillitis	4893	298210	1598
Infection	Tuberculosis	2581	298210	1440
Infection	Typhoid fever	60	298210	1577
Infection	VZV (Chickenpox)	6886	298210	1571
Infection	VZV (Combined)	7475	298210	1571, 1573, 1674
Infection	VZV (Shingles)	1067	298210	1573
Inflammation	Coronary artery disease	2015	370977	1082
Inflammation	COPD	1895	371116	1112
Inflammation	Diabetes (type 1)	478	372533	1222
Inflammation	Multiple sclerosis	1738	371273	1261
Inflammation	Myocardial infarction	11592	361400	1075
Inflammation	Parkinson's	901	372110	1262
Inflammation	Psoriasis	5861	367150	1453
Inflammation	Rheumatoid arthritis	5713	367298	1464
Inflammation	Rosacea	1010	372001	1660
Inflammation	Sarcoidosis	1030	371981	1371
Inflammation	Systemic lupus erythematosus	636	372375	1381
Inflammation	Ulcerative colitis	2635	370357	1463
Other	Abnormal cervical smear	804	201181	1663
Other	Alzheimer's	137	372874	1263
Other	Cervical abnormality	1225	200760	1554, 1663
Other	Cervical intraepithelial neoplasia	433	201552	1554
Infection	Additional codes included in super-phenotype	NA	NA	1156, 1196, 1244, 1246, 1247, 1274, 1412, 1416, 1418, 1439, 1514, 1515, 1576, 1579, 1581, 1582, 1594, 1657, 1676, 1678

Table S6: Top variants for genomic regions excluding the extended MHC region associated with antibody responses to 45 pathogen-derived antigens. Betas are reported relative to the minor allele.

Chr	Region	Antigen	SNP	MAF	Beta	SE	P
3	68638922-69293121	*C. trachomatis* (mompA)	3:68985092_TAGTGACA_T	0.030	0.279	0.045	6.50×10^{-10}
		C. trachomatis (mompD)	3:68985092_TAGTGACA_T	0.030	0.262	0.045	7.10×10^{-9}
4	103390496-104199922	EBV (EA-D)	rs230493	0.355	-0.068	0.015	3.60×10^{-6}
		EBV (VCAp18)	rs1289231	0.077	0.126	0.028	7.00×10^{-6}
		EBV (ZEBRA)	rs3755867	0.332	-0.076	0.015	3.60×10^{-7}
		EBV (ZEBRA)	rs4699031	0.332	-0.076	0.015	3.60×10^{-7}
		HHV-6 (IE1A)	4:103406914_TA_T	0.366	-0.096	0.015	1.30×10^{-10}
		HHV-7 (U14)	rs4648052	0.375	-0.072	0.014	5.70×10^{-7}
		HIV (env)	rs567727578	0.468	-0.068	0.015	4.00×10^{-6}
		HPV18 (L1)	rs4648052	0.375	-0.068	0.015	3.90×10^{-6}
		HTLV-1 (env)	rs4648068	0.330	-0.075	0.015	1.00×10^{-6}
		HTLV-1 (env)	rs4648058	0.330	-0.075	0.015	1.00×10^{-6}
		KSHV (K8.1)	rs10013613	0.403	-0.074	0.014	2.50×10^{-7}
		KSHV (K8.1)	rs74462352	0.397	-0.075	0.014	2.50×10^{-7}
		T. gondii (sag1)	rs10013613	0.403	-0.067	0.014	3.20×10^{-6}
5	138114233-139053851	MCV (VP1)	rs13181561	0.265	0.155	0.016	4.90×10^{-22}
7	150011299-150484264	HTLV-1 (gag)	rs12534190	0.230	0.166	0.017	9.90×10^{-23}
11	118762073-118811315	HHV-7 (U14)	rs75438046	0.028	-0.278	0.043	9.80×10^{-11}
14	106624768-107228049	HCV (NS3)	rs75801933	0.066	0.145	0.029	6.80×10^{-7}
		HIV (env)	rs4977158	0.084	0.163	0.036	4.80×10^{-6}
		HIV (gag)	rs10150853	0.099	0.140	0.030	2.30×10^{-6}
		HIV (gag)	rs10138691	0.099	0.140	0.030	2.30×10^{-6}
		HPV16 (E6)	rs2337939	0.067	0.191	0.030	1.60×10^{-10}
		HPV16 (E7)	rs199944736	0.150	-0.113	0.021	5.90×10^{-8}
		HPV16 (E7)	rs201829121	0.150	-0.113	0.021	$5.90\times10\text{-}^{8}$
		HPV16 (L1)	rs4774155	0.070	0.136	0.028	1.60×10^{-6}
		HPV18 (L1)	rs75801933	0.066	0.134	0.029	4.10×10^{-6}
		HTLV-1 (env)	rs75801933	0.066	0.154	0.029	1.40×10^{-7}
		T. gondii (p22)	rs4977158	0.084	0.176	0.036	7.70×10^{-7}
		T. gondii (sag1)	rs4977158	0.084	0.213	0.035	1.70×10^{-9}

19	49097126-49282803	BKV (VP1)	rs681343	0.489	-0.123	0.014	3.80×10^{-18}
		JCV (VP1)	rs681343	0.489	-0.134	0.014	1.70×10^{-21}
22	23154058-23220565	*H. pylori* (UreA)	22:23169419_GT_G	0.173	-0.119	0.019	9.00×10^{-10}
22	41424903-42698602	HHV-7 (U14)	rs35211694	0.245	0.117	0.019	4.40×10^{-10}

Table S7: Top variants within the extended MHC region (chr6:25,384,361-34,366,455) associated with antibody responses to 45 pathogen-derived antigens. Betas are reported relative to the minor allele.

**Antigen**	**SNP**	**MAF**	**Beta**	**SE**	**P**
BKV (VP1)	rs4713573	0.324	0.079	0.016	4.70×10^{-7}
CMV (pp150)	rs9275015	0.300	-0.086	0.017	2.30×10^{-7}
CMV (pp52)	rs9270798	0.232	0.096	0.017	1.00×10^{-8}
C. trachomatis (mompA)	rs71536538	0.273	-0.081	0.017	3.50×10^{-6}
C. trachomatis (mompD)	rs4367411	0.201	-0.098	0.019	1.80×10^{-7}
C. trachomatis (TarpDF2)	rs112438356	0.098	-0.139	0.026	5.00×10^{-8}
C. trachomatis (TarpDF2)	rs112327275	0.098	-0.139	0.026	5.00×10^{-8}
EBV (EA-D)	rs2395192	0.460	-0.163	0.014	3.40×10^{-31}
EBV (EBNA1)	rs530423766	0.433	-0.324	0.015	6.90×10^{-97}
EBV (VCAp18)	rs9264759	0.208	0.158	0.018	4.00×10^{-19}
EBV (ZEBRA)	rs9274704	0.278	0.306	0.016	2.70×10^{-86}
HBV (HBc)	rs112275579	0.365	0.079	0.017	3.80×10^{-6}
HBV (HBe)	rs9266092	0.391	0.070	0.015	4.90×10^{-6}
HCV (NS3)	rs3997848	0.323	0.082	0.015	1.20×10^{-7}
HHV-6 (IE1A)	rs9265967	0.139	-0.134	0.021	2.30×10^{-10}
HHV-6 (IE1B)	rs28383304	0.145	0.146	0.021	1.30×10^{-12}
HHV-6 (p101k)	rs4959072	0.219	0.120	0.017	5.40×10^{-12}
HHV-7 (U14)	rs9270140	0.143	-0.163	0.021	6.20×10^{-15}
HIV (env)	rs41288885	0.059	0.153	0.031	1.20×10^{-6}
HIV (env)	6:32748889_CT_C	0.046	0.185	0.038	1.20×10^{-6}
HIV (gag)	rs3134974	0.445	-0.072	0.015	2.10×10^{-6}
HPV16 (E6)	rs35445446	0.168	0.087	0.019	6.60×10^{-6}
HPV16 (E7)	rs78274956	0.495	-0.130	0.016	1.20×10^{-16}
HPV16 (L1)	rs548129848	0.417	0.088	0.017	1.00×10^{-7}
HPV18 (L1)	rs6930081	0.468	-0.066	0.015	5.60×10^{-6}
HPV18 (L1)	rs6929819	0.468	-0.066	0.014	5.60×10^{-6}
HSV-1 (1gG)	rs116135626	0.187	0.151	0.025	1.20×10^{-9}
HSV-2 (2mgG)	rs36152293	0.337	0.107	0.018	4.60×10^{-9}
HTLV-1 (env)	rs115675626	0.088	-0.154	0.027	2.20×10^{-8}
HTLV-1 (gag	rs9271727	0.252	0.164	0.017	2.20×10^{-22}
H. pylori (CagA)	rs71534592	0.121	-0.172	0.032	5.60×10^{-8}
H. pylori (Catalase)	rs9270641	0.199	0.104	0.020	3.90×10^{-7}
H. pylori (HP1564)	rs9268541	0.058	0.146	0.030	1.30×10^{-6}
H. pylori (VacA)	rs35186928	0.370	-0.075	0.015	3.30×10^{-7}
JCV (VP1)	rs71538510	0.173	-0.323	0.019	8.50×10^{-66}
KSHV (K8.1)	rs2844535	0.274	-0.076	0.016	1.80×10^{-6}
MCV (VP1)	rs9269771	0.274	-0.249	0.017	2.70×10^{-48}
T. gondii (sag1)	rs144947706	0.369	-0.113	0.016	1.40×10^{-12}
VZV (gE/gI)	rs9273325	0.174	0.221	0.019	7.10×10^{-33}

Table S8: Top variants associated with antibody response in the UK Biobank and CoLaus datasets following meta-analysis

		UK Biobank			CoLaus			Meta-analysis (fixed-effects)		
**Antigen**	**SNP ID (UKB)**	**beta**	**SE**	**P**	**beta**	**SE**	**P**	**beta**	**SE**	**P**
EBV (EAD)	rs2395192	-0.163	0.014	3.40×10^{-31}	-0.111	0.027	4.83×10^{-5}	-0.152	0.012	3.54×10^{-34}
EBV (ZEBRA)	rs9274728	-0.306	0.016	3.70×10^{-86}	-0.323	0.032	1.83×10^{-24}	-0.309	0.014	8.74×10^{-109}
HHV6 (IE1A)	rs8192589	0.126	0.020	4.70×10^{-10}	0.092	0.037	1.41×10^{-2}	0.118	0.018	2.98×10^{-11}
HHV7 (U14)	6:32593225_CAG_C	0.198	0.026	7.20×10^{-14}	0.218	0.044	5.77×10^{-7}	0.203	0.023	2.44×10^{-19}
KSHV (K8.1)	rs2372093	-0.080	0.018	1.20×10^{-5}	-0.082	0.029	5.46×10^{-3}	-0.080	0.016	2.10×10^{-7}
T. gondii (sag1)	rs7754119	0.103	0.015	1.10×10^{-11}	0.053	0.023	2.04×10^{-2}	0.088	0.013	3.95×10^{-12}

Table S9: Published associations between *NFKB1* and disease-related traits

Published trait	Trait type	Ancestry	SNP	R^2 (rs28362491)	Study size (cases)	P	OR (del)	95% LCI	95% UCI	Ref
Allergic rhinitis	Allergy	European	rs12509403	0.556	891,367 (120,482)	1.17×10^{-15}	0.960	0.950	0.97	13
Allergic sensitisation	Allergy	European	rs4648050	0.572	24,481 (8,040)	2.03×10^{-8}	0.880	0.840	0.92	13
Combined hayfever and eczema	Allergy	White British	rs230507	0.776	346,545 (106,772)	1.04×10^{-9}	0.963	0.951	0.974	14
Tonsillectomy	Infection	European	rs230523	0.759	173,421 (60,098)	4.54×10^{-14}	1.070	1.060	1.08	15
ARDS* (<65yrs)	Inflammation	European	rs28362491	NA	NR	3.00×10^{-2}	0.106	0.014	0.825	16
Ankylosing spondylitis	Inflammation	European	rs3774937	0.751	86,475 (52,262)	7.83×10^{-10}	1.120	1.080	1.16	17
Behçet's disease	Inflammation	Turkish	rs28362491	NA	270 (89)	4.00×10^{-3}	0.556	0.380	0.811	18
Coronary artery disease	Inflammation	European	rs28362491	NA	220 (120)	1.50×10^{-2}	2.880	1.210	6.84	19
Coronary artery disease	Inflammation	India	rs28362491	NA	830 (600)	2.90×10^{-2}	1.260	1.030	1.55	20
Coronary artery disease	Inflammation	Uygur	rs28362491	NA	2020 (960)	<0.001	1.581	1.222	2.046	21
Left ventricular dysfunction in CAD*	Inflammation	India	rs28362491	NA	600 (190)	7.00×10^{-3}	2.340	1.310	4.17	20
Lung injury score in ARDS*	Inflammation	European	rs28362491	NA	103 (77)	3.00×10^{-4}	3.700	1.800	7.9	22
Mouth Ulcers	Inflammation	European	rs4699030	0.863	816,850 (145,377)	5.91×10^{-11}	1.030	1.020	1.04	23
Myocardial infarction	Inflammation	European	rs28362491	NA	253 (86)	<0.001	0.304	0.177	0.522	24
Primary biliary cholangitis	Inflammation	Japanese	rs230534	0.745	2,886 (1,381)	1.50×10^{-7}	1.340	1.200	1.49	25
Primary sclerosing cholangitis	Inflammation	European	rs3774937	0.751	37,621 (3,408)	6.11×10^{-9}	1.167	1.107	1.229	17
Systemic sclerosis	Inflammation	Iran/Turkey	rs4648133	0.572	2107 (764)	3.11×10^{-7}	1.470	1.270	1.7	26
TR schizophrenia*	Inflammation	Han Chinese	rs230529	0.897	1,610 (804)	1.74×10^{-7}	1.450	1.260	1.66	27
Ulcerative colitis	Inflammation	European	rs3774959	0.698	47,560 (17,865)	3.66×10^{-12}	1.118	1.077	1.159	28
Ulcerative colitis	Inflammation	European	rs3774937	0.751	48,626 (14,413)	1.33×10^{-11}	1.107	1.075	1.142	17
Ulcerative colitis	Inflammation	European	rs28362491	NA	1,152 (350)	4.30×10^{-3}	1.570	1.140	2.16	29
Ulcerative colitis	Inflammation	European	rs28362491	NA	282 (127)	1.10×10^{-2}	2.510	1.280	4.92	30
Cancer risk	Other	European/ Chinese	rs28362491	NA	43,000 (18222)	2.00×10^{-3}	0.890	0.830	0.96	31

Published trait		Ancestry	SNP	R^2	Study size (cases)	P	Beta (del)	SE		Ref
Albumin/Globulin Ratio	Other	Japanese	rs60371688	0.850	98,626	1.49×10^{-12}	0.036	0.005		32
Asthma	Allergy	White British	rs59123962	0.697	458,699	7.50×10^{-4}	-0.002	0.001		33
Combined hayfever, allergic rhinitis, and eczema	Allergy	White British	rs230504	0.777	458,699	6.90×10^{-13}	-0.007	0.001		33
Non-albumin protein	Other	Japanese	rs1585213	0.949	98,538	1.47×10^{-15}	-0.037	0.005		32
Immunogobulin levels ((A + G)/M)	Other	European	rs4648052	0.700	19,219	3.00×10^{-7}	-0.060	NR		34
Immunogobulin levels (A - M)	Other	European	rs4648052	0.700	19,219	2.30×10^{-7}	-0.070	NR		34

*ARDS: Acute respiratory distress syndrome; CAD: Coronary artery disease; TR: Treatment resistant

Table S10: Association statistics for rs28362491 and self-reported and ICD-10 derived phenotypes after exclusion of phenotypes where the regression model was poorly fitted

**Phenotype group**	**Phenotype**	**OR**	**95% CI**	**P**
Allergy	Allergic dermatitis (ICD)	0.957	0.747-1.227	0.729
Allergy	Allergy (unspecified) (SR)	0.986	0.940-1.033	0.547
Allergy	Asthma	0.993	0.982-1.005	0.280
Allergy	Atopic dermatitis (ICD)	0.850	0.637-1.133	0.267
Allergy	Eczema (SR)	0.981	0.957-1.005	0.123
Allergy	Hayfever	0.965	0.949-0.981	3.57×10^{-5}
Allergy	Seborrhoeic dermatitis (ICD)	0.817	0.601-1.111	0.197
Infection	*Aspergillus spp.* (ICD)	0.946	0.786-1.140	0.560
Infection	*Campylobacter spp.* (ICD)	1.001	0.885-1.132	0.986
Infection	*Candida spp.* (ICD)	0.993	0.947-1.042	0.778
Infection	*Clostridium difficile* (ICD)	1.018	0.930-1.115	0.697
Infection	CMV (ICD)	1.094	0.890-1.345	0.395
Infection	*Corynebacterium diphtheriae*	0.912	0.667-1.248	0.566
Infection	EBV	1.067	0.979-1.162	0.143
Infection	*Enterobius vermicularis* (ICD)	1.110	0.868-1.420	0.406
Infection	Enterovirus (ICD)	0.989	0.698-1.399	0.948
Infection	*Escherichia coli* (ICD)	1.026	0.984-1.071	0.230
Infection	*Giardia lamblia* (ICD)	1.026	0.708-1.485	0.894
Infection	*Haemophilus influenzae* (ICD)	1.012	0.896-1.143	0.850
Infection	*Helicobacter pylori*	1.022	0.974-1.073	0.371
Infection	Hepatitis A	1.027	0.882-1.195	0.734
Infection	Hepatitis C	1.122	0.983-1.281	0.087
Infection	HSV	1.032	0.926-1.149	0.570
Infection	Influenza (ICD)	1.120	1.000-1.253	4.90×10^{-2}
Infection	*Klebsiella pneumoniae* (ICD)	1.053	0.947-1.172	0.339
Infection	*Legionella spp*. (ICD)	1.524	1.097-2.118	1.21×10^{-2}
Infection	Measles	0.978	0.941-1.016	0.247
Infection	MOCV (ICD)	1.056	0.778-1.434	0.726
Infection	Mumps	0.996	0.948-1.045	0.862
Infection	*Neisseria meningitidis* (ICD)	0.704	0.509-0.974	3.43×10^{-2}
Infection	Norovirus (ICD)	1.208	0.966-1.511	0.098
Infection	Papillomavirus (ICD)	1.095	1.019-1.177	1.38×10^{-2}
Infection	Pertussis	0.984	0.909-1.065	0.693
Infection	*Pneumocystis jirovecii* (ICD)	1.007	0.735-1.381	0.963
Infection	Polio	0.958	0.835-1.100	0.544
Infection	*Proteus spp*. (ICD)	1.143	0.996-1.311	0.057
Infection	*Staphylococcus aureus*	1.012	0.964-1.062	0.635
Infection	Streptococcus A	1.072	0.991-1.160	0.082
Infection	Streptococcus B (ICD)	1.063	0.924-1.222	0.391
Infection	Streptococcus D (ICD)	0.966	0.792-1.177	0.729
Infection	*Streptococcus pneumoniae* (ICD)	1.068	0.956-1.193	0.246
Infection	Tonsillitis (SR)	0.976	0.937-1.016	0.235
Infection	Tuberculosis (SR)	0.984	0.931-1.041	0.576
Infection	VZV (Chicken pox)	0.983	0.950-1.017	0.313
Infection	VZV (Combined)	0.994	0.963-1.026	0.704
Infection	VZV (Shingles)	1.028	0.961-1.099	0.421
Inflammation (Other)	Coronary artery disease	1.004	0.989-1.019	0.595

Inflammation (Other)	COPD	1.016	0.991-1.042	0.206
Inflammation (Other)	Encephalomyelitis (ICD)	1.048	0.874-1.258	0.611
Inflammation (Other)	Inflammatory polyarthropathy (ICD)	0.864	0.687-1.087	0.213
Inflammation (Other)	Motor neuron disease (ICD)	1.062	0.913-1.236	0.435
Inflammation (Chronic)	Multiple Sclerosis	1.047	0.985-1.113	0.144
Inflammation (Other)	Myocardial infarction	1.006	0.985-1.028	0.550
Inflammation (Other)	Parkinson's	1.044	0.981-1.112	0.177
Inflammation (Chronic)	Psoriasis	1.044	1.005-1.083	2.55×10^{-2}
Inflammation (Chronic)	Rheumatoid Arthritis	0.986	0.955-1.018	0.389
Inflammation (Other)	Rosacea	0.979	0.908-1.055	0.577
Inflammation (Other)	Ulcerative colitis	1.079	1.037-1.124	2.15×10^{-4}
Other	Abnormal cervical smear (SR)	0.990	0.896-1.093	0.836
Other	Alzheimer's	1.109	1.012-1.215	2.65×10^{-2}
Other	Cervical abnormality (SR)	1.055	0.973-1.144	0.196
Other	Cervical intraepithelial neoplasia (SR)	1.166	1.020-1.334	2.48×10^{-2}
	Allergy	0.988	0.978-0.998	1.84×10^{-2}
	Chronic inflammation	1.020	0.998-1.042	0.075
	Infection	1.014	1.001-1.027	3.31×10^{-2}

Table S11: Association statistics for rs28362491 and mortality associated with allergy, infection, or inflammation

Phenotype	Cases	Controls	OR	95% CI	P
Allergy	131	25205	0.846	0.658-1.088	0.192
Infection	1244	24092	1.065	0.982-1.155	0.129
Inflammation	4421	20915	1.038	0.991-1.087	0.117

Table S12: Association statistics for rs28362491 and blood cell counts

Phenotype	Beta	SE	P
Leukocyte count	-9.50×10^{-5}	1.97×10^{-3}	0.960
Lymphocyte count	1.98×10^{-2}	1.95×10^{-3}	3.30×10^{-24}
Basophil count	-1.25×10^{-2}	2.08×10^{-3}	2.00×10^{-9}
Eosinophil count	6.49×10^{-3}	1.96×10^{-3}	9.30×10^{-4}
Monocyte count	-1.50×10^{-2}	1.89×10^{-3}	2.70×10^{-15}
Neutrophil count	-7.27×10^{-3}	1.99×10^{-3}	2.50×10^{-4}
RBC count	-1.04×10^{-2}	1.67×10^{-3}	4.80×10^{-10}
Platelet count	-5.79×10^{-3}	1.79×10^{-3}	1.30×10^{-3}

Table S13: Colocalisation results for GWAS and eQTL phenotype pairs

**Phenotype 1**	**Phenotype 2**	**Lead SNP 1**	**Lead SNP 2**	**PP H0**	**PP H1**	**PP H2**	**PP H3**	**PP H4**
EBV (EA-D)	EBV (ZEBRA)	rs230493	rs4648058	2.02×10^{-4}	3.31×10^{-3}	1.01×10^{-2}	1.64×10^{-1}	8.22×10^{-1}
EBV (ZEBRA)	HHV-6 (IE1A)	rs4648058	rs1598859	1.28×10^{-8}	6.42×10^{-7}	2.88×10^{-3}	1.43×10^{-1}	8.54×10^{-1}
HHV-6 (IE1A)	HHV-7 (U14)	rs1598859	rs4648052	9.53×10^{-8}	2.15×10^{-2}	1.82×10^{-6}	4.09×10^{-1}	5.69×10^{-1}
HHV-7 (U14)	HIV (Env)	rs4648052	rs10013613	4.71×10^{-4}	9.01×10^{-3}	6.80×10^{-3}	1.28×10^{-1}	8.55×10^{-1}
HIV (Env)	HPV18 (L1)	rs10013613	rs4648052	9.18×10^{-4}	1.33×10^{-2}	9.37×10^{-3}	1.34×10^{-1}	8.43×10^{-1}
HPV18 (L1)	HTLV-1 (Env)	rs4648052	rs4648058	5.38×10^{-4}	5.49×10^{-3}	1.31×10^{-2}	1.32×10^{-1}	8.49×10^{-1}
HTLV-1 (Env)	KSHV (K8.1)	rs4648058	rs10013613	7.84×10^{-5}	1.91×10^{-3}	9.39×10^{-3}	2.27×10^{-1}	7.61×10^{-1}
KSHV (K8.1)	*T. gondii* (sag1)	rs10013613	rs10013613	7.81×10^{-5}	9.36×10^{-3}	8.16×10^{-4}	9.59×10^{-2}	8.94×10^{-1}
HHV-6 (IE1A)	Basophil count	rs1598859	rs2168805	1.33×10^{-10}	2.99×10^{-5}	1.70×10^{-6}	3.81×10^{-1}	6.19×10^{-1}
HHV-6 (IE1A)	Eosinophil count	rs1598859	rs230507	3.71×10^{-21}	8.35×10^{-16}	6.10×10^{-7}	1.36×10^{-1}	8.64×10^{-1}
HHV-6 (IE1A)	Lymphocyte count	rs1598859	rs35680095	7.00×10^{-18}	1.58×10^{-12}	4.44×10^{-6}	9.99×10^{-1}	1.45×10^{-7}
HHV-6 (IE1A)	Monocyte count	rs1598859	rs980455	4.04×10^{-17}	9.09×10^{-12}	1.33×10^{-6}	2.97×10^{-1}	7.03×10^{-1}
HHV-6 (IE1A)	Red blood cell count	rs1598859	rs230539	3.15×10^{-12}	7.08×10^{-7}	9.36×10^{-7}	2.09×10^{-1}	7.91×10^{-1}
HHV-6 (IE1A)	Platelet count	rs1598859	rs62328536	2.49×10^{-6}	5.60×10^{-1}	4.75×10^{-7}	1.06×10^{-1}	3.33×10^{-1}
HHV-6 (IE1A)	eQTL: Monocytes (unstim)	rs1598859	rs72696119	5.34×10^{-7}	1.20×10^{-1}	1.26×10^{-6}	2.82×10^{-1}	5.98×10^{-1}
HHV-6 (IE1A)	eQTL: Monocytes (LPS2)	rs1598859	rs4698856	1.39×10^{-8}	3.14×10^{-3}	1.53×10^{-6}	3.43×10^{-1}	6.53×10^{-1}
HHV-6 (IE1A)	eQTL: Monocytes (LPS24)	rs1598859	rs28882677	1.60×10^{-8}	3.61×10^{-3}	2.10×10^{-6}	4.72×10^{-1}	5.25×10^{-1}
HHV-6 (IE1A)	eQTL: Monocytes (IFN)	rs1598859	rs4648055	9.37×10^{-12}	2.11×10^{-6}	6.76×10^{-7}	1.50×10^{-1}	8.50×10^{-1}
HHV-6 (IE1A)	eQTL: Neutrophils	rs1598859	rs4698857	6.31×10^{-8}	1.42×10^{-2}	7.72×10^{-7}	1.72×10^{-1}	8.14×10^{-1}
HHV-6 (IE1A)	eQTL: Natural killer cells	rs1598859	rs72696119	3.90×10^{-8}	8.78×10^{-3}	1.18×10^{-6}	2.63×10^{-1}	7.28×10^{-1}
Monocyte count	eQTL: Monocytes (unstim)	rs980455	rs72696119	1.44×10^{-12}	4.73×10^{-2}	3.40×10^{-12}	1.10×10^{-1}	8.43×10^{-1}
Monocyte count	eQTL: Monocytes (LPS2)	rs980455	rs4698856	2.75×10^{-14}	9.03×10^{-4}	3.02×10^{-12}	9.74×10^{-2}	9.02×10^{-1}
Monocyte count	eQTL: Monocytes (LPS24)	rs980455	rs28882677	2.15×10^{-14}	7.05×10^{-4}	2.81×10^{-12}	9.05×10^{-2}	9.09×10^{-1}
Monocyte count	eQTL: Monocytes (IFN)	rs980455	rs4648055	1.44×10^{-16}	4.73×10^{-6}	1.04×10^{-11}	3.40×10^{-1}	6.60×10^{-1}
Neutrophil count	eQTL: Neutrophils	rs2903281	rs4698857	2.33×10^{-3}	3.22×10^{-2}	2.84×10^{-2}	3.93×10^{-1}	5.44×10^{-1}
Lymphocyte count	eQTL: Natural killer cells	rs35680095	rs72696119	5.05×10^{-14}	3.20×10^{-2}	1.52×10^{-12}	9.65×10^{-1}	2.78×10^{-3}

*PP: Posterior probability

Table S14: Two-sample Mendelian Randomisations results for causal effect of blood cell counts on antibody response

Exposure	Outcome	Coef.Estimate	SE	P
RBC count	EBV (EAD)	5.696	1.370	3.21×10^{-5}
RBC count	EBV (ZEBRA)	5.955	1.364	1.27×10^{-5}
RBC count	HHV6 (IE1A)	8.292	1.397	2.92×10^{-9}
RBC count	HHV-7 (U14)	5.526	1.362	4.98×10^{-5}
RBC count	HIV (env)	5.905	1.398	2.39×10^{-5}
RBC count	HPV-18 (L1)	5.706	1.395	4.32×10^{-5}
RBC count	HTLV-1 (env)	5.660	1.396	5.05×10^{-5}
RBC count	KSHV (K8.1)	6.676	1.386	1.46×10^{-6}
RBC count	*T. gondii* (sag1)	6.065	1.384	1.17×10^{-5}
Basophil count	EBV (EAD)	4.742	1.140	3.21×10^{-5}
Basophil count	EBV (ZEBRA)	4.958	1.136	1.27×10^{-5}
Basophil count	HHV6 (IE1A)	6.903	1.163	2.92×10^{-9}
Basophil count	HHV-7 (U14)	4.601	1.134	4.98×10^{-5}
Basophil count	HIV (env)	4.916	1.164	2.39×10^{-5}
Basophil count	HPV-18 (L1)	4.750	1.162	4.32×10^{-5}
Basophil count	HTLV-1 (env)	4.712	1.163	5.05×10^{-5}
Basophil count	KSHV (K8.1)	5.558	1.154	1.46×10^{-6}
Basophil count	*T. gondii* (sag1)	5.049	1.152	1.17×10^{-5}
Neutrophil count	EBV (EAD)	8.144	1.958	3.21×10^{-5}
Neutrophil count	EBV (ZEBRA)	8.515	1.950	1.27×10^{-5}
Neutrophil count	HHV6 (IE1A)	11.856	1.997	2.92×10^{-9}
Neutrophil count	HHV-7 (U14)	7.902	1.948	4.98×10^{-5}
Neutrophil count	HIV (env)	8.444	1.998	2.39×10^{-5}
Neutrophil count	HPV-18 (L1)	8.159	1.995	4.32×10^{-5}
Neutrophil count	HTLV-1 (env)	8.093	1.997	5.05×10^{-5}
Neutrophil count	KSHV (K8.1)	9.545	1.982	1.46×10^{-6}
Neutrophil count	*T. gondii* (sag1)	8.671	1.978	1.17×10^{-5}
Monocyte count	EBV (EAD)	3.957	0.952	3.21×10^{-5}
Monocyte count	EBV (ZEBRA)	4.137	0.948	1.27×10^{-5}
Monocyte count	HHV6 (IE1A)	5.761	0.970	2.92×10^{-9}
Monocyte count	HHV-7 (U14)	3.840	0.947	4.98×10^{-5}
Monocyte count	HIV (env)	4.103	0.971	2.39×10^{-5}
Monocyte count	HPV-18 (L1)	3.964	0.969	4.32×10^{-5}
Monocyte count	HTLV-1 (env)	3.932	0.970	5.05×10^{-5}
Monocyte count	KSHV (K8.1)	4.638	0.963	1.46×10^{-6}
Monocyte count	*T. gondii* (sag1)	4.214	0.961	1.17×10^{-5}
Eosinophil count	EBV (EAD)	-9.118	2.193	3.21×10^{-5}
Eosinophil count	EBV (ZEBRA)	-9.533	2.184	1.27×10^{-5}
Eosinophil count	HHV6 (IE1A)	-13.274	2.236	2.92×10^{-9}
Eosinophil count	HHV-7 (U14)	-8.847	2.181	4.98×10^{-5}
Eosinophil count	HIV (env)	-9.453	2.237	2.39×10^{-5}
Eosinophil count	HPV-18 (L1)	-9.134	2.233	4.32×10^{-5}
Eosinophil count	HTLV-1 (env)	-9.061	2.236	5.05×10^{-5}
Eosinophil count	KSHV (K8.1)	-10.687	2.219	1.46×10^{-6}

Eosinophil count	*T. gondii* (sag1)	-9.709	2.215	$1.17{\times}10^{-5}$
Lymphocyte count	EBV (EAD)	-2.988	0.719	$3.21{\times}10^{-5}$
Lymphocyte count	EBV (ZEBRA)	-3.124	0.716	$1.27{\times}10^{-5}$
Lymphocyte count	HHV6 (IE1A)	-4.350	0.733	$2.92{\times}10^{-9}$
Lymphocyte count	HHV-7 (U14)	-2.899	0.715	$4.98{\times}10^{-5}$
Lymphocyte count	HIV (env)	-3.098	0.733	$2.39{\times}10^{-5}$
Lymphocyte count	HPV-18 (L1)	-2.993	0.732	$4.32{\times}10^{-5}$
Lymphocyte count	HTLV-1 (env)	-2.969	0.733	$5.05{\times}10^{-5}$
Lymphocyte count	KSHV (K8.1)	-3.502	0.727	$1.46{\times}10^{-6}$
Lymphocyte count	*T. gondii* (sag1)	-3.181	0.726	$1.17{\times}10^{-5}$
Platelet count	EBV (EAD)	10.227	2.459	$3.21{\times}10^{-5}$
Platelet count	EBV (ZEBRA)	10.693	2.449	$1.27{\times}10^{-5}$
Platelet count	HHV6 (IE1A)	14.889	2.508	$2.92{\times}10^{-9}$
Platelet count	HHV-7 (U14)	9.923	2.446	$4.98{\times}10^{-5}$
Platelet count	HIV (env)	10.604	2.510	$2.39{\times}10^{-5}$
Platelet count	HPV-18 (L1)	10.246	2.505	$4.32{\times}10^{-5}$
Platelet count	HTLV-1 (env)	10.163	2.507	$5.05{\times}10^{-5}$
Platelet count	KSHV (K8.1)	11.987	2.489	$1.46{\times}10^{-6}$
Platelet count	*T. gondii* (sag1)	10.890	2.485	$1.17{\times}10^{-5}$

Table S15: Power calculations for Mendelian Randomisation analyses for causal effect of blood cell counts on antibody response

Exposure	Outcome	Power for full cohort
RBC count	EBV (EAD)	99.90%
RBC count	EBV (ZEBRA)	100.00%
RBC count	HHV6 (IE1A)	100.00%
RBC count	HHV-7 (U14)	99.80%
RBC count	HIV (env)	99.90%
RBC count	HPV-18 (L1)	99.90%
RBC count	HTLV-1 (env)	99.90%
RBC count	KSHV (K8.1)	100.00%
RBC count	*T. gondii* (sag1)	100.00%
Basophil count	EBV (EAD)	98.20%
Basophil count	EBV (ZEBRA)	98.90%
Basophil count	HHV6 (IE1A)	100.00%
Basophil count	HHV-7 (U14)	97.60%
Basophil count	HIV (env)	98.70%
Basophil count	HPV-18 (L1)	98.70%
Basophil count	HTLV-1 (env)	98.10%
Basophil count	KSHV (K8.1)	99.70%
Basophil count	*T. gondii* (sag1)	99.70%
Neutrophil count	EBV (EAD)	98.80%
Neutrophil count	EBV (ZEBRA)	99.30%
Neutrophil count	HHV6 (IE1A)	100.00%
Neutrophil count	HHV-7 (U14)	98.40%
Neutrophil count	HIV (env)	99.20%
Neutrophil count	HPV-18 (L1)	98.80%
Neutrophil count	HTLV-1 (env)	98.70%
Neutrophil count	KSHV (K8.1)	99.90%
Neutrophil count	*T. gondii* (sag1)	99.40%
Monocyte count	EBV (EAD)	99.40%
Monocyte count	EBV (ZEBRA)	99.70%
Monocyte count	HHV6 (IE1A)	100.00%
Monocyte count	HHV-7 (U14)	99.10%
Monocyte count	HIV (env)	99.60%
Monocyte count	HPV-18 (L1)	99.40%
Monocyte count	HTLV-1 (env)	99.30%
Monocyte count	KSHV (K8.1)	99.90%
Monocyte count	*T. gondii* (sag1)	99.70%
Eosinophil count	EBV (EAD)	99.00%
Eosinophil count	EBV (ZEBRA)	99.40%
Eosinophil count	HHV6 (IE1A)	100.00%
Eosinophil count	HHV-7 (U14)	98.60%
Eosinophil count	HIV (env)	99.40%
Eosinophil count	HPV-18 (L1)	99.00%
Eosinophil count	HTLV-1 (env)	98.90%
Eosinophil count	KSHV (K8.1)	99.90%

Eosinophil count	*T. gondii* (sag1)	99.50%
Lymphocyte count	EBV (EAD)	99.10%
Lymphocyte count	EBV (ZEBRA)	99.50%
Lymphocyte count	HHV6 (IE1A)	100.00%
Lymphocyte count	HHV-7 (U14)	98.70%
Lymphocyte count	HIV (env)	99.40%
Lymphocyte count	HPV-18 (L1)	99.10%
Lymphocyte count	HTLV-1 (env)	99.00%
Lymphocyte count	KSHV (K8.1)	99.90%
Lymphocyte count	*T. gondii* (sag1)	99.60%
Platelet count	EBV (EAD)	99.70%
Platelet count	EBV (ZEBRA)	99.80%
Platelet count	HHV6 (IE1A)	100.00%
Platelet count	HHV-7 (U14)	99.50%
Platelet count	HIV (env)	99.80%
Platelet count	HPV-18 (L1)	99.70%
Platelet count	HTLV-1 (env)	99.70%
Platelet count	KSHV (K8.1)	100.00%
Platelet count	*T. gondii* (sag1)	99.90%

Table S16: One-sample Mendelian Randomisations results for causal effect of blood cell counts on infection and inflammation-related disease

Exposure	Outcome	Coef.Estimate	SE	P
RBC count	Influenza (ICD)	2.19×10^{-3}	7.227	1.000
RBC count	*Legionella spp*. (ICD)	1.64×10^{-3}	1.289	0.999
RBC count	*Neisseria meningitidis* (ICD)	7.43×10^{-4}	0.401	0.999
RBC count	Papillomavirus (ICD)	2.09×10^{-3}	1.047	0.998
RBC count	Cervical intraepithelial neoplasia (SR)	-8.29×10^{-3}	1.079	0.994
RBC count	Alzheimer's	1.23×10^{-3}	2.051	1.000
RBC count	Hayfever	1.57×10^{-3}	1.250	0.999
RBC count	Psoriasis	2.65×10^{-3}	13.594	1.000
RBC count	Ulcerative colitis	9.41×10^{-4}	0.646	0.999
RBC count	Allergy	3.03×10^{-2}	0.430	0.944
RBC count	Infection	-4.27×10^{-4}	0.580	0.999
Basophil count	Influenza (ICD)	2.38×10^{-3}	0.819	0.998
Basophil count	*Legionella spp*. (ICD)	1.61×10^{-3}	1.019	0.999
Basophil count	*Neisseria meningitidis* (ICD)	-1.04×10^{-3}	0.543	0.998
Basophil count	Papillomavirus (ICD)	2.81×10^{-3}	0.875	0.997
Basophil count	Cervical intraepithelial neoplasia (SR)	7.32×10^{-3}	4.333	0.999
Basophil count	Alzheimer's	1.94×10^{-3}	0.582	0.997
Basophil count	Hayfever	1.88×10^{-3}	0.507	0.997
Basophil count	Psoriasis	1.89×10^{-3}	0.720	0.998
Basophil count	Ulcerative colitis	1.52×10^{-3}	5.368	1.000
Basophil count	Allergy	1.86×10^{-2}	0.341	0.957
Basophil count	Infection	7.54×10^{-3}	0.509	0.988
Neutrophil count	Influenza (ICD)	2.25×10^{-3}	7.354	1.000
Neutrophil count	*Legionella spp*. (ICD)	3.56×10^{-3}	23.505	1.000
Neutrophil count	*Neisseria meningitidis* (ICD)	1.85×10^{-3}	0.781	0.998
Neutrophil count	Papillomavirus (ICD)	2.73×10^{-3}	0.721	0.997
Neutrophil count	Cervical intraepithelial neoplasia (SR)	-1.03×10^{-3}	0.552	0.999
Neutrophil count	Alzheimer's	2.49×10^{-3}	4.994	1.000
Neutrophil count	Hayfever	1.40×10^{-3}	3.022	1.000
Neutrophil count	Psoriasis	2.02×10^{-3}	1.162	0.999
Neutrophil count	Ulcerative colitis	1.68×10^{-3}	18.612	1.000
Neutrophil count	Allergy	2.81×10^{-2}	3.910	0.994
Neutrophil count	Infection	8.18×10^{-3}	0.846	0.992
Monocyte count	Influenza (ICD)	1.62×10^{-3}	1.022	0.999
Monocyte count	*Legionella spp*. (ICD)	2.07×10^{-3}	32.528	1.000
Monocyte count	*Neisseria meningitidis* (ICD)	1.35×10^{-3}	0.575	0.998
Monocyte count	Papillomavirus (ICD)	2.89×10^{-3}	1.182	0.998
Monocyte count	Cervical intraepithelial neoplasia (SR)	1.14×10^{-3}	0.616	0.999
Monocyte count	Alzheimer's	2.49×10^{-3}	1.568	0.999
Monocyte count	Hayfever	1.99×10^{-3}	6.541	1.000
Monocyte count	Psoriasis	1.96×10^{-3}	0.768	0.998
Monocyte count	Ulcerative colitis	1.34×10^{-3}	1.337	0.999
Monocyte count	Allergy	1.84×10^{-2}	0.753	0.981
Monocyte count	Infection	7.54×10^{-3}	0.497	0.988

Eosinophil count	Influenza (ICD)	2.35×10^{-3}	1.779	0.999
Eosinophil count	*Legionella spp*. (ICD)	1.90×10^{-3}	5.564	1.000
Eosinophil count	*Neisseria meningitidis* (ICD)	1.34×10^{-3}	1.966	0.999
Eosinophil count	Papillomavirus (ICD)	2.81×10^{-3}	7.500	1.000
Eosinophil count	Cervical intraepithelial neoplasia (SR)	3.80×10^{-3}	0.465	0.993
Eosinophil count	Alzheimer's	2.12×10^{-3}	2.278	0.999
Eosinophil count	Hayfever	9.50×10^{-4}	0.456	0.998
Eosinophil count	Psoriasis	1.94×10^{-3}	10.017	1.000
Eosinophil count	Ulcerative colitis	1.45×10^{-3}	1.010	0.999
Eosinophil count	Allergy	2.90×10^{-2}	0.279	0.917
Eosinophil count	Infection	4.50×10^{-3}	5.320	0.999
Lymphocyte count	Influenza (ICD)	2.38×10^{-3}	2.949	0.999
Lymphocyte count	*Legionella spp*. (ICD)	1.94×10^{-3}	0.573	0.997
Lymphocyte count	*Neisseria meningitidis* (ICD)	1.50×10^{-3}	1.009	0.999
Lymphocyte count	Papillomavirus (ICD)	2.97×10^{-3}	1.230	0.998
Lymphocyte count	Cervical intraepithelial neoplasia (SR)	-3.99×10^{-4}	2.735	1.000
Lymphocyte count	Alzheimer's	1.76×10^{-3}	0.403	0.997
Lymphocyte count	Hayfever	2.18×10^{-3}	0.454	0.996
Lymphocyte count	Psoriasis	1.66×10^{-3}	0.784	0.998
Lymphocyte count	Ulcerative colitis	1.56×10^{-3}	0.707	0.998
Lymphocyte count	Allergy	1.96×10^{-2}	0.204	0.923
Lymphocyte count	Infection	-3.20×10^{-4}	0.476	0.999
Platelet count	Influenza (ICD)	2.08×10^{-3}	0.765	0.998
Platelet count	*Legionella spp*. (ICD)	1.75×10^{-3}	67.426	1.000
Platelet count	*Neisseria meningitidis* (ICD)	9.03×10^{-4}	0.545	0.999
Platelet count	Papillomavirus (ICD)	2.39×10^{-3}	1.139	0.998
Platelet count	Cervical intraepithelial neoplasia (SR)	6.24×10^{-4}	59.720	1.000
Platelet count	Alzheimer's	2.23×10^{-3}	1.108	0.998
Platelet count	Hayfever	2.10×10^{-3}	1.879	0.999
Platelet count	Psoriasis	1.28×10^{-3}	7.416	1.000
Platelet count	Ulcerative colitis	1.46×10^{-3}	0.668	0.998
Platelet count	Allergy	3.37×10^{-2}	13.029	0.998
Platelet count	Infection	2.13×10^{-3}	0.438	0.996

Table S17: Power calculations for Mendelian Randomisation analyses for causal effect of blood cell counts on counts on infection and inflammation-related disease

Exposure	Outcome	Power for Full cohort
RBC count	Influenza (ICD)	2.50%
RBC count	*Legionella spp*. (ICD)	2.50%
RBC count	*Neisseria meningitidis* (ICD)	2.50%
RBC count	Papillomavirus (ICD)	2.50%
RBC count	Cervical intraepithelial neoplasia (SR)	2.50%
RBC count	Alzheimer's	2.50%
RBC count	Hayfever	2.50%
RBC count	Psoriasis	2.50%
RBC count	Ulcerative colitis	2.50%
RBC count	Allergy	3.00%
RBC count	Infection	2.50%
Basophil count	Influenza (ICD)	2.50%
Basophil count	*Legionella spp*. (ICD)	2.50%
Basophil count	*Neisseria meningitidis* (ICD)	2.50%
Basophil count	Papillomavirus (ICD)	2.50%
Basophil count	Cervical intraepithelial neoplasia (SR)	2.50%
Basophil count	Alzheimer's	2.50%
Basophil count	Hayfever	2.50%
Basophil count	Psoriasis	2.50%
Basophil count	Ulcerative colitis	2.50%
Basophil count	Allergy	2.80%
Basophil count	Infection	2.60%
Neutrophil count	Influenza (ICD)	2.50%
Neutrophil count	*Legionella spp*. (ICD)	2.50%
Neutrophil count	*Neisseria meningitidis* (ICD)	2.50%
Neutrophil count	Papillomavirus (ICD)	2.50%
Neutrophil count	Cervical intraepithelial neoplasia (SR)	2.50%
Neutrophil count	Alzheimer's	2.50%
Neutrophil count	Hayfever	2.50%
Neutrophil count	Psoriasis	2.50%
Neutrophil count	Ulcerative colitis	2.50%
Neutrophil count	Allergy	2.70%
Neutrophil count	Infection	2.60%
Monocyte count	Influenza (ICD)	2.50%
Monocyte count	*Legionella spp*. (ICD)	2.50%
Monocyte count	*Neisseria meningitidis* (ICD)	2.50%
Monocyte count	Papillomavirus (ICD)	2.50%
Monocyte count	Cervical intraepithelial neoplasia (SR)	2.50%
Monocyte count	Alzheimer's	2.50%
Monocyte count	Hayfever	2.50%
Monocyte count	Psoriasis	2.50%
Monocyte count	Ulcerative colitis	2.50%
Monocyte count	Allergy	2.90%
Monocyte count	Infection	2.60%

Eosinophil count	Influenza (ICD)	2.50%
Eosinophil count	*Legionella spp*. (ICD)	2.50%
Eosinophil count	*Neisseria meningitidis* (ICD)	2.50%
Eosinophil count	Papillomavirus (ICD)	2.50%
Eosinophil count	Cervical intraepithelial neoplasia (SR)	2.50%
Eosinophil count	Alzheimer's	2.50%
Eosinophil count	Hayfever	2.50%
Eosinophil count	Psoriasis	2.50%
Eosinophil count	Ulcerative colitis	2.50%
Eosinophil count	Allergy	2.70%
Eosinophil count	Infection	2.50%
Lymphocyte count	Influenza (ICD)	2.50%
Lymphocyte count	*Legionella spp*. (ICD)	2.50%
Lymphocyte count	*Neisseria meningitidis* (ICD)	2.50%
Lymphocyte count	Papillomavirus (ICD)	2.50%
Lymphocyte count	Cervical intraepithelial neoplasia (SR)	2.50%
Lymphocyte count	Alzheimer's	2.50%
Lymphocyte count	Hayfever	2.50%
Lymphocyte count	Psoriasis	2.50%
Lymphocyte count	Ulcerative colitis	2.50%
Lymphocyte count	Allergy	3.00%
Lymphocyte count	Infection	2.50%
Platelet count	Influenza (ICD)	2.50%
Platelet count	*Legionella spp*. (ICD)	2.50%
Platelet count	*Neisseria meningitidis* (ICD)	2.50%
Platelet count	Papillomavirus (ICD)	2.50%
Platelet count	Cervical intraepithelial neoplasia (SR)	2.50%
Platelet count	Alzheimer's	2.50%
Platelet count	Hayfever	2.50%
Platelet count	Psoriasis	2.50%
Platelet count	Ulcerative colitis	2.50%
Platelet count	Allergy	2.80%
Platelet count	Infection	2.50%

Table S18: Variance explained and F statistics for rs28362491 for blood cell counts from the full UK Biobank cohort.

Phenotype	Variance	F statistic
RBC count	8.20×10^{-5}	38.739
Basophil count	7.64×10^{-5}	36.011
Neutrophil count	2.84×10^{-5}	13.399
Monocyte count	1.32×10^{-4}	62.484
Eosinophil count	2.32×10^{-5}	10.960
Lymphocyte count	2.18×10^{-4}	103.028
Leukocyte count	4.94×10^{-9}	0.002
Platelet count	2.20×10^{-5}	10.414

References

1. Brenner, N., Mentzer, A.J., Butt, J., Michel, A., Prager, K., Brozy, J., Weißbrich, B., Aiello, A.E., Meier, H.C.S., Breuer, J., et al. (2018). Validation of Multiplex Serology detecting human herpesviruses 1-5. PLoS One 13, e0209379-e0209379.
2. Brenner, N., Mentzer, A.J., Butt, J., Braband, K.L., Michel, A., Jeffery, K., Klenerman, P., Gärtner, B., Schnitzler, P., Hill, A., et al. (2019). Validation of Multiplex Serology for human hepatitis viruses B and C, human T-lymphotropic virus 1 and Toxoplasma gondii. PLoS One 14, e0210407-e0210407.
3. Dondog, B., Schnitzler, P., Michael, K.M., Clifford, G., Franceschi, S., Pawlita, M., and Waterboer, T. (2015). Hepatitis C Virus Seroprevalence in Mongolian Women Assessed by a Novel Multiplex Antibody Detection Assay. Cancer Epidemiology Biomarkers & Prevention 24, 1360-1365.
4. Kranz, L.M., Gärtner, B., Michel, A., Pawlita, M., Waterboer, T., and Brenner, N. (2019). Development and validation of HIV-1 Multiplex Serology. Journal of Immunological Methods 466, 47-51.
5. Gossai, A., Waterboer, T., Nelson, H.H., Doherty, J.A., Michel, A., Willhauck-Fleckenstein, M., Farzan, S.F., Christensen, B.C., Hoen, A.G., Perry, A.E., et al. (2016). Prospective Study of Human Polyomaviruses and Risk of Cutaneous Squamous Cell Carcinoma in the United States. Cancer Epidemiology Biomarkers & Prevention 25, 736-744.
6. Kjærheim, K., Røe, O.D., Waterboer, T., Sehr, P., Rizk, R., Dai, H.Y., Sandeck, H., Larsson, E., Andersen, A., Boffetta, P., et al. (2007). Absence of SV40 antibodies or DNA fragments in prediagnostic mesothelioma serum samples. International Journal of Cancer 120, 2459-2465.
7. Robles, C., Casabonne, D., Benavente, Y., Costas, L., Gonzalez-Barca, E., Aymerich, M., Campo, E., Tardon, A., Jiménez-Moleón, J.J., Castaño-Vinyals, G., et al. (2015). Seroreactivity against Merkel cell polyomavirus and other polyomaviruses in chronic lymphocytic leukaemia, the MCC-Spain study. Journal of General Virology 96, 2286-2292.
8. Sehr, P., Müller, M., Höpfl, R., Widschwendter, A., and Pawlita, M. (2002). HPV antibody detection by ELISA with capsid protein L1 fused to glutathione S-transferase. Journal of Virological Methods 106, 61-70.
9. Sehr, P., Zumbach, K., and Pawlita, M. (2001). A generic capture ELISA for recombinant proteins fused to glutathione S-transferase: validation for HPV serology. Journal of Immunological Methods 253, 153-162.
10. Hulstein, S.H., Matser, A., Alberts, C.J., Snijder, M.B., Willhauck-Fleckenstein, M., Hufnagel, K., Prins, M., de Vries, H.J.C., Schim van der Loeff, M.F., and Waterboer, T. (2018). Differences in Chlamydia trachomatis seroprevalence between ethnic groups cannot be fully explained by socioeconomic status, sexual healthcare seeking behavior or sexual risk behavior: a cross-sectional analysis in the HEalthy LIfe in an Urban Setting (HELIUS) study. BMC Infectious Diseases 18, 612-612.
11. Trabert, B., Waterboer, T., Idahl, A., Brenner, N., Brinton, L.A., Butt, J., Coburn, S.B., Hartge, P., Hufnagel, K., Inturrisi, F., et al. (2018). Antibodies Against Chlamydia trachomatis and Ovarian Cancer Risk in Two Independent Populations. JNCI: Journal of the National Cancer Institute 111, 129-136.
12. Michel, A., Waterboer, T., Kist, M., and Pawlita, M. (2009). Helicobacter pylori Multiplex Serology. Helicobacter 14, 525-535.
13. Waage, J., Standl, M., Curtin, J.A., Jessen, L.E., Thorsen, J., Tian, C., Schoettler, N., andMe Research, T., collaborators, A., Flores, C., et al. (2018). Genome-wide association and HLA fine-mapping studies identify risk loci and genetic pathways underlying allergic rhinitis. Nat Genet 50, 1072-1080.
14. Johansson, A., Rask-Andersen, M., Karlsson, T., and Ek, W.E. (2019). Genome-wide association analysis of 350 000 Caucasians from the UK Biobank identifies novel loci for asthma, hay fever and eczema. Hum Mol Genet 28, 4022-4041.
15. Tian, C., Hromatka, B.S., Kiefer, A.K., Eriksson, N., Noble, S.M., Tung, J.Y., and Hinds, D.A. (2017). Genome-wide association and HLA region fine-mapping studies identify susceptibility loci for multiple common infections. Nat Commun 8, 599-599.
16. Bajwa, E.K., Cremer, P.C., Gong, M.N., Zhai, R., Su, L., Thompson, B.T., and Christiani, D.C. (2011). An NFKB1 promoter insertion/deletion polymorphism influences risk and outcome in acute respiratory distress syndrome among Caucasians. PLoS One 6, e19469-e19469.

17. Ellinghaus, D., Jostins, L., Spain, S.L., Cortes, A., Bethune, J., Han, B., Park, Y.R., Raychaudhuri, S., Pouget, J.G., Hubenthal, M., et al. (2016). Analysis of five chronic inflammatory diseases identifies 27 new associations and highlights disease-specific patterns at shared loci. Nat Genet 48, 510-518.
18. Yenmis, G., Oner, T., Cam, C., Koc, A., Kucuk, O.S., Yakicier, M.C., Dizman, D., and Kanigur Sultuybek, G. (2015). Association of NFKB1 and NFKBIA polymorphisms in relation to susceptibility of Behcet's disease. Scand J Immunol 81, 81-86.
19. Seidi, A., Mirzaahmadi, S., Mahmoodi, K., and Soleiman-Soltanpour, M. (2018). The association between NFKB1 -94ATTG ins/del and NFKB1A 826C/T genetic variations and coronary artery disease risk. Mol Biol Res Commun 7, 17-24.
20. Mishra, A., Srivastava, A., Mittal, T., Garg, N., and Mittal, B. (2013). Role of inflammatory gene polymorphisms in left ventricular dysfunction (LVD) susceptibility in coronary artery disease (CAD) patients. Cytokine 61, 856-861.
21. Lai, H.-M., Li, X.-M., Yang, Y.-N., Ma, Y.-T., Xu, R., Pan, S., Zhai, H., Liu, F., Chen, B.-D., and Zhao, Q. (2015). Genetic Variation in NFKB1 and NFKBIA and Susceptibility to Coronary Artery Disease in a Chinese Uygur Population. PLOS ONE 10, e0129144-e0129144.
22. Adamzik, M., Frey, U.H., Rieman, K., Sixt, S., Beiderlinden, M., Siffert, W., and Peters, J. (2007). Insertion/deletion polymorphism in the promoter of NFKB1 influences severity but not mortality of acute respiratory distress syndrome. Intensive Care Med 33, 1199-1203.
23. Dudding, T., Haworth, S., Lind, P.A., Sathirapongsasuti, J.F., andMe Research, T., Tung, J.Y., Mitchell, R., Colodro-Conde, L., Medland, S.E., Gordon, S., et al. (2019). Genome wide analysis for mouth ulcers identifies associations at immune regulatory loci. Nat Commun 10, 1052-1052.
24. Boccardi, V., Rizzo, M.R., Marfella, R., Papa, M., Esposito, A., Portoghese, M., Paolisso, G., and Barbieri, M. (2011). -94 ins/del ATTG NFKB1 gene variant is associated with lower susceptibility to myocardial infarction. Nutr Metab Cardiovasc Dis 21, 679-684.
25. Kawashima, M., Hitomi, Y., Aiba, Y., Nishida, N., Kojima, K., Kawai, Y., Nakamura, H., Tanaka, A., Zeniya, M., Hashimoto, E., et al. (2017). Genome-wide association studies identify PRKCB as a novel genetic susceptibility locus for primary biliary cholangitis in the Japanese population. Hum Mol Genet 26, 650-659.
26. Gonzalez-Serna, D., Lopez-Isac, E., Yilmaz, N., Gharibdoost, F., Jamshidi, A., Kavosi, H., Poursani, S., Farsad, F., Direskeneli, H., Saruhan-Direskeneli, G., et al. (2019). Analysis of the genetic component of systemic sclerosis in Iranian and Turkish populations through a genome-wide association study. Rheumatology (Oxford) 58, 289-298.
27. Liou, Y.J., Wang, H.H., Lee, M.T., Wang, S.C., Chiang, H.L., Chen, C.C., Lin, C.H., Chung, M.S., Kuo, C.C., Liao, D.L., et al. (2012). Genome-wide association study of treatment refractory schizophrenia in Han Chinese. PLoS One 7, e33598-e33598.
28. Jostins, L., Ripke, S., Weersma, R.K., Duerr, R.H., McGovern, D.P., Hui, K.Y., Lee, J.C., Schumm, L.P., Sharma, Y., Anderson, C.A., et al. (2012). Host-microbe interactions have shaped the genetic architecture of inflammatory bowel disease. Nature 491, 119-124.
29. Karban, A.S., Okazaki, T., Panhuysen, C.I., Gallegos, T., Potter, J.J., Bailey-Wilson, J.E., Silverberg, M.S., Duerr, R.H., Cho, J.H., Gregersen, P.K., et al. (2004). Functional annotation of a novel NFKB1 promoter polymorphism that increases risk for ulcerative colitis. Hum Mol Genet 13, 35-45.
30. Borm, M.E., van Bodegraven, A.A., Mulder, C.J., Kraal, G., and Bouma, G. (2005). A NFKB1 promoter polymorphism is involved in susceptibility to ulcerative colitis. Int J Immunogenet 32, 401-405.
31. Wang, D., Xie, T., Xu, J., Wang, H., Zeng, W., Rao, S., Zhou, K., Pei, F., and Zhou, Z. (2016). Genetic association between NFKB1 -94 ins/del ATTG Promoter Polymorphism and cancer risk: a meta-analysis of 42 case-control studies. Sci Rep 6, 30220-30220.
32. Kanai, M., Akiyama, M., Takahashi, A., Matoba, N., Momozawa, Y., Ikeda, M., Iwata, N., Ikegawa, S., Hirata, M., Matsuda, K., et al. (2018). Genetic analysis of quantitative traits in the Japanese population links cell types to complex human diseases. Nat Genet 50, 390-400.
33. Loh, P.R., Tucker, G., Bulik-Sullivan, B.K., Vilhjalmsson, B.J., Finucane, H.K., Salem, R.M., Chasman, D.I., Ridker, P.M., Neale, B.M., Berger, B., et al. (2015). Efficient Bayesian mixed-model analysis increases association power in large cohorts. Nat Genet 47, 284-290.

34. Jonsson, S., Sveinbjornsson, G., de Lapuente Portilla, A.L., Swaminathan, B., Plomp, R., Dekkers, G., Ajore, R., Ali, M., Bentlage, A.E.H., Elmer, E., et al. (2017). Identification of sequence variants influencing immunoglobulin levels. Nat Genet 49, 1182-1191.
