# Supplementary material for: A common NFKB1 variant detected through antibody analysis in UK Biobank predicts risk of infection and allergy
Source: Am J Hum Genet. 2024 Jan 16;111(2):295–308. doi: 10.1016/j.ajhg.2023.12.013 (PMC10870136; doi:10.1016/j.ajhg.2023.12.013)
Supplement: Document S2. Article plus supplemental information [file mmc3.pdf]

A common *NFKB1* variant detected through antibody analysis in UK Biobank predicts risk of infection and allergy

Authors

Amanda Y. Chong, Nicole Brenner, Andres Jimenez-Kaufmann, ..., Tim Waterboer, Adrian V.S. Hill, Alexander J. Mentzer

Correspondence

amanda.chong@well.ox.ac.uk (A.Y.C.), alexander.mentzer@ndm.ox.ac.uk (A.J.M.)

Infectious agents contribute significantly to the global burden of diseases, through both acute infection and their chronic sequelae. We identified an insertion-deletion variant thought to affect *NFKB1* expression as a causal variant—central to serological responses to diverse infectious agents, risk of infection, immune cell survival, antibody production, and inflammation.

Chong et al., 2024, The American Journal of Human Genetics *111*, 295–308
February 1, 2024 © 2023 The Authors.
https://doi.org/10.1016/j.ajhg.2023.12.013

A common *NFKB1* variant detected through antibody analysis in UK Biobank predicts risk of infection and allergy

Amanda Y. Chong,[1,*] Nicole Brenner,[2] Andres Jimenez-Kaufmann,[3] Adrian Cortes,[4] Michael Hill,[5] Thomas J. Littlejohns,[6] James J. Gilchrist,[1,7] Benjamin P. Fairfax,[8] Julian C. Knight,[1,9] Flavia Hodel,[10,11] Jacques Fellay,[10,11,12] Gil McVean,[4] Andres Moreno-Estrada,[3] Tim Waterboer,[2] Adrian V.S. Hill,[1,13] and Alexander J. Mentzer[1,4,14,*]

Summary

Infectious agents contribute significantly to the global burden of diseases through both acute infection and their chronic sequelae. We leveraged the UK Biobank to identify genetic loci that influence humoral immune response to multiple infections. From 45 genome-wide association studies in 9,611 participants from UK Biobank, we identified *NFKB1* as a locus associated with quantitative antibody responses to multiple pathogens, including those from the herpes, retro-, and polyoma-virus families. An insertion-deletion variant thought to affect *NFKB1* expression (rs28362491), was mapped as the likely causal variant and could play a key role in regulation of the immune response. Using 121 infection- and inflammation-related traits in 487,297 UK Biobank participants, we show that the deletion allele was associated with an increased risk of infection from diverse pathogens but had a protective effect against allergic disease. We propose that altered expression of *NFKB1*, as a result of the deletion, modulates hematopoietic pathways and likely impacts cell survival, antibody production, and inflammation. Taken together, we show that disruptions to the tightly regulated immune processes may tip the balance between exacerbated immune responses and allergy, or increased risk of infection and impaired resolution of inflammation.

Introduction

There is significant evidence that human genetic variation influences susceptibility to acute disease caused by infectious pathogens. Case-control genome-wide association studies (GWASs) of a variety of pathogens and infections, such as human immunodeficiency virus, hepatitis B and C viruses, leprosy, typhoidal and non-typhoidal *Salmonella*, meningococcal disease, and more recently SARS-CoV-2, have identified a range of variants spanning pathogen recognition, immune activation, and immunologic memory robustly associated with differential susceptibility.[1–10] Furthermore, using large biobanks of human genetic data linked with participant self-reporting, studies such as 23andMe are replicating and building on these findings from case-control analyses of infection susceptibility.[11] Together, these studies are helping improve our understanding of how genetic variants influence the delicate balance of immunity and risk of invasive infection that give rise to diseases such as meningitis, mononucleosis, or plantar warts and more autoimmune reactions to infections such as rheumatic heart disease. Despite these advances, there remain challenges to using both study approaches. Firstly, achieving sufficient power to discover genetic signals in case-control studies is limited by challenges in recruiting large numbers of cases, and both study approaches may be affected by pathogen heterogeneity that may have an impact on signatures of genetic association, while the self-report approach will be influenced by limitations in accuracy and recall of self-reporting.

Antibodies are a critical component of response to infection and represent a marker of prior exposure or chronic carriage with agents capable of latent or chronic infection states. Now that methods are available to accurately measure multiple antibodies simultaneously in large numbers of individuals, these represent stable biological markers that can be used to understand disease risk. Furthermore, there is evidence from vaccine studies that the magnitude of antibody response and levels of circulating antibody may reflect the likelihood of protection against either primary or subsequent disease, although this may depend on the antigen to which the antibody is directed, and the functional impact of antibody binding and activity.[12]

[1]The Wellcome Centre for Human Genetics, University of Oxford, Oxford, UK; [2]Division of Infections and Cancer Epidemiology, German Cancer Research Center (DKFZ), Heidelberg, Germany; [3]Advanced Genomics Unit, National Laboratory of Genomics for Biodiversity (LANGEBIO), CINVESTAV, Irapuato, Mexico; [4]Big Data Institute, Li Ka Shing Centre for Health Information and Discovery, University of Oxford, Oxford, UK; [5]MRC-Population Health Research Unit, University of Oxford, Oxford, UK; [6]Nuffield Department of Population Health, University of Oxford, Oxford, UK; [7]Department of Paediatrics, University of Oxford, Oxford, UK; [8]Department of Oncology, University of Oxford, Oxford, UK; [9]NIHR Oxford Biomedical Research Centre, Oxford University Hospitals NHS Foundation Trust, Oxford, UK; [10]Global Health Institute, School of Life Sciences, EPFL, Lausanne, Switzerland; [11]Swiss Institute of Bioinformatics, Lausanne, Switzerland; [12]Precision Medicine Unit, Lausanne University Hospital and University of Lausanne, Lausanne, Switzerland; [13]The Jenner Institute, University of Oxford, Oxford, UK
[14]Senior author
*Correspondence: amanda.chong@well.ox.ac.uk (A.Y.C.), alexander.mentzer@ndm.ox.ac.uk (A.J.M.)
https://doi.org/10.1016/j.ajhg.2023.12.013.
© 2023 The Authors. This is an open access article under the CC BY license (http://creativecommons.org/licenses/by/4.0/).

Human genetic variation is recognized to significantly influence total antibody levels.[13] Genetic analyses of antibody response levels against vaccine preventable infections, such as hepatitis B virus (HBV), have demonstrated a strong influence of genetic loci, including the major histocompatibility complex (MHC) in predicting antibody magnitude overlapping with GWAS of disease susceptibility.[14] The MHC has also been implicated in varied antibody responses to infections, including JC virus and influenza.[15–19] Furthermore, primary immunodeficiencies, such as common variable immunodeficiency (CVID) and X-linked agammaglobulinemia, are well recognized disorders that increase the risk of multiple infections owing to impaired humoral function and antibody deficiencies.[20–22]

The availability of antibody data against 45 antigens representing 20 infectious agents in combination with genome-wide genotyping data in a subset of UK Biobank participants now offers the opportunity to investigate whether there are common variants that influence both response to antibodies and subsequent susceptibility to both acute infectious disease and their chronic sequelae. While previous studies have identified genetic associations with single antigens or pathogens using this UK Biobank dataset,[17,23] we hypothesize that there are genetic variants and loci that have a common effect on antibody responses to multiple infections through effects on central immunological pathways. To identify genetic loci that influence antibody titers against multiple infections, we performed GWASs for a magnitude of antibody responses to each antigen and identified *NFKB1* as a distinct locus of interest in contrast to the other, more familiar loci that were identified, including immunoglobulin genes, the MHC, and *FUT2*. Given the role of *NFKB1* in inflammation and the induction and regulation of immune responses to infection, we further investigated the association between *NFKB1* variation and infectious and inflammatory diseases using data from 487,297 individuals. Finally, to better understand the impact of variation in this locus on underlying immune mechanisms, we explored the effect of this variant on blood cell traits and cellular expression data both within UK Biobank and in dedicated expression quantitative trait datasets.

Subjects and methods

Serology and GWAS

We used serological measurements of infectious agents released as part of the UK Biobank Data Showcase[24,25] to investigate genetic loci associated with susceptibility to infectious disease. This data consist of measured antibody responses against 45 antigens from 20 viral, bacterial, and protozoan pathogens present in the UK (Tables S1–S3) from a randomly selected subset (n = 9,695) of the UK Biobank cohort. Viral pathogens included on the panel included herpes simplex virus types 1 and 2 (HSV-1 and -2), varicella-zoster virus (VZV), Epstein-Barr virus (EBV), human cytomegalovirus (CMV), human herpesvirus 6 and 7 (HHV-6 and -7), Kaposi's sarcoma-associated herpesvirus (KSHV), HBV, hepatitis C virus (HCV), human T-lymphotropic virus 1 (HTLV-1), human immunodeficiency virus 1 (HIV-1), BK and JC polyomaviruses (BKV and JCV), Merkel cell polyomavirus (MCV), and human papillomavirus 16 and 18 (HPV-16 and -18). Other pathogens assayed were the bacterial pathogens *Chlamydia trachomatis* and *Helicobacter pylori*, as well as protozoan parasite *Toxoplasma gondii*. Target antigens were selected based on known biological function and validation assays (Table S1). Measurements from samples collected at the initial assessment visit were used for analysis. Mean age of participants was 56.55 years at time of sample collection.

For all association tests, we used the genotyped and imputed autosomal variant datasets available for the UK Biobank cohort.[26] This consists of 805,426 high-quality genotyped variants, generated from the UK BiLEVE and UK Biobank Axiom arrays, and 93,095,623 imputed autosomal variants were imputed via the Haplotype Reference Consortium (HRC), UK10K, and 1000 Genomes phase 3 reference panels.[27–30] Quality control filters for poorly performing variants and samples prior to imputation are described in Bycroft et al.[26] UK Biobank has obtained ethics approval from the North West Multi-centre Research Ethics Committee (approval number: 11/NW/0382) and had obtained informed consent from all participants.

For our analysis we had 9,611 unrelated individuals that had genetic and serological data available. Antibody responses across all participants regardless of serostatus were normalized using a rank-based inverse normal transformation (INT). All estimated betas are reported relative to standard deviations of the normalized distribution. We measured the Spearman's rank correlation between traits to determine whether there was any evidence of correlation between antibody traits (Figure S1). Correlation measurements were repeated after regressing out the effects of age, sex, and lead genetic variants as identified from GWAS analyses (Figure S2). Regression analyses were carried out using fitted generalized linear models as implemented in the python statsmodels package and normalized MFI values.

Association analyses using imputed genotype data and both the untransformed and normalized data from quantitative serology traits were carried out using linear mixed models as implemented in BOLT-LMM (minor allele frequency [MAF] >0.01, imputation infoscore >0.3;[31]) including age at recruitment and genetic sex as covariates. All participants were included irrespective of ethnic origin as the use of linear mixed models allows us to account for population substructure through the incorporation of a genetic relatedness matrix (GRM) as a random effect covariate. Reported betas and standard errors are based on normalized distributions and reflect magnitude of response. To identify the genomic regions and candidate genes associated with antibody response to each of the tested antigens, we first identified SNPs associated with tested antigens based on genome-wide significance ($p < 5 \times 10^{-8}$) and suggestive significance ($p < 1 \times 10^{-5}$). Starting with the most significant SNPs (peak SNP), we defined associated regions for each antigen based on calculated linkage disequilibrium (LD) from best-guess genotypes ($R^2 \geq 0.2$, calculated with PLINK; Chang et al.[32]) between the peak SNP and other SNPs reaching suggestive significance for the dataset. This was repeated for each SNP that was not linked with a previous peak SNP. We then merged overlapping regions across all traits to define independent regions of association for further analysis.

Fine mapping of GWAS signals

For regions containing SNPs with an association p value less than the Bonferroni corrected p value ($p < 1.1 \times 10^{-9}$), we then performed conditional analyses to determine if there were multiple independent signals present within the region. Analyses were carried out in GCTA using the –mlma option and adjusting for allelic dosage of the top SNP for each associated trait.[33] These gene dosages were also used to test for interdependence between these associated regions and other regions of interest.

FINEMAP was used (default settings, $R2 = 0.8$; Benner et al.[34]) to identify potential causal groups (Bayes factor ≥ 2) of SNPs within each of these regions based on summary statistics for each of the associated traits. These were then compared across antigens and merged to define haplotypes of SNPs based on shared SNPs and direction of effect. Causal groups sharing SNPs were only merged if the direction of effect across all antigens were in a consistent direction relative to the minor allele.

Potential causal SNPs were identified from haplotypes shared across multiple antigens. To do this, we first checked the location of SNPs relative to the Ensembl GRCh37.p13 gene annotations to identify the nearest genes. The predicted effects of each variant were then retrieved using the Ensembl Variant Effect Predictor.[33]

Multivariate GWAS

Following fine mapping of association signals in the *NFKB1* region, we carried out a multivariate GWAS using the seven phenotypes with a predicted causal SNP in *NFKB1*. The phenotypes included were EBV (EAD), HHV-6 (IE1A), HHV-7 (U14), HIV (env), HPV-18 (L1), KSHV (K8.1), and *T. gondii* (sag1). A multivariate GWAS was carried out using SNPTEST (v2.5.6;[35]), and the INT normalized phenotypes. Age at recruitment, sex, and the top four principal components were included as covariates, as SNPTEST does not fit a GRM as part of the association model. Principal components were calculated from LD-pruned genotype data using PLINK (v1.90b3).

Cohorte Lausannoise replication study and meta-analysis

The Cohorte Lausannoise (CoLaus/PsyCoLaus) study includes 6,188 individuals of European ancestry living in Lausanne, Switzerland who were randomly selected from the general population. Recruitment and sampling protocols are described by Firmann et al.[36] The institutional ethics committee of the University of Lausanne, which afterward became the Ethics Commission of Canton Vaud (www.cer-vd.ch), approved the baseline CoLaus study (ref. 16/03, decisions of January 13 and February 10, 2003), and written consent was obtained from all participants.

Genotyping data for 5,399 individuals were obtained using the Affymetrix Axiom Biobank array. Following quality control steps to remove poorly performing variants and samples, imputed genotypes were generated with the Sanger Imputation Service.[30] Genotypes were phased using EAGLE2 (v2.0.5; Loh et al.[29]) and the HRC panel,[30] and imputation was carried out using the HRC reference panel, 1000 Genomes Phase 3 data, and the UK10K reference panel.[27,28] Serology phenotypes were generated using a similar Multiplex Serology Panel to the UK Biobank data as described above (Table S3).[16,25]

Genome-wide association analyses were carried out using 4,216 individuals with imputed genotype data and serology data and linear mixed models as implemented in GCTA (1.91.3beta) and thresholded high-quality imputed genotype data (imputation infoscore >0.8, MAF >0.01, $P_{\mathrm{HWE}} < 10 \times 10^{-7}$). Age, sex, and the top three principal components were included as covariates. For antigens with a signal at *NFKB1* and that were also available from the CoLaus/PsyCoLaus cohort, we performed GWASs of antibody levels that were common across the two cohorts and extracted the set of intersecting SNPs for meta-analysis using Metasoft (v2.0.1; Han and Eskin[37]).

Infection and inflammation associations in UK Biobank

We used self-reported non-cancer illness (Field ID: 20002) and prevalent and incident hospital inpatient International Classification of Disease version 10 (ICD-10) codes (Field ID: 41270) to define cases and controls for further infection- and inflammation-related phenotypes within UK Biobank. For self-reported conditions, cases and controls were drawn from participants who reported the presence or absence of non-cancer-related conditions and had genetic data available, excluding those who did not respond to the question. Controls for infection-related traits derived from self-reported data were defined as individuals who had reported no major infections for infection-related phenotypes with genetic data available. Controls for other immune-related traits were drawn from all unaffected individuals with genetic data.

Similarly, potential cases and controls based on ICD-10 data were drawn from those individuals with hospital inpatient diagnoses and genetic data available. Case definitions are outlined in Tables S4 and S5. Controls for ICD-10-derived phenotypes were drawn from individuals with hospital inpatient and genetic data who were not affected by the trait of interest. In the case of infection-related traits, the selection was further restricted to those who had not reported a major infection or had no ICD codes relating to infection (e.g., Chapter I [A00-B99], Chapter X: Diseases of the respiratory system: Acute upper respiratory infections [J00-J06], Influenza and pneumonia [J09-J18], other acute lower respiratory infections [J20-J22]).

Where equivalent phenotypes were present in both self-reported and ICD-10 data, a joint phenotype was defined using all possible cases and controls. Similarly, summary phenotypes for allergy, infection, and inflammation were defined using all available cases and controls. Due to the small numbers of cases across many phenotypes, we used only those with more than 50 cases.

Association tests for these traits were carried out using linear mixed models as implemented in SAIGE (v 0.36.3.2; Zhou et al.[38]), also including age at recruitment and genetic sex as covariates, except for traits where only one sex was present in the cases, in which case sex was not included, but controls were drawn only from individuals of that sex (Tables S4 and S5 for more information). Due to relatively small numbers of individuals for which cause of death was available (approximately 25,000), we used logistic regression to assess the relationship between rs28362491:delATTG and the contribution of infection or inflammatory conditions to cause of death. Similar to the GWAS analysis described above, we used imputed dosages of rs28362491:delATTG with age and sex as covariates. We also restricted analysis to individuals in the White British subset as defined by UK Biobank.

Association with blood cell counts

Association tests for blood cell counts were carried out on the full UK Biobank cohort using transformed cell-count data and imputed genotypes. Traits included red blood cell (RBC) count, white blood cell (WBC) count, platelet, basophil, eosinophil, lymphocyte, monocyte, and neutrophil counts. Values were

normalized using a rank-based inverse normal transformation prior to analysis. Association tests were carried out using BOLT-LMM, including age at recruitment and genetic sex as covariates.

Expression quantitative trait locus analysis

To assess the regulatory function of rs28362491, we used the tightly linked 5′UTR SNP rs72696119 ($r^2 = 0.999$, D′ = 0.999). We correlated rs72696119 genotype with *NFKB1* mRNA expression in naive and stimulated primary immune cell subsets from healthy European adults, using data from expression quantitative trait locus (eQTL) studies. $CD56^+CD3^-$ NK cells, $CD19^+$ B cells, $CD14^+$ monocytes, and $CD16^+$ neutrophils were separated, genotyped, and gene expression quantified as previously described.[39–42] Following quality control and normalization of gene-expression data, we correlated rs72696119 genotype with *NFKB1* RNA expression in each cell type: B cells (n = 279), NK cells (n = 245), neutrophils (n = 101), naive monocytes (n = 414), and stimulated monocytes (lipopolysaccharide [LPS] 2 h, n = 261; LPS 24 h, n = 322; interferon gamma [IFNγ] 24 h, n = 367). *NFKB1* expression was correlated with genotype by linear regression and analysis of variance (ANOVA), including the first 25 principal components of gene-expression data in each cell type/condition to account for confounding variation. p- values are calculated with F-tests (1 degree of freedom). Statistical analysis was performed in R. We used publicly available eQTL data from the DICE database for $CD4^+$ and $CD8^+$ T cells to examine association of rs72696119 with *NFKB1* expression in naive T cells (Schmiedel et al.[43]; https://dice-database.org/).

Colocalization of eQTL and GWAS signals

Colocalization testing between eQTL signals and antibody and blood cell GWAS association signals was carried out using coloc (v5.0.1; Wallace[44]) with susieR (v0.11.92; Wang et al. and Zou et al.[45,46]) and the intersection of variants from all GWAS phenotypes and the eQTL traits from the region immediately upstream of and across NFKB1. LD was calculated independently for each dataset using PLINK. Colocalization was tested between pairs of antibody traits: between HHV6 (IE1A) and blood cell traits, between HHV6 (IE1A) and eQTL datasets, and between related blood cell traits and eQTL datasets (e.g., monocyte count and unstimulated and stimulated monocyte eQTL datasets) (Table S13).

Mendelian randomization

The causal effect of blood cell counts on antibody response and risk of disease were tested using one- and two-sample Mendelian randomization (MR) by using rs28362491 as the genetic instrumental variable. We used normalized cell counts for RBCs, basophils, eosinophils, neutrophils, monocytes, lymphocytes, and platelets. Antibody response measures and health-record-derived traits were restricted to those with a significant association at rs28362491 (antibody response: EBV [EAD and ZEBRA], HHV-6 [IE1A], HHV-7 [U14], HPV-18 [L1], HTLV-1 [env], KSHV [K8.1], and *T. gondii* [sag1]; health records: hay fever, influenza [ICD], *Legionella* spp. [ICD], *Neisseria meningitidis* [ICD], papillomavirus [ICD], psoriasis, ulcerative colitis, Alzheimer's disease, cervical intraepithelial neoplasia [SR], allergy, and infection). Bias as a result of overlapping samples between datasets for two-sample analyses are expected to be negligible given the small size of the antibody dataset in comparison with the full UK Biobank cohort.[47]

We used the inverse-variance weighted (IVW) method as implemented in the R package MendelianRandomization[48,49] for two-sample MR and multiplicative structural mean model (MSMM; binary outcome) from R package OnesampleMR (https://github.com/remlapmot/OneSampleMR) for one-sample analyses. Power calculations for pairs of traits were carried out using an online power calculator (https://sb452.shinyapps.io/power/). The variance in the exposure explained by rs28362491 (R^2) and F statistics for instrument strength was also calculated. An F statistic >10 was taken to be indicative of a low risk of weak instrument bias in MR analyses.

Results

We have previously described the use of a Multiplex Serology panel to measure immunoglobulin G (IgG) antibody responses against 45 antigens from 20 infectious agents implicated in the pathogenesis of chronic disease, applied to 9,695 individuals in UK Biobank.[24] We observed that antibody responses to antigens from the same pathogen were more strongly correlated with each other (Spearman's rho 0.10–0.86; Figure S1) than with those of other pathogens (Spearman's rho −0.05-0.75) and that all antibody responses were generally weakly correlated with each other with the exception of KSHV (K8.1), HIV (env), HTLV-1 (env), and HPV-18 (L1), which were all strongly correlated (Spearman's rho 0.70–0.75). However, seroprevalence estimates for these pathogens in UK Biobank are very low (0.2%–8.1%; Mentzer et al.[24]). Since cross-reactivity between antigens was found to be negligible for all antigens included in the current panel, except for HTLV-1 gag,[24] it is likely that shared genetic variants and environmental factors influence these correlated responses. Using the 9,611 individuals with both serology data and imputed genetic data, we performed GWASs of quantitative antibody responses to 45 antigens to identify these common genetic loci that influence response to multiple infections.

We identified 27 genome-wide significant ($p < 5 \times 10^{-8}$) loci associated with magnitude of antibody responses to any antigen (Figure 1; Table S6). We observed four genomic regions, localizing to *NFKB1* (most significant association with HHV-6 [IE1A]; beta = −0.10; $p = 1.30 \times 10^{-10}$), the extended MHC region (EBV [EBNA-1]; beta = −0.32; $p = 6.90 \times 10^{-97}$), the IGH locus (HPV-16 [E6]; beta = 0.19; $p = 1.60 \times 10^{-10}$), and *FUT2* (JC polyomavirus [VP1]; beta = −0.13; $p = 1.70 \times 10^{-21}$), which were associated with antibody response to multiple antigens and where at least one association signal passed Bonferroni correction ($p < 1.1 \times 10^{-9}$). All four genetic regions are well recognized to drive differences in infection susceptibility.[10,11,14–17,23,50]

Associations were observed within the extended MHC region, centered mainly around human leukocyte antigen (HLA) genes, for at least one antigen from each of the 20 pathogens, with associations for human CMV, *Chlamydia trachomatis*, EBV, HHV-6, HHV-7, HPV-16, HSV-1 and -2, HTLV-1, JCV, MCV, *Toxoplasma gondii* (*T. gondii*), and VZV reaching genome-wide significance (Table S7). This suggests that, with adequate power, we are now able to resolve HLA associations with responses to antigens considered

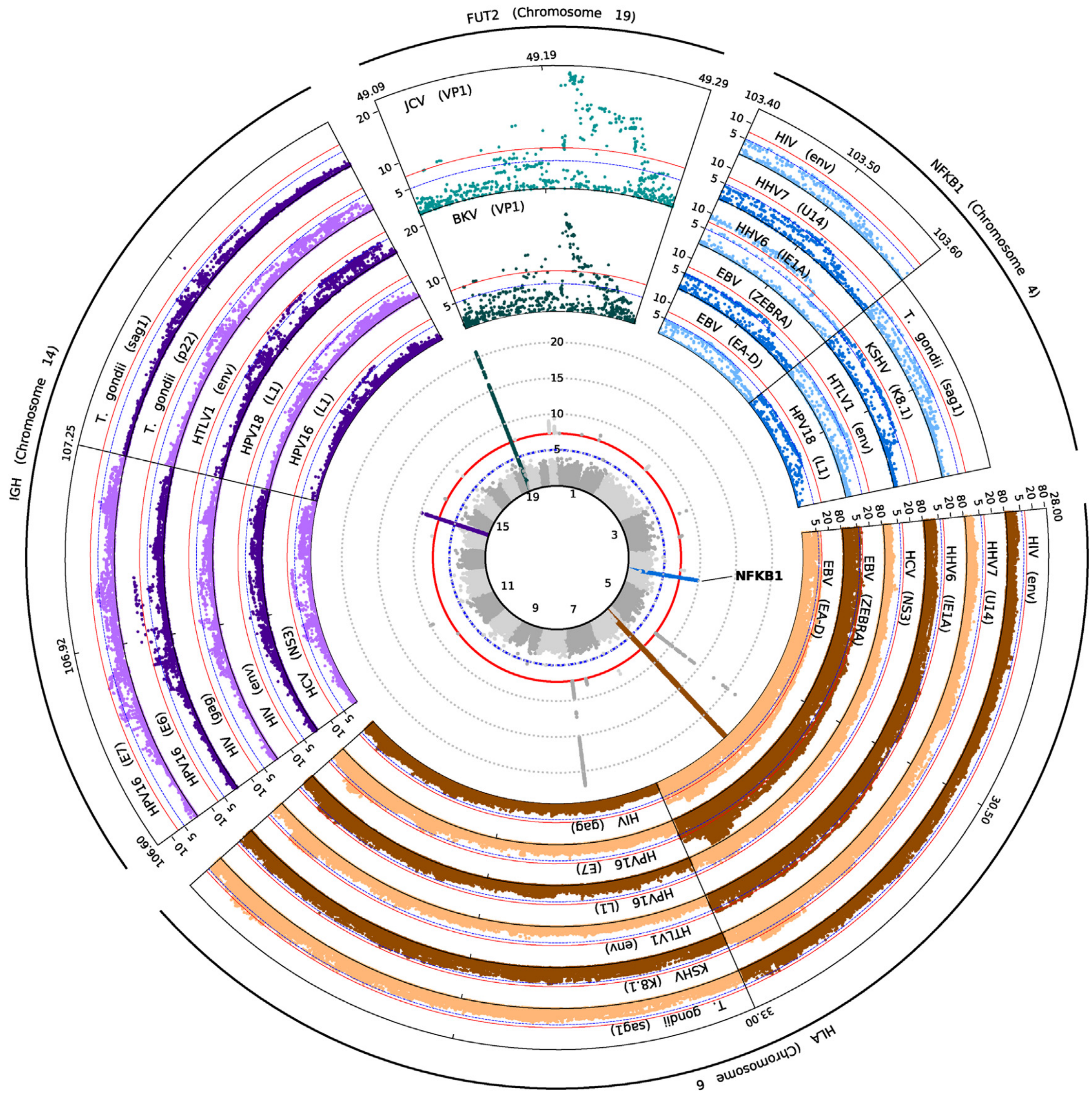

Figure 1. ***NFKB1* is a key locus affecting antibody response and risk of infection and inflammatory disease**
Central inset: stacked Manhattan plot of association statistics for antibody response to 45 pathogen-derived antigens. Signals for the four loci with multiple associations are colored: *NFKB1* (blue), the MHC locus (brown), the IGH locus (purple), and *FUT2* (teal). Surrounding insets: Regional association plots for specific antigens demonstrating signals at each of the four highlighted loci. Y axis (all plots) is $-\log_{10}(P)$.

most clinically relevant (such as EBNA-1 and EA for EBV; Figure 1), such as those described from the same UK Biobank cohort.[16,17,23]

NFKB1 is associated with antibody responses against multiple pathogens

Of particular interest is the identification of multiple variants in *NFKB1* as a common locus associated with antibody responses across multiple pathogens and pathogen types, including EBV, HPV-18, HIV, HTLV-1, KSHV, and *T. gondii*. To confirm the likely contribution of this locus to multiple infectious antigen responses, we replicated this association, demonstrating a consistent direction of effect for three antigens (EBV [EA-D] beta = –0.12, p = 9.15×10^{-7}; EBV [ZEBRA] beta = –0.12, p = 7.20×10^{-7}; and HHV7 [U14] beta = –0.06, p = 1.81×10^{-2}) using an independently recruited cohort (CoLaus/PsyCoLaus[16]) where data using the same Multiplex Serology technology was available but with a different set of antigens (Tables 1 and S8).

Using a combination of fine mapping and examination of possible variant effects, we identified the most-likely causative variant to be rs28362491, a 4 bp insertion-deletion (indel) variant within a promoter region upstream of *NFKB1*. The rs28362491 deletion is proposed to disrupt a promoter binding site, lowering *NFKB1* expression.[51] We found rs28362491 to be part of a cluster of tightly linked SNPs ($R^2 > 0.8$) predicted to contain at least one causal variant for 7 antigens: EBV (EAD), HHV-6 (IE1A), HHV-7 (U14), HPV-18 (L1), HIV (env), KSHV (K8.1), and *T. gondii*

Table 1. UK Biobank and CoLaus/PsyCoLaus meta-analysis results for rs28362491

	UK Biobank (n = 9611)				CoLaus/PsyCoLaus (n = 4216)				Meta-analysis		
Phenotype	Seropositive	beta	SE	P	Seropositive	beta	SE	P	beta	SE	P
EBV (EAD)	8,278	−0.059	0.014	3.20×10^{-5}	3,257	−0.117	0.024	9.15×10^{-7}	−0.074	0.012	1.13×10^{-9}
EBV (ZEBRA)	8,763	−0.062	0.014	1.30×10^{-5}	3,718	−0.116	0.023	7.20×10^{-7}	−0.076	0.012	3.01×10^{-10}
HHV6 (IE1A)	7,469	−0.086	0.015	2.90×10^{-9}	766	−0.03	0.024	2.03×10^{-1}	−0.071	0.012	1.03×10^{-8}
HHV7 (U14)	9,103	−0.057	0.014	5.00×10^{-5}	2,244	−0.055	0.023	1.81×10^{-2}	−0.057	0.012	2.68×10^{-6}
KSHV (K8.1)	305	−0.069	0.014	1.50×10^{-6}	58	0.035	0.024	1.47×10^{-1}	−0.042	0.012	7.40×10^{-4}
T. gondii (sag1)	2,190	−0.063	0.014	1.20×10^{-5}	1,328	−0.004	0.023	8.73×10^{-1}	−0.047	0.012	1.33×10^{-4}

(sag1). A Bayesian-based multivariate GWAS using these phenotypes confirmed the presence of a shared association across *NFKB1* (Figure S4), with rs28362491 also demonstrating a significant association (log10 Bayes factor = 5.056), as well as shared associations across the HLA region (rs9272339; log10 Bayes factor = 25.976).

NFKB1 is one of five genes that encode the NF-κB family of transcription factors. The NF-κB family plays a critical role in induction and mediation of pro-inflammatory response to infection and for development and maintenance of blood cells and immune tissues through modulation of apoptosis. Transcriptomic profiling of immune response to pathogens suggests that *NFKB1* forms part of a larger set of genes involved in regulation of immune response to a wide range of pathogen infections.[52] Furthermore, mutations in *NFKB1* have been identified as one of the most common causes of monogenic CVID in European populations.[20,22]

In addition, rs28362491 has been associated with multiple disease phenotypes in a range of previously published genetic association studies. Examples of such traits with significant evidence of effect include coronary artery disease with consistent reports of increased odds of disease with presence of the deletion demonstrated in European[53] (odds ratio [OR] = 2.88 [95% confidence interval (CI) 1.21–6.84]; Figure 2; Table S9), Indian[54] (OR = 1.26 [1.03–1.55]), and Uyghur[55] (OR = 1.58 [1.22–2.05]) populations; risk of lung injury in acute respiratory distress syndrome in Europeans[56] (OR = 3.7 [1.8–7.9]); and ulcerative colitis in Europeans[51,57] (OR = 1.57 [1.14–2.16]).

The same region has also been found to be associated in the same direction for other traits where, although the rs28362491 variant was not reported, it is likely that the effect may co-localize. These traits include tonsillectomy[11] (OR = 1.08 [1.06–1.08]; rs230523, $R^2 = 0.759$), ankylosing spondylitis[58] (OR = 1.12 [1.08–1.16]; rs3774937, $R^2 = 0.751$), primary biliary cholangitis[59] (OR = 1.34 [1.11–1.23]; rs230534, $R^2 = 0.745$), and mouth ulcers[60] (OR = 1.03 [1.02–1.04]; rs4699030, $R^2 = 0.863$) among others (Table S9). Intriguingly, the same locus and/or variant was associated with other traits in the opposite direction, including allergic rhinitis[61] (OR = 0.96 [0.95–0.97]; rs12509403, $R^2 = 0.556$), hay fever and eczema[62] (OR = 0.96 [0.95–0.97]; rs230507, $R^2 = 0.776$), and cancer risk[63] (OR = 0.89 [0.83–0.96]; rs28362491).

NFKB1 is associated with dysregulation of inflammatory responses, balancing susceptibility to infections with risk of allergy

We found that the direction of effect for rs28362491 was consistent across 9 antigens for which we observed a signal for serum antibody responses in UK Biobank (EBV [EAD and ZEBRA], HHV-6 [IE1A], HHV-7 [U14], HPV-18 [L1], HIV [env], HTLV-1 [env], KSHV [K8.1], and *T. gondii* [sag1]), with the deletion allele (rs28362491:delATTG) being associated with lower antibody responses in UK Biobank (Figure 3). Given this observation, with our observed replication in CoLaus/PsyCoLaus and the published associations with multiple infectious or inflammatory conditions (Table S9), we further tested for the effect of rs28362491:delATTG specifically on susceptibility to infection or inflammatory disease in UK Biobank, using prevalent and incident hospital inpatient (ICD-10) or self-reported cases of infection and inflammatory conditions in 487,297 participants. While neither hospital inpatient data nor self-reporting are likely to provide a comprehensive record of infection or pathogen exposure, they provide a valuable counterpoint to help understand the relationship between antibody titers and disease.

Firstly, we replicated the previously published associations with diverse traits such as allergic rhinitis[61] and ulcerative colitis,[51,57,58,64] confirming the variant's role in a variety of traits (Figure 3; Tables S9 and S10). Then we explored the effect of the variant on reported traits related to the measured antibody responses. For example, we found that although rs28362491:delATTG was consistently associated with reduced antibody levels against all measured HPV antigens, there was an increased odds of reporting cervical intraepithelial neoplasia (OR = 1.17 [1.02–1.18]) or having an ICD code of papillomavirus (OR = 1.09 [1.02–1.34]) (Figure 3). We observed a similar pattern for EBV where the deletion was associated with reduced responses against multiple EBV antigens (e.g., EA-D: OR = 0.94 [0.92–0.97]), yet there was a trend toward increased odds of self-report of EBV infection/infectious mononucleosis (OR = 1.07 [0.98–1.18]) and multiple sclerosis (which

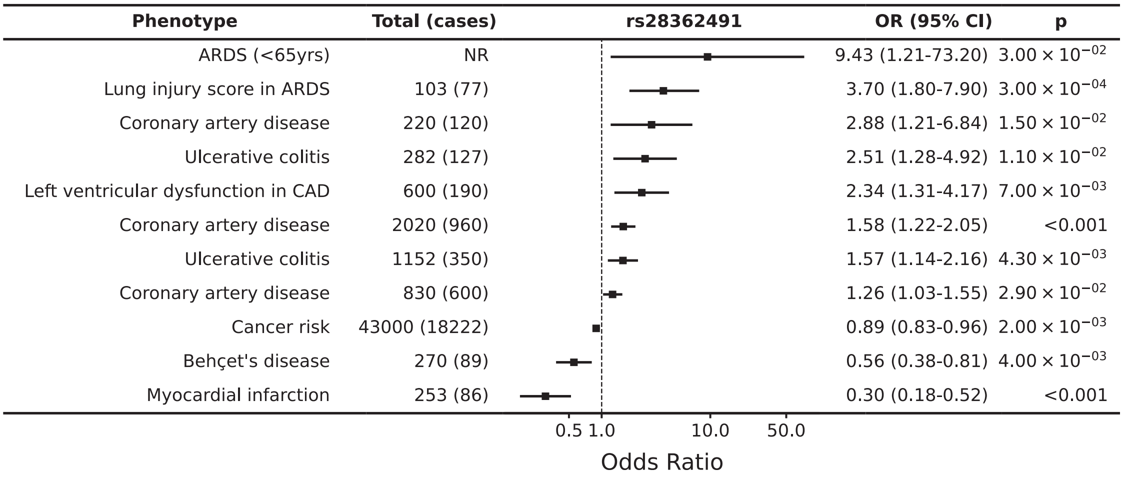

Figure 2. rs28362491:delATTG is associated with increased risk of a range of disease associated with dysregulation of inflammation
References are listed in Table S9. ARDS, acute respiratory distress syndrome; CAD, coronary artery disease.

has been plausibly causally linked with EBV infection; OR = 1.05 [0.98–1.11] in our UK Biobank data), as well as a similar effect for *H. pylori*.

We then proceeded to test the association of the variant with a wider number of infectious and inflammatory diagnoses. Out of 39 infections tested, the deletion allele increased risk of infection with influenza (OR = 1.120 [1.000–1.253]; p = 4.90 × 10^{-2}), legionella (OR = 1.524 [1.097–2.118]; p = 1.21 × 10^{-2}), and papillomavirus (OR = 1.095 [1.019–1.177]; p = 1.38 × 10^{-2}) and decreased the risk of infection with *Neisseria meningitidis* (OR = 0.704 [0.509–0.974]; p = 3.43 × 10^{-2}). Since many infections were limited in number, we also tested the effect of the deletion allele against a phenotype consisting of any infection and found that overall, there was a significant increase in risk associated with the deletion (OR = 1.014 [1.001–1.027], p = 3.31 × 10^{-2}; Table S10). Furthermore, we found that the same variant was associated with an increased risk of diseases associated with dysregulation of inflammatory responses and chronic inflammation, such as psoriasis (OR = 1.044 [1.005–1.083]; p = 2.55 × 10^{-2}). In contrast, we found that the deletion allele was protective against allergic diseases, such as allergic rhinitis (OR = 0.965 [0.949–0.981]; p = 3.57 × 10^{-5}), which are associated with acute inflammatory responses.

Altogether, these results demonstrate that this variant is associated with diverse groups of infectious or allergic diagnoses. It appears that the deletion tends to increase the risk of infectious or chronic inflammatory diagnoses possibly associated with reduced antibody responses. Conversely, the insertion is associated with increased antibody response (and thus reduced risk of infectious diagnoses) but will come at an increased risk of allergic conditions. Given these observed divergent patterns of association with risk of inflammatory and infectious or allergic disease, we proceeded to test for risk of mortality in UK Biobank participants due to allergic, infectious, or inflammatory disease. Although low in frequency given the relative immaturity of the UK Biobank cohort study, we observed similar trends of a protective effect of the deletion variant against death from allergic disease (OR = 0.846 [0.658–1.088], p = 0.192) and an increased risk of infection (OR = 1.065 [0.982–1.155], p = 0.129) and inflammation (OR = 1.038 [0.991–1.087], p = 0.117) (Table S11).

NFKB1 influences hematopoiesis and immune cell function

Having found significant evidence of the rs28362491:delATTG variant being associated with phenotypic disease endpoints and our intermediate antibody traits, we next sought to use these associations to understand the likely molecular pathways disrupted leading to increased infection risk. We first investigated the effect of rs28362491:delATTG on WBC counts and function (Table S12). Using cell-count data from UK Biobank, we observed that the deletion allele was not significantly associated with lower overall WBC counts (p = 0.96), but instead, carriers of the deletion allele had a higher total number of lymphocytes (beta = 1.98 × 10^{-2}, p = 3.30 × 10^{-24}) and eosinophils (beta = 6.49 × 10^{-3}, p = 9.30 × 10^{-4}) than those without. Individuals with the deletion also had lower counts of basophils (beta = −1.25 × 10^{-2}, p = 2.00 × 10^{-9}), monocytes (beta = −1.50 × 10^{-2}, p = 2.70 × 10^{-15}), neutrophils (beta = −7.27 × 10^{-3}, p = 2.50 × 10^{-4}), and platelets (beta = −5.79 × 10^{-3}, p = 1.30 × 10^{-3}) (Figure 4).

We next interrogated the differential expression of *NFKB1* in diverse blood cell types and found that the effect varied depending on the stimulation state of the cell. As rs28362491 is upstream of *NFKB1* and is not captured in the eQTL datasets, we used a tightly linked SNP (rs72696119, r^2 = 0.999 D′ = 0.999) in the 5′ untranslated region (UTR) of *NFKB1* as a tagging SNP for eQTL analyses. We found that rs72696119 is a *cis*-eQTL for *NFKB1*, associated with increased expression in resting monocytes (p = 1.20 × 10^{-2}), neutrophils (p = 8.00 × 10^{-3}), NK cells

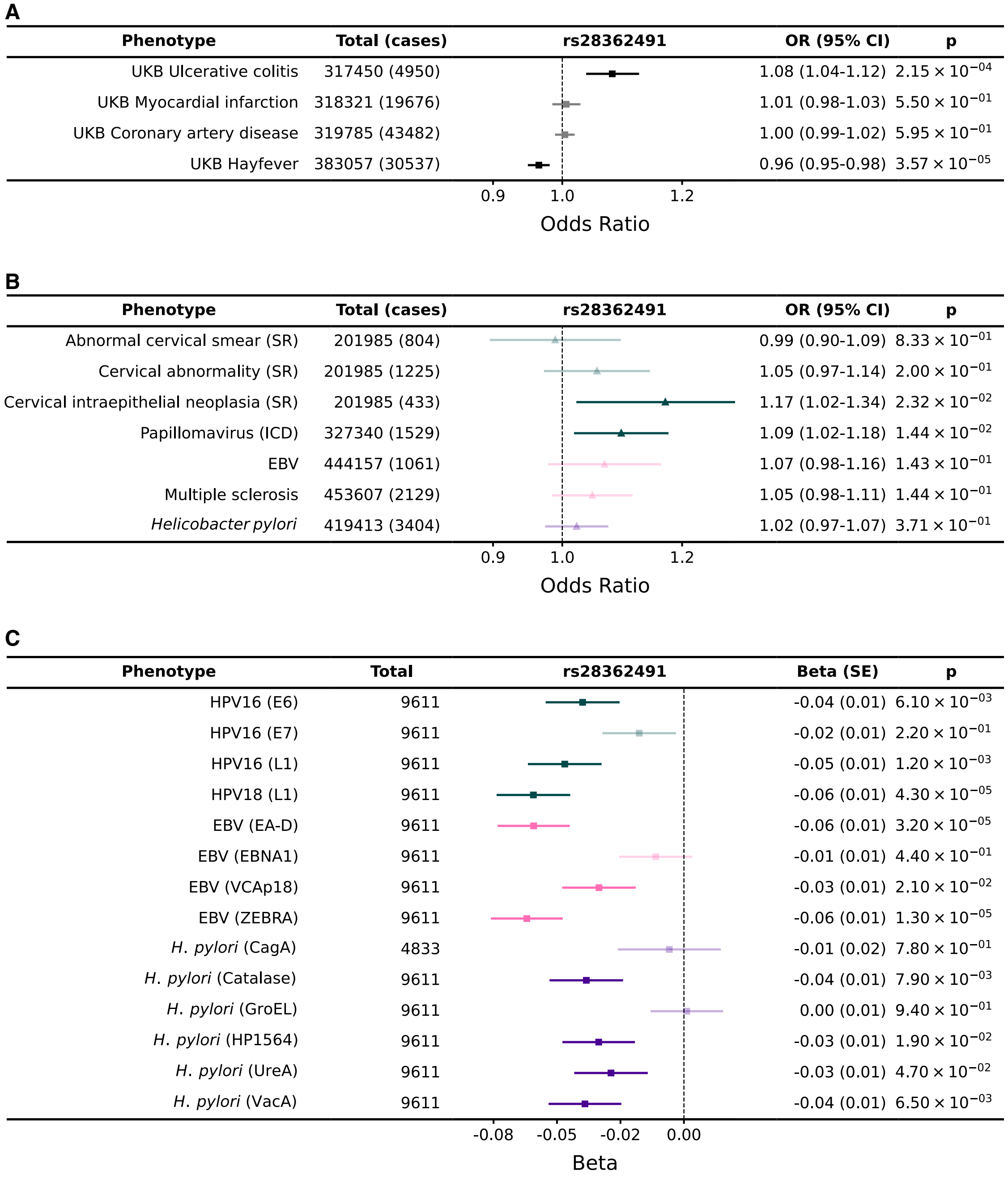

Figure 3. rs28362491:delATTG is associated with increased risk of a range of infections and disease associated with dysregulation of inflammation and protective against exacerbated immune responses and allergy
(A) Replication of published associations between rs28362491 and disease in UK Biobank.
(B) Odds ratios and 95% confidence intervals for association between rs28362491 and 7 health-record-derived disease traits derived from health record data (ICD-10 and self-report).
(C) Regression coefficients (betas) for association between rs28362491 and normalized antibody response against 14 antigens with equivalent ICD-10 or self-reported phenotypes in (B). Phenotypes in (B) and (C) are colored by pathogen: papillomavirus (teal), EBV (pink), and *H. pylori* (purple). Solid colors indicate traits with a $p < 0.05$. ICD, phenotype is derived from ICD-10 health record data; SR, phenotype is derived from self-reported health record data.

($p = 9.23 \times 10^{-4}$), and decreased expression in naive $CD4^+$ and $CD8^+$ T cells ($p = 3.50 \times 10^{-4}$ and $p = 1.40 \times 10^{-3}$) but not associated with differential expression in B cells ($p = 0.99$). All significant loci in all tested analyses were found to colocalize (e.g., HHV-6 [IE1A] and lymphocyte count: $PP_{(H4)} = 1.45 \times 10^{-7}$; Table S13).

As blood cell counts are available for the full UK Biobank cohort, we used Mendelian randomization analysis to test for causal links between the rs28362491:delATTG variant, blood cell traits, and measured antibody responses. To maximize power for these associations, given the small degree of sample overlap relative to the total number of

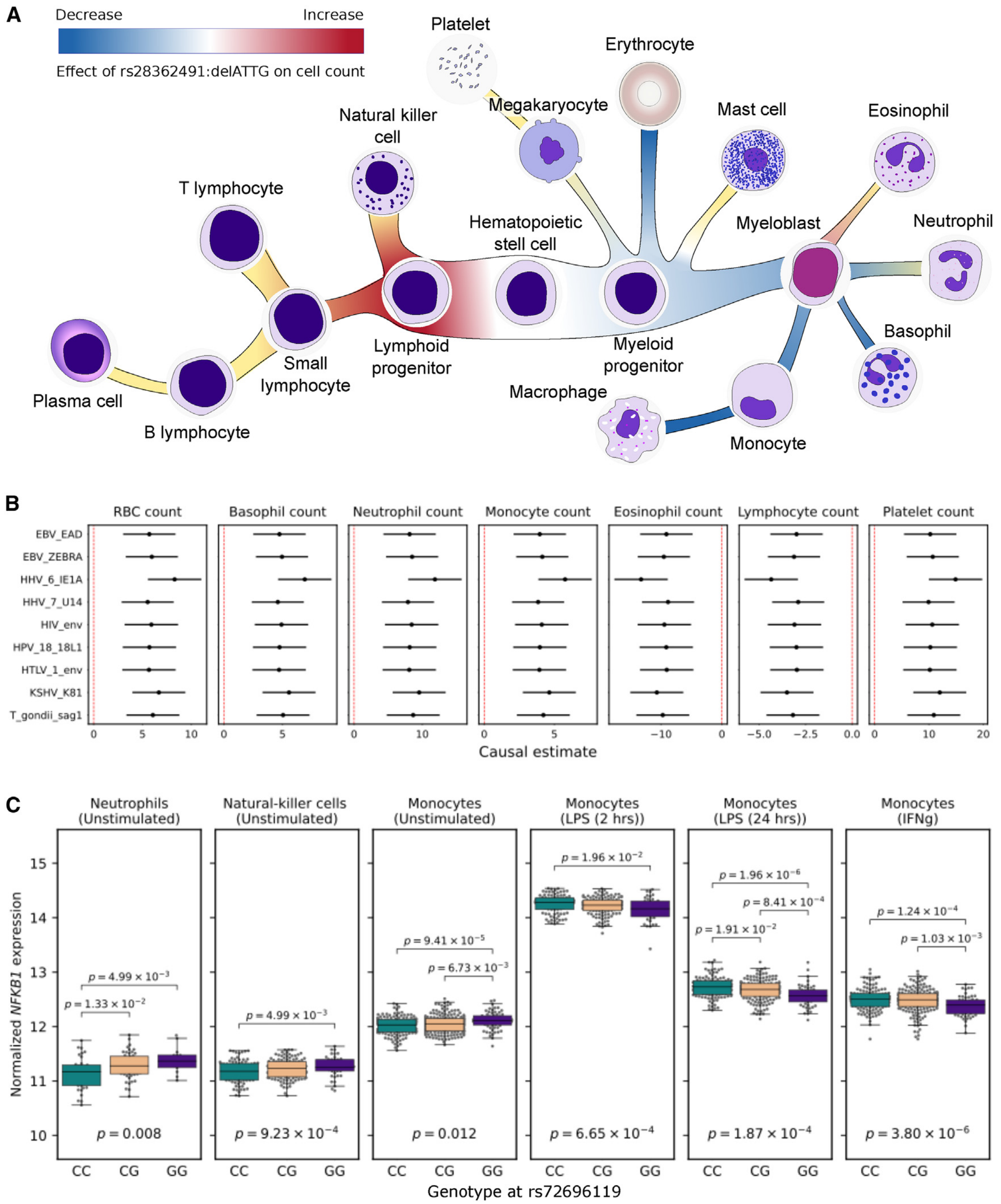

Figure 4. rs28362491 variation is also associated with differential hematopoiesis and *NFKB1* expression in white blood cells

(A) Effect of rs28362491:delATTG on WBC counts, indicated by red-blue shading. Yellow shaded regions indicate a lack of information on cell counts (mast cells, B and T cells) or signals that did not reach genome-wide significance (NK cells, platelets, neutrophils, and eosinophils).

(B) Causal estimates from two-sample Mendelian randomization analyses examining the effect of normalized blood cell counts on antibody responses.

(C) Differential expression by genotype at tightly linked 5′UTR SNP rs72696119 (delATTG linked with G), eQTL p values, and p values for pairwise t tests for differential expression by genotype. Expression for B (no significant difference) and T cells (individual level data not available) not shown.

individuals in the exposure (blood cell traits) dataset, we carried out a two-sample MR analysis. Two-sample MR supported a causal effect between blood cell counts and antibody response levels, consistent with direction of effect of the rs28362491:delATTG variant in the corresponding GWAS data. Increased RBC, basophil, neutrophil, monocyte, and platelet counts all associated with higher antibody responses, and increased lymphocyte and eosinophil counts associated with lower antibody responses (Figure 4; Table S14). Statistical power for two-sample MR for blood

cell traits and antibody responses was between 99% and 100% (Table S15), suggesting we had adequate power for these traits.

We did not identify any other significant associations between normalized blood cell counts and infection or inflammation-related disease (Table S16). However, we did not have sufficient power to deter a causal effect between these traits (2.5%–3%, Table S17). F statistics for the full cohort ranged from 103.028 for normalized lymphocyte count to 10.414 for platelet counts (Table S18).

Discussion

The genetic architecture underlying susceptibility to a host of infectious diseases is being resolved to greater detail. Loci, including the MHC, immunoglobulin loci, and *FUT2*, are becoming associated with a larger number of infectious traits when detected through multiple study formats, either through case-control analyses of clinical disease definition, self-report, or antigen-specific diagnostics.[15–17,19,23] Here, we identify rs28362491 in the promoter region of *NFKB1*, which is common in all populations (global allele frequency[del] = 0.42), that incurs a subtle, yet demonstrable effect across both the UK Biobank population and, where available, other population datasets, modulating the risk of a range of infections.

As expected, given the known pleiotropy of NF-κB signaling, our study provides significant evidence of the delicate balance between immune response to infection and inflammatory processes, where disruptions can tip the balance toward exacerbated immune responses and allergy or toward increased risk of infection and impaired resolution of inflammation. There are likely multiple mechanisms underlying this effect, involving differential hematopoiesis and immunoglobulin class switching driven by modulation of *NFKB1* gene expression.

On the basis of our functional work, we suggest that reduction of *NFKB1* expression, driven by the deletion variant, favors development of lymphoid progenitor cells and lymphoid cell lineages over myeloid lineages during hematopoiesis. NF-κB activity regulates B cell development and maturation and is important for T cell co-stimulation.[65] Despite moderate increases in the relative number of circulating lymphocytes, reduced *NFKB1* expression in T cells may result in impaired T cell function, thereby making them less responsive to infection. Variants tightly linked to rs28362491 have been associated with a reduced IgA/IgG:IgM ratio (Table S9), suggesting that reduced *NFKB1* expression may also affect B cell class switching during activation,[13,22] leading to the reduced IgG antibody levels observed in our study.

Interestingly, carriage of rs28362491:delATTG was associated with higher levels of *NFKB1* gene expression in all cell types except B and T cells (Figure 4). This was unexpected given that the deletion allele results in the disruption of a promoter binding site. This effect was reversed in monocytes stimulated with IFNγ or LPS, where lower *NFKB1* expression was observed after stimulation. Although the mechanisms for this change in expression profile are unclear, it might also be related to *NFKB1* self-regulation, as with the dysregulation of inflammation, where disruption of *NFKB1* transcription disrupts NF-κB activity, resulting in altered expression profiles in individuals carrying the deletion allele. Our results support the role of *NFKB1* as a master regulator of cellular transcription that is finely balanced depending on cell state and stimulation.

The observed inverse effect in allergy compared with infection and inflammatory disease may be a result of impaired resolution of inflammation stemming from a dysregulation of *NFKB1* expression as a result of disrupted promoter binding. NF-κB family members and downstream gene products play a key role in induction and regulation of inflammatory responses, and as such, are tightly regulated, possibly by relative ratios of the NF-κB p50-p65 (*NFKB1* encodes p50, p65 is encoded by *RELA*) and p50-p50 dimers.[54] Taken together with our cellular findings, it would appear that in the absence of haploinsufficiency, variation and regulation of expression is more important than overall *NFKB1* activity in altering risk of infection or inflammatory disease. However, follow-up studies will be required to fully dissect the effects of mild disruptions to NF-κB pathways, particularly since many of our observations are based on cross-sectional analyses, and therefore, longitudinal analyses should be prioritized for the future.

Although we present evidence from a variety of sources and study formats in support of our observation that strengthens the credibility of the association, our work does not prove definitively that, firstly, rs28362491:delATTG is the primary driving variant, nor that it is causal in all phenotypes studied. However, our fine-mapping methods would make it the most-likely candidate, and historic transcription binding studies would make it the most plausible functional modulator of expression. Causal inference through Mendelian randomization supports a possible link between hematopoietic pathways, cell function, and antibody responses, but the small effect sizes and variance explained by the rs28362491:delATTG variant mean that all but the most robust effects are likely to be missed in such analysis. Furthermore, these analyses rely on the assumption that the traits selected, and directionality of causation are correctly identified. Given the complexity of the NF-κB pathways and the complex nature of immune system interactions, it is possible that this assumption may not hold for all tested causal paths or all subsets of biobank participants at the time of data collection. As such, deeper validation of our hypothesis of mechanism will be highly challenging to dissect.

Our ability to dissect the relationship between antibody titer and pathogen-associated disease is limited by the small size of the current data subset and the low number of confirmed infections within the UK Biobank samples. While the multiplex serology platform allows us to capture

a measure of cumulative pathogen exposure, there are additional factors that will also affect the peak magnitude and speed of waning of antibody levels over time, including age, time since exposure or infection, chronic carriage of pathogens, and reactivation of latent infections. Longitudinal studies will provide better insights into how antibody titers change over time and with subsequent infection, which UK Biobank does offer opportunities to inspect, owing to follow up samples being available. We also recognize that the UK Biobank cohort is predominantly of European descent, and therefore not necessarily representative of the wider UK population.[66] As such, while we are able to draw correlations between antibody titers and reported disease, future studies should draw from a much wider cohort including participants of non-European descent, and individuals with known history of exposure.

Identifying rs28362491:delATTG as a robust association with the multiple traits presented here has required data from a substantial number of individuals demonstrating that, although the effect of the variant at an individual scale may be subtle, the variation is very important on a population level, influencing susceptibility to a range of diseases important to human health. These findings are likely to be important for future disease prediction scores and potentially from a therapeutics perspective building on our knowledge of the NF-κB complex from primary immunodeficiency work where rare but large effect variants contribute to disease susceptibility. Our findings suggest the NF-κB pathway will be difficult to target therapeutically since an altered balance will encourage either the development of infection or allergy. However, an acute disruption in favor of increased expression in cases of, for example, adjuvant use during vaccination could offer an opportunity for some infectious disease prevention on a public health scale.

Web resources

Zenodo, A common NFKB1 variant detected through antibody analysis in UK Biobank predicts risk of infection and allergy: Summary statistics – Serology, https://doi.org/10.5281/zenodo.7347714

Zenodo, A common NFKB1 variant detected through antibody analysis in UK Biobank predicts risk of infection and allergy: Summary statistics - Health records, https://doi.org/10.5281/zenodo.7347792

UK Biobank, https://www.ukbiobank.ac.uk/

CoLaus/PsyCoLaus, https://www.colaus-psycolaus.ch/

Supplemental information

Supplemental information can be found online at https://doi.org/10.1016/j.ajhg.2023.12.013.

Acknowledgments

We thank the UK Biobank participants and all individuals involved in recruitment, data and sample collection, and data curation and release. This research has been conducted using the UK Biobank Resource under application number 43920. This research has been conducted using data from the CoLaus/PsyCoLaus Study.

A.J.M. was supported by a Wellcome Trust Fellowship with ref. 106289/Z/14/Z and the National Institute for Health Research (NIHR) Oxford Biomedical Research Centre (BRC) and Academy of Medical Sciences Starter Grant (SGL024\1096). A.Y.C. was supported by the Research Councils UK Newton Fund Award with reference MR/N028937/1. A.J.-K. and A.M.-E. were supported by The Mexican Biobank Project (CONACYT-Newton Fund grant number FONCICYT/50/2016). J.J.G. is funded by a National Institute for Health Research (NIHR) Clinical Lectureship. J.F. was supported by the Swiss National Science Foundation, grant #175603. The research was supported by the Wellcome Trust Core Award grant number 203141/Z/16/Z with additional support from the NIHR Oxford BRC. The views expressed are those of the authors and not necessarily those of the NHS, the NIHR, or the Department of Health.

Author contributions

A.Y.C. and A.J.-K. carried out the GWAS of antibody traits; A.Y.C. performed additional analysis of *NFKB1*-related traits. N.B., M.H., T.J.L., T.W., and A.J.M. performed the antibody data generation, and N.B., T.J.L., T.W., and A.J.M. curated the data. A.C., M.H., and T.J.L. contributed to the methods development. J.J.G., B.P.F., and J.C.K. carried out analysis of the eQTL data. F.H. and J.F. carried out analysis of the CoLaus/PsyCoLaus cohort. T.W., A.V.S.H., and A.J.M. designed the experiments. G.M., A.M.-E., T.W., A.V.S.H., and A.J.M. supervised the research and provided funding.

Declaration of interests

The authors declare no competing interests.

Received: January 6, 2023
Accepted: December 10, 2023
Published: January 16, 2024

References

1. Davila, S., Wright, V.J., Khor, C.C., Sim, K.S., Binder, A., Breunis, W.B., Inwald, D., Nadel, S., Betts, H., Carrol, E.D., et al. (2010). Genome-wide association study identifies variants in the CFH region associated with host susceptibility to meningococcal disease. Nat. Genet. *42*, 772–776.
2. Gilchrist, J.J., Rautanen, A., Fairfax, B.P., Mills, T.C., Naranbhai, V., Trochet, H., Pirinen, M., Muthumbi, E., Mwarumba, S., Njuguna, P., et al. (2018). Risk of nontyphoidal Salmonella bacteraemia in African children is modified by STAT4. Nat. Commun. *9*, 1014.
3. Initiative, C.-H.G. (2021). Mapping the human genetic architecture of COVID-19. Nature *2021*, 1–8.
4. Pairo-Castineira, E., Clohisey, S., Klaric, L., Bretherick, A.D., Rawlik, K., Pasko, D., Walker, S., Parkinson, N., Fourman, M.H., Russell, C.D., et al. (2020). Genetic mechanisms of critical illness in COVID-19. Nature *591*, 92–98.
5. The Severe Covid, G.G. (2020). Genomewide Association Study of Severe Covid-19 with Respiratory Failure. N. Engl. J. Med. *383*, 1522–1534.
6. Vergara, C., Thio, C.L., Johnson, E., Kral, A.H., O'Brien, T.R., Goedert, J.J., Mangia, A., Piazzolla, V., Mehta, S.H., Kirk,

G.D., et al. (2019). Multi-Ancestry Genome-Wide Association Study of Spontaneous Clearance of Hepatitis C Virus. Gastroenterology *156*, 1496–1507.e7.
7. Wang, Z., Sun, Y., Fu, X., Yu, G., Wang, C., Bao, F., Yue, Z., Li, J., Sun, L., Irwanto, A., et al. (2016). A large-scale genome-wide association and meta-analysis identified four novel susceptibility loci for leprosy. Nat. Commun. *7*, 13760.
8. Zhang, F., Liu, H., Chen, S., Low, H., Sun, L., Cui, Y., Chu, T., Li, Y., Fu, X., Yu, Y., et al. (2011). Identification of two new loci at IL23R and RAB32 that influence susceptibility to leprosy. Nat. Genet. *43*, 1247–1251.
9. Duggal, P., Thio, C.L., Wojcik, G.L., Goedert, J.J., Mangia, A., Latanich, R., Kim, A.Y., Lauer, G.M., Chung, R.T., Peters, M.G., et al. (2013). Genome-Wide Association Study of Spontaneous Resolution of Hepatitis C Virus Infection: Data From Multiple Cohorts. Ann. Intern. Med. *158*, 235–245.
10. Dunstan, S.J., Hue, N.T., Han, B., Li, Z., Tram, T.T.B., Sim, K.S., Parry, C.M., Chinh, N.T., Vinh, H., Lan, N.P.H., et al. (2014). Variation at HLA-DRB1 is associated with resistance to enteric fever. Nat. Genet. *46*, 1333–1336.
11. Tian, C., Hromatka, B.S., Kiefer, A.K., Eriksson, N., Noble, S.M., Tung, J.Y., and Hinds, D.A. (2017). Genome-wide association and HLA region fine-mapping studies identify susceptibility loci for multiple common infections. Nat. Commun. *8*, 599.
12. VanBlargan, L.A., Goo, L., and Pierson, T.C. (2016). Deconstructing the Antiviral Neutralizing-Antibody Response: Implications for Vaccine Development and Immunity. Microbiol. Mol. Biol. Rev. *80*, 989–1010.
13. Jonsson, S., Sveinbjornsson, G., de Lapuente Portilla, A.L., Swaminathan, B., Plomp, R., Dekkers, G., Ajore, R., Ali, M., Bentlage, A.E.H., Elmér, E., et al. (2017). Identification of sequence variants influencing immunoglobulin levels. Nat. Genet. *49*, 1182–1191.
14. Png, E., Thalamuthu, A., Ong, R.T.H., Snippe, H., Boland, G.J., and Seielstad, M. (2011). A genome-wide association study of hepatitis B vaccine response in an Indonesian population reveals multiple independent risk variants in the HLA region. Hum. Mol. Genet. *20*, 3893–3898.
15. Hammer, C., Begemann, M., McLaren, P.J., Bartha, I., Michel, A., Klose, B., Schmitt, C., Waterboer, T., Pawlita, M., Schulz, T.F., et al. (2015). Amino Acid Variation in HLA Class II Proteins Is a Major Determinant of Humoral Response to Common Viruses. Am. J. Hum. Genet. *97*, 738–743.
16. Hodel, F., Chong, A.Y., Scepanovic, P., Xu, Z.M., Naret, O., Thorball, C.W., Rüeger, S., Marques-Vidal, P., Vollenweider, P., Begemann, M., et al. (2021). Human genomics of the humoral immune response against polyomaviruses. Virus Evol. *7*, veab058.
17. Kachuri, L., Francis, S.S., Morrison, M.L., Wendt, G.A., Bossé, Y., Cavazos, T.B., Rashkin, S.R., Ziv, E., and Witte, J.S. (2020). The landscape of host genetic factors involved in immune response to common viral infections. Genome Med. *12*, 93.
18. Andreu-Sánchez, S., Bourgonje, A.R., Vogl, T., Kurilshikov, A., Leviatan, S., Ruiz-Moreno, A.J., Hu, S., Sinha, T., Vich Vila, A., Klompus, S., et al. (2023). Phage display sequencing reveals that genetic, environmental, and intrinsic factors influence variation of human antibody epitope repertoire. Immunity *56*, 1376–1392.e1378.
19. Venkataraman, T., Valencia, C., Mangino, M., Morgenlander, W., Clipman, S.J., Liechti, T., Valencia, A., Christofidou, P., Spector, T., Roederer, M., et al. (2022). Analysis of antibody binding specificities in twin and SNP-genotyped cohorts reveals that antiviral antibody epitope selection is a heritable trait. Immunity *55*, 174–184.e5.
20. Hayden, M.S., and Ghosh, S. (2011). NF-kappaB in immunobiology. Cell Res. *21*, 223–244.
21. Hayden, M.S., West, A.P., and Ghosh, S. (2006). NF-kappaB and the immune response. Oncogene *25*, 6758–6780.
22. Tuijnenburg, P., Lango Allen, H., Burns, S.O., Greene, D., Jansen, M.H., Staples, E., Stephens, J., Carss, K.J., Biasci, D., Baxendale, H., et al. (2018). Loss-of-function nuclear factor kappaB subunit 1 (NFKB1) variants are the most common monogenic cause of common variable immunodeficiency in Europeans. J. Allergy Clin. Immunol. *142*, 1285–1296.
23. Butler-Laporte, G., Kreuzer, D., Nakanishi, T., Harroud, A., Forgetta, V., and Richards, J.B. (2020). Genetic Determinants of Antibody-Mediated Immune Responses to Infectious Diseases Agents: A Genome-Wide and HLA Association Study. Open Forum Infect. Dis. *7*, ofaa450.
24. Mentzer, A.J., Brenner, N., Allen, N., Littlejohns, T.J., Chong, A.Y., Cortes, A., Almond, R., Hill, M., Sheard, S., McVean, G., et al. (2022). Identification of host–pathogen-disease relationships using a scalable multiplex serology platform in UK Biobank. Nat. Commun. *13*, 1818.
25. Waterboer, T., Sehr, P., Michael, K.M., Franceschi, S., Nieland, J.D., Joos, T.O., Templin, M.F., and Pawlita, M. (2005). Multiplex Human Papillomavirus Serology Based on In Situ–Purified Glutathione S-Transferase Fusion Proteins. Clin. Chem. *51*, 1845–1853.
26. Bycroft, C., Freeman, C., Petkova, D., Band, G., Elliott, L.T., Sharp, K., Motyer, A., Vukcevic, D., Delaneau, O., O'Connell, J., et al. (2018). The UK Biobank resource with deep phenotyping and genomic data. Nature *562*, 203–209.
27. 1000 Genomes Project Consortium, Auton, A., Brooks, L.D., Durbin, R.M., Garrison, E.P., Kang, H.M., Korbel, J.O., Marchini, J.L., McCarthy, S., McVean, G.A., and Abecasis, G.R. (2015). A global reference for human genetic variation. Nature *526*, 68–74.
28. Huang, J., Howie, B., McCarthy, S., Memari, Y., Walter, K., Min, J.L., Danecek, P., Malerba, G., Trabetti, E., Zheng, H.F., et al. (2015). Improved imputation of low-frequency and rare variants using the UK10K haplotype reference panel. Nat. Commun. *6*, 8111.
29. Loh, P.R., Danecek, P., Palamara, P.F., Fuchsberger, C., A Reshef, Y., K Finucane, H., Schoenherr, S., Forer, L., McCarthy, S., Abecasis, G.R., et al. (2016). Reference-based phasing using the Haplotype Reference Consortium panel. Nat. Genet. *48*, 1443–1448.
30. McCarthy, S., Das, S., Kretzschmar, W., Delaneau, O., Wood, A.R., Teumer, A., Kang, H.M., Fuchsberger, C., Danecek, P., Sharp, K., et al. (2016). A reference panel of 64,976 haplotypes for genotype imputation. Nat. Genet. *48*, 1279–1283.
31. Loh, P.R., Tucker, G., Bulik-Sullivan, B.K., Vilhjálmsson, B.J., Finucane, H.K., Salem, R.M., Chasman, D.I., Ridker, P.M., Neale, B.M., Berger, B., et al. (2015). Efficient Bayesian mixed-model analysis increases association power in large cohorts. Nat. Genet. *47*, 284–290.
32. Chang, C.C., Chow, C.C., Tellier, L.C., Vattikuti, S., Purcell, S.M., and Lee, J.J. (2015). Second-generation PLINK: Rising to the challenge of larger and richer datasets. GigaScience *4*, 7.
33. Yang, J., Lee, S.H., Goddard, M.E., and Visscher, P.M. (2011). GCTA: a tool for genome-wide complex trait analysis. Am. J. Hum. Genet. *88*, 76–82.

34. Benner, C., Spencer, C.C.A., Havulinna, A.S., Salomaa, V., Ripatti, S., and Pirinen, M. (2016). FINEMAP: efficient variable selection using summary data from genome-wide association studies. Bioinformatics *32*, 1493–1501.
35. The Wellcome Trust Case Control, C. (2007). Genome-wide association study of 14,000 cases of seven common diseases and 3,000 shared controls. Nature *447*, 661–678.
36. Firmann, M., Mayor, V., Vidal, P.M., Bochud, M., Pécoud, A., Hayoz, D., Paccaud, F., Preisig, M., Song, K.S., Yuan, X., et al. (2008). The CoLaus study: a population-based study to investigate the epidemiology and genetic determinants of cardiovascular risk factors and metabolic syndrome. BMC Cardiovasc. Disord. *8*, 6.
37. Han, B., and Eskin, E. (2011). Random-effects model aimed at discovering associations in meta-analysis of genome-wide association studies. Am. J. Hum. Genet. *88*, 586–598.
38. Zhou, W., Nielsen, J.B., Fritsche, L.G., Dey, R., Gabrielsen, M.E., Wolford, B.N., LeFaive, J., VandeHaar, P., Gagliano, S.A., Gifford, A., et al. (2018). Efficiently controlling for case-control imbalance and sample relatedness in large-scale genetic association studies. Nat. Genet. *50*, 1335–1341.
39. Fairfax, B.P., Humburg, P., Makino, S., Naranbhai, V., Wong, D., Lau, E., Jostins, L., Plant, K., Andrews, R., McGee, C., and Knight, J.C. (2014). Innate immune activity conditions the effect of regulatory variants upon monocyte gene expression. Science *343*, 1246949.
40. Fairfax, B.P., Makino, S., Radhakrishnan, J., Plant, K., Leslie, S., Dilthey, A., Ellis, P., Langford, C., Vannberg, F.O., and Knight, J.C. (2012). Genetics of gene expression in primary immune cells identifies cell type-specific master regulators and roles of HLA alleles. Nat. Genet. *44*, 502–510.
41. Gilchrist, J.J., Makino, S., Naranbhai, V., Sharma, P.K., Koturan, S., Tong, O., Taylor, C.A., Watson, R.A., de los Aires, A.V., Cooper, R., et al. (2022). Natural Killer cells demonstrate distinct eQTL and transcriptome-wide disease associations, highlighting their role in autoimmunity. Nat. Commun. *13*, 4073.
42. Naranbhai, V., Fairfax, B.P., Makino, S., Humburg, P., Wong, D., Ng, E., Hill, A.V.S., and Knight, J.C. (2015). Genomic modulators of gene expression in human neutrophils. Nat. Commun. *6*, 7545.
43. Schmiedel, B.J., Singh, D., Madrigal, A., Valdovino-Gonzalez, A.G., White, B.M., Zapardiel-Gonzalo, J., Ha, B., Altay, G., Greenbaum, J.A., McVicker, G., et al. (2018). Impact of Genetic Polymorphisms on Human Immune Cell Gene Expression. Cell *175*, 1701–1715.e16.
44. Wallace, C. (2021). A more accurate method for colocalisation analysis allowing for multiple causal variants. PLoS Genet. *17*, e1009440.
45. Wang, G., Sarkar, A., Carbonetto, P., and Stephens, M. (2020). A simple new approach to variable selection in regression, with application to genetic fine mapping. J. R. Stat. Soc. Series B Stat. Methodol. *82*, 1273–1300.
46. Zou, Y., Carbonetto, P., Wang, G., and Stephens, M. (2021). Fine-mapping from summary data with the “Sum of Single Effects” model. Preprint at bioRxiv.
47. Burgess, S., Davies, N.M., and Thompson, S.G. (2016). Bias due to participant overlap in two-sample Mendelian randomization. Genet. Epidemiol. *40*, 597–608.
48. Broadbent, J.R., Foley, C.N., Grant, A.J., Mason, A.M., Staley, J.R., and Burgess, S. (2020). MendelianRandomization v0.5.0: updates to an R package for performing Mendelian randomization analyses using summarized data. Wellcome Open Res. *5*, 252.
49. Yavorska, O.O., and Burgess, S. (2017). MendelianRandomization: an R package for performing Mendelian randomization analyses using summarized data. Int. J. Epidemiol. *46*, 1734–1739.
50. Scepanovic, P., Alanio, C., Hammer, C., Hodel, F., Bergstedt, J., Patin, E., Thorball, C.W., Chaturvedi, N., Charbit, B., Abel, L., et al. (2018). Human genetic variants and age are the strongest predictors of humoral immune responses to common pathogens and vaccines. Genome Med. *10*, 59.
51. Karban, A.S., Okazaki, T., Panhuysen, C.I.M., Gallegos, T., Potter, J.J., Bailey-Wilson, J.E., Silverberg, M.S., Duerr, R.H., Cho, J.H., Gregersen, P.K., et al. (2004). Functional annotation of a novel NFKB1 promoter polymorphism that increases risk for ulcerative colitis. Hum. Mol. Genet. *13*, 35–45.
52. Jenner, R.G., and Young, R.A. (2005). Insights into host responses against pathogens from transcriptional profiling. Nat. Rev. Microbiol. *3*, 281–294.
53. Seidi, A., Mirzaahmadi, S., Mahmoodi, K., and Soleiman-Soltanpour, M. (2018). The association between NFKB1 -94ATTG ins/del and NFKB1A 826C/T genetic variations and coronary artery disease risk. Mol. Biol. Res. Commun. *7*, 17–24.
54. Mishra, A., Srivastava, A., Mittal, T., Garg, N., and Mittal, B. (2013). Role of inflammatory gene polymorphisms in left ventricular dysfunction (LVD) susceptibility in coronary artery disease (CAD) patients. Cytokine *61*, 856–861.
55. Lai, H.-M., Li, X.-M., Yang, Y.-N., Ma, Y.-T., Xu, R., Pan, S., Zhai, H., Liu, F., Chen, B.-D., and Zhao, Q. (2015). Genetic Variation in NFKB1 and NFKBIA and Susceptibility to Coronary Artery Disease in a Chinese Uygur Population. PLoS One *10*, e0129144.
56. Bajwa, E.K., Cremer, P.C., Gong, M.N., Zhai, R., Su, L., Thompson, B.T., and Christiani, D.C. (2011). An NFKB1 promoter insertion/deletion polymorphism influences risk and outcome in acute respiratory distress syndrome among Caucasians. PLoS One *6*, e19469.
57. Borm, M.E.A., van Bodegraven, A.A., Mulder, C.J.J., Kraal, G., and Bouma, G. (2005). A NFKB1 promoter polymorphism is involved in susceptibility to ulcerative colitis. Int. J. Immunogenet. *32*, 401–405.
58. Ellinghaus, D., Jostins, L., Spain, S.L., Cortes, A., Bethune, J., Han, B., Park, Y.R., Raychaudhuri, S., Pouget, J.G., Hübenthal, M., et al. (2016). Analysis of five chronic inflammatory diseases identifies 27 new associations and highlights disease-specific patterns at shared loci. Nat. Genet. *48*, 510–518.
59. Kawashima, M., Hitomi, Y., Aiba, Y., Nishida, N., Kojima, K., Kawai, Y., Nakamura, H., Tanaka, A., Zeniya, M., Hashimoto, E., et al. (2017). Genome-wide association studies identify PRKCB as a novel genetic susceptibility locus for primary biliary cholangitis in the Japanese population. Hum. Mol. Genet. *26*, 650–659.
60. Dudding, T., Haworth, S., Lind, P.A., Sathirapongsasuti, J.F., 23andMe Research Team, Tung, J.Y., Mitchell, R., Colodro-Conde, L., Medland, S.E., Gordon, S., et al. (2019). Genome wide analysis for mouth ulcers identifies associations at immune regulatory loci. Nat. Commun. *10*, 1052.
61. Waage, J., Standl, M., Curtin, J.A., Jessen, L.E., Thorsen, J., Tian, C., Schoettler, N., 23andMe Research Team; and AAGC collaborators, and Flores, C., et al. (2018). Genome-wide association and HLA fine-mapping studies identify risk loci and

genetic pathways underlying allergic rhinitis. Nat. Genet. *50*, 1072–1080.

62. Johansson, Å., Rask-Andersen, M., Karlsson, T., and Ek, W.E. (2019). Genome-wide association analysis of 350 000 Caucasians from the UK Biobank identifies novel loci for asthma, hay fever and eczema. Hum. Mol. Genet. *28*, 4022–4041.
63. Wang, D., Xie, T., Xu, J., Wang, H., Zeng, W., Rao, S., Zhou, K., Pei, F., and Zhou, Z. (2016). Genetic association between NFKB1 -94 ins/del ATTG Promoter Polymorphism and cancer risk: a meta-analysis of 42 case-control studies. Sci. Rep. *6*, 30220.
64. Jostins, L., Ripke, S., Weersma, R.K., Duerr, R.H., McGovern, D.P., Hui, K.Y., Lee, J.C., Schumm, L.P., Sharma, Y., Anderson, C.A., et al. (2012). Host-microbe interactions have shaped the genetic architecture of inflammatory bowel disease. Nature *491*, 119–124.
65. Kaileh, M., and Sen, R. (2012). NF-kappaB function in B lymphocytes. Immunol. Rev. *246*, 254–271.
66. Keyes, K.M., and Westreich, D. (2019). UK Biobank, big data, and the consequences of non-representativeness. Lancet *393*, 1297.

The American Journal of Human Genetics, Volume *111*

Supplemental information

A common *NFKB1* variant detected through antibody analysis in UK Biobank predicts risk of infection and allergy

Amanda Y. Chong, Nicole Brenner, Andres Jimenez-Kaufmann, Adrian Cortes, Michael Hill, Thomas J. Littlejohns, James J. Gilchrist, Benjamin P. Fairfax, Julian C. Knight, Flavia Hodel, Jacques Fellay, Gil McVean, Andres Moreno-Estrada, Tim Waterboer, Adrian V.S. Hill, and Alexander J. Mentzer

Supplemental Figures

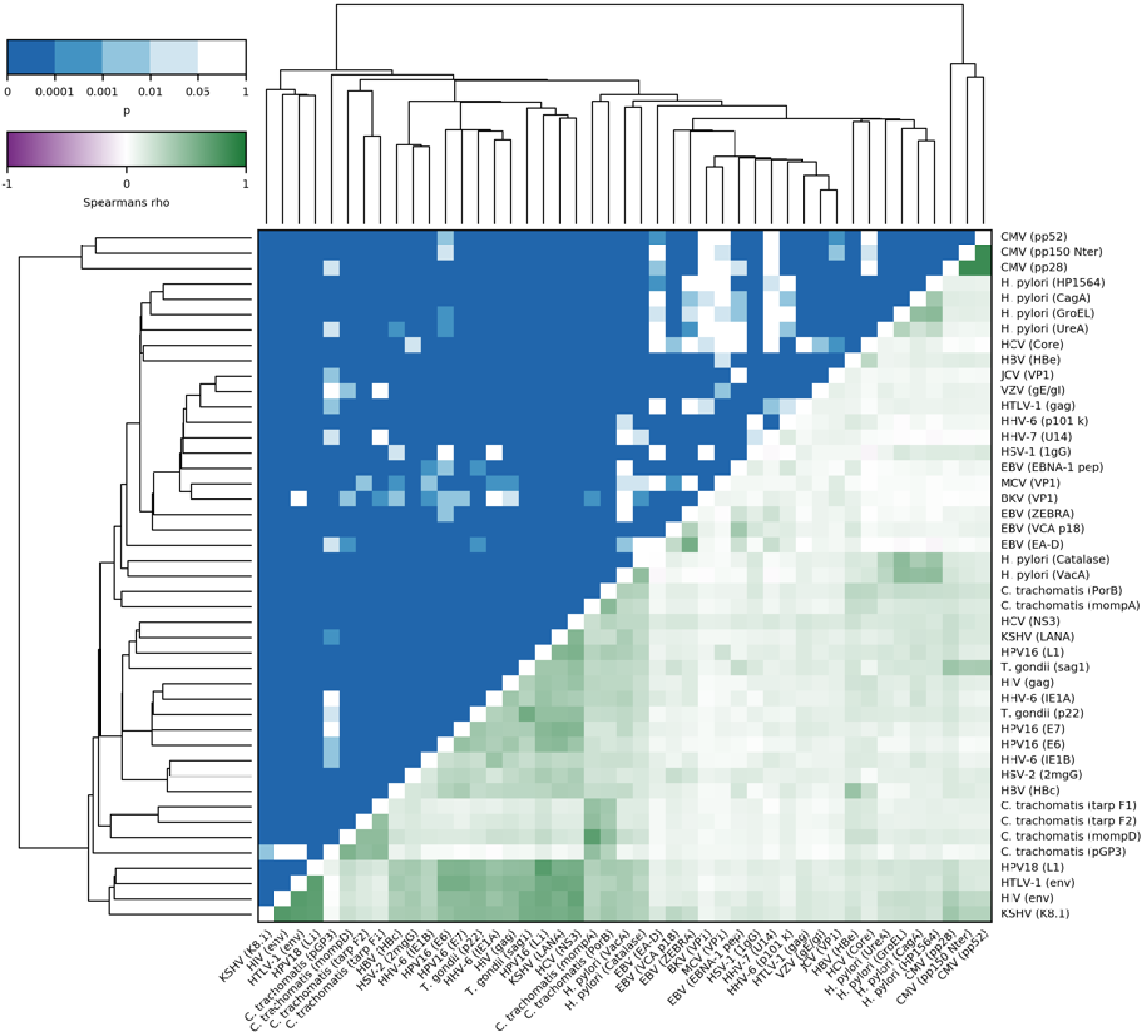

Figure S1: All antibody responses were generally positively correlated. Antibody responses to different antigens from the same pathogen tended to cluster together and were more strongly correlated with each other. No evidence of unexpected correlation between antibody traits was detected. Pairwise correlations between antibody responses are shown in the bottom triangle, p-values are indicated in the upper triangle

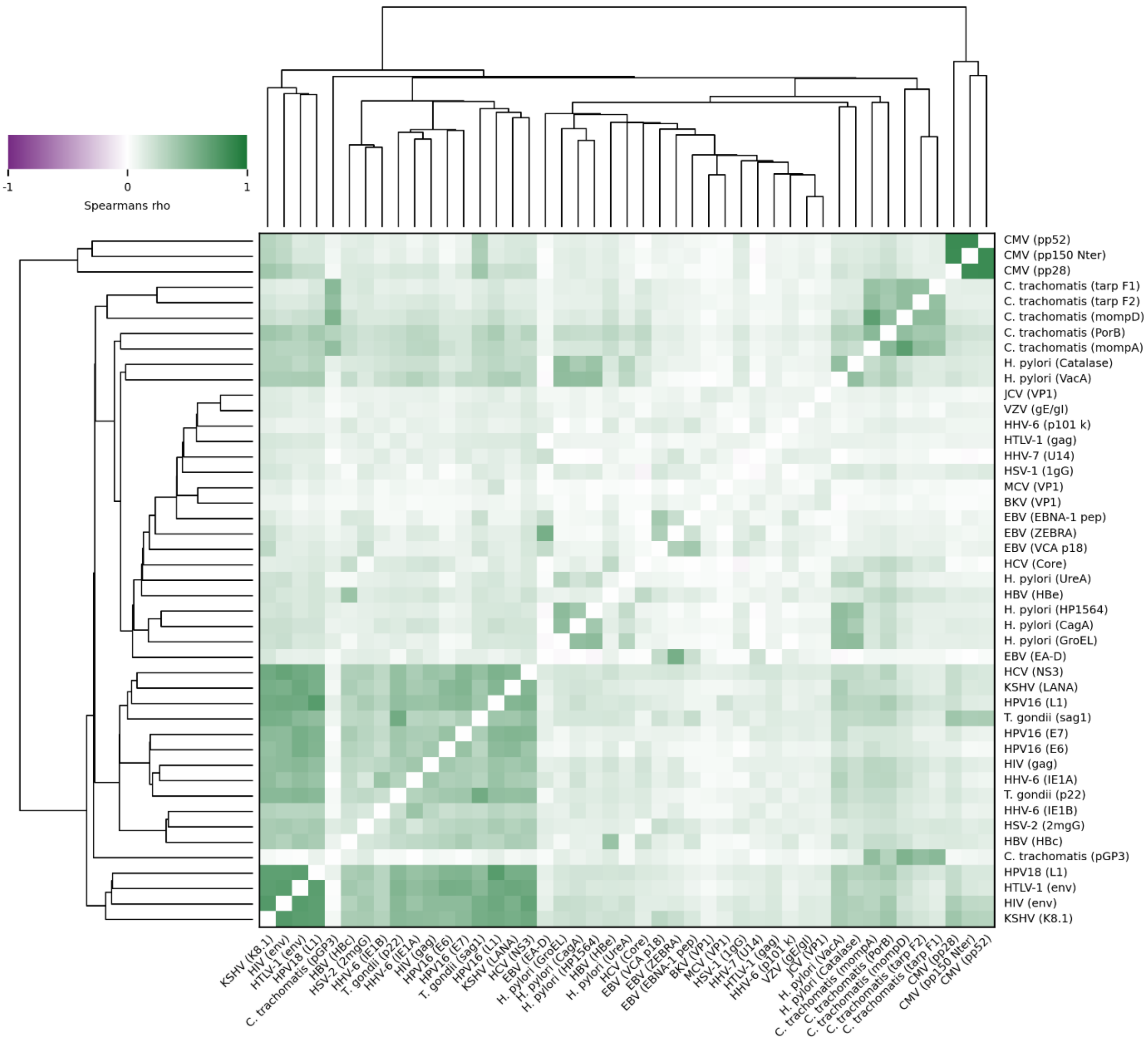

Figure S2: Antibody responses remained correlated after accounting for effects of age, sex, and lead GWAS SNPs. Pairwise correlations between antibody responses accounting for age and sex are shown in the bottom triangle, correlations accounting for age, sex, and genetic variants are indicated in the upper triangle.

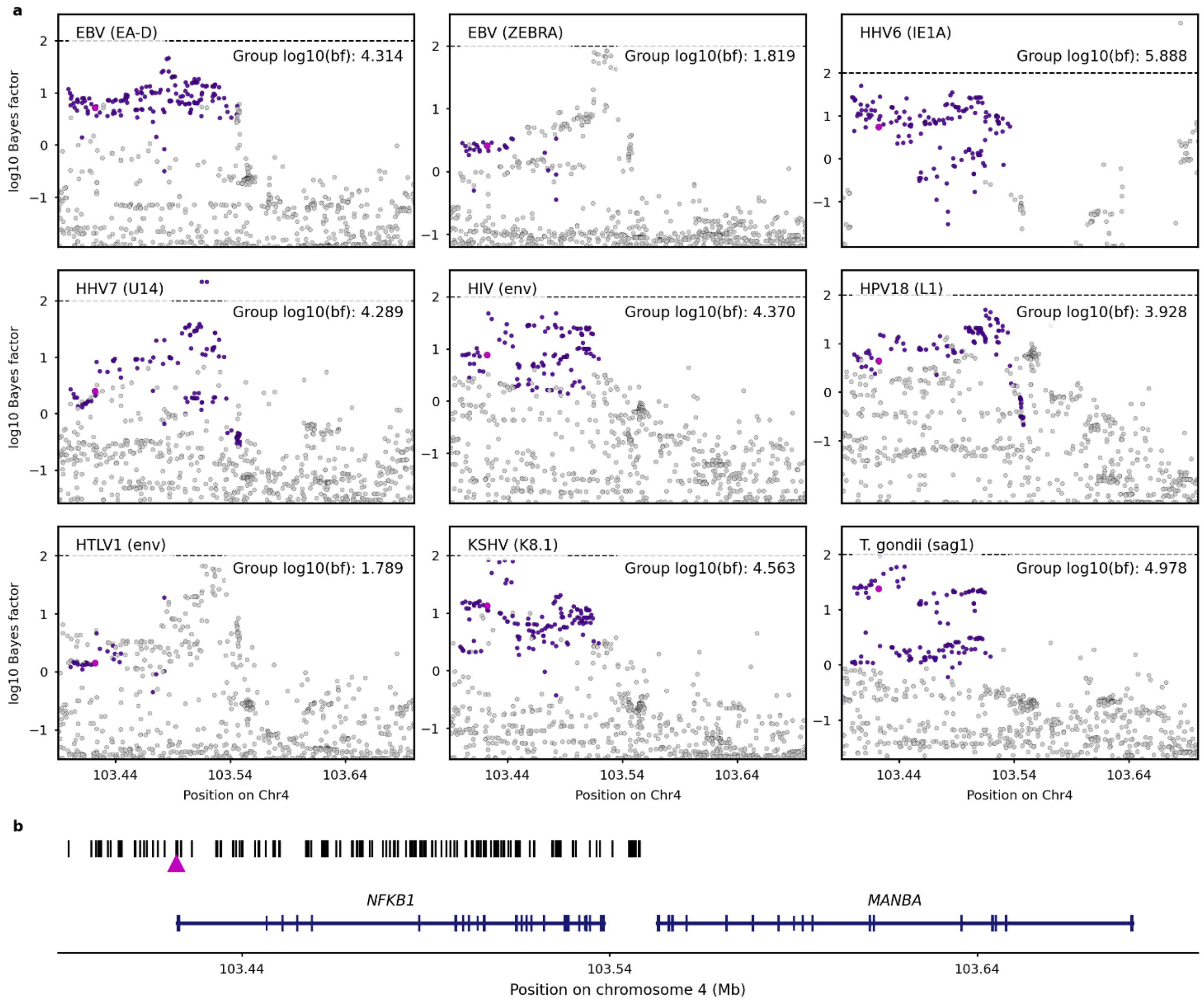

Figure S3: Associations on Chromosome 4 fine-mapped to the *NFKB1* locus. (a) Fine-mapping results for significantly associated antibody responses. Log10 Bayes factors for individual variants are shown on the y axis. Predicted causal groups of variants containing rs28362491 for each phenotype are in purple, rs28362491 is highlighted in pink. Log10 Bayes factors for the entire causal group are also shown. (b) Location of all predicted causal variants from (a) relative to *NFKB1* and *MANBA* on chromosome 4. Location of rs28362491 is indicated by a pink triangle.

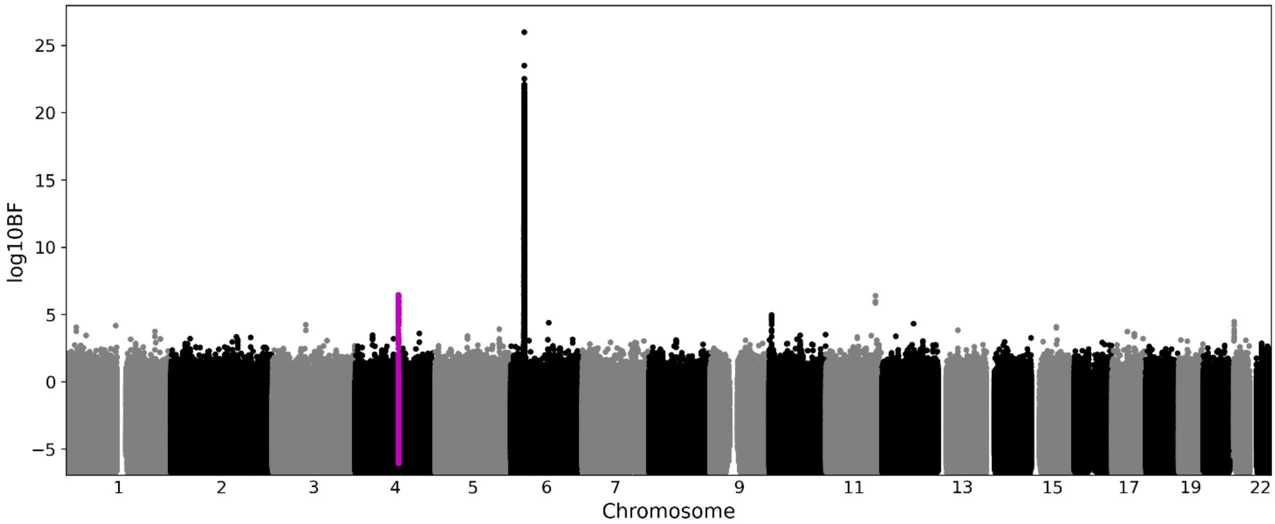

Figure S4: Bayesian-based multivariate GWAS including seven significant phenotypes supports a shared association at *NFKB1*. Included phenotypes: EBV (EAD), HHV-6 (IE1A), HHV-7 (U14), HIV (env), HPV18 (L1), KSHV (K8.1), and *T. gondii* (sag1). The *NFKB1* region is highlighted in pink.

Supplemental Tables

Table S1: Details of the 45 antigens and 20 pathogens covered in the UK Biobank serology panel

Infectious agent	Antigen	Likely Function	Reference
HSV-1	1gG	membrane glycoprotein	1
HSV-2	2mgGunique	membrane glycoprotein	1
VZV	gE/gI	envelope glycoproteins	1
EBV	VCAp18	capsid protein	1
	EBNA1	replication, latent viral infection	
	ZEBRA	replication activator	
	EA-D	replication (polymerase accessory subunit)	
CMV	pp150 (N-terminus)	tegument protein	1
	pp52	DNA binding protein	
	pp28	capsid protein	
HHV-6	IE1B	potential transactivator	Validation ongoing
	IE1A	potential transactivator	
	p101k	potential tegument protein	
HHV-7	U14	potential tegument protein	Validation ongoing
KSHV	LANA	replication and long-term persistence	Validation ongoing
	K8.1	structural glycoprotein	
HBV	HBc	core antigen	2
	HBe	soluble nucleocapsid associated antigen	
HCV	Core	structural antigen	2; 3
	NS3	protease and RNA helicase activity	
Toxoplasma gondii	p22	surface protein	2
	sag1	surface protein	
HTLV-1	Gag	structural antigen	2
	Env	structural antigen	
HIV-1	Gag	structural antigen	4
	Env	structural antigen	
BKV	VP1	major capsid protein	5-7
JCV	VP1	major capsid protein	5-7
MCV	VP1	major capsid protein	5; 7
HPV16	L1	major capsid protein	8; 9
	E6	oncogene	
	E7	oncogene	
HPV18	L1	major capsid protein	8
Chlamydia trachomatis	mompD	structural antigen	10; 11
	mompA	structural antigen	
	TarpDF1	regulation of actin recruitment	
	TarpDF2		
	PorB	porin	
	pGP3	virulence factor	
Helicobacter pylori	CagA (N-terminus)	pathogenesis	12
	VacA (C-terminus)	pathogenesis	
	HP1564	cell envelope	
	GroEL	chaperonin	
	Catalase	detoxification	
	UreA	urease alpha subunit	
-	GST	used for background subtraction	9

Table S2: Distributions of serum antibody responses in UK Biobank prior to normalisation

Antigen	No. samples	mean	SD	median	max	min
HSV-1 (1gG)	9695	3192.093	3004.911	2974	15718	1
HSV-2 (2mgG)	9695	272.784	868.506	32	10630	1
VZV (gE/gI)	9695	978.344	1195.509	520	11635	1
EBV (VCA p18)	9695	7289.483	3419.997	7473	19010	1
EBV (EBNA-1 pep)	9695	4425.043	3116.959	4324	16969	1
EBV (ZEBRA)	9695	2348.944	1965.866	1990	13637	1
EBV (EA-D)	9695	2558.274	2588.912	1735	15853	1
CMV (pp150 Nter)	9695	1602.94	2014.004	597	13233	1
CMV (pp52)	9695	3177.482	3411.886	2010	14979	1
CMV (pp28)	9695	1343.486	1657.125	491	11120	1
HHV-7 (U14)	9695	848.617	804.376	600	10321	1
KSHV (LANA)	9695	92.937	590.933	16	12897	1
KSHV (K8.1)	9695	73.157	122.948	57	4375	1
HHV-6 (IE1B)	9695	521.343	885.102	233	12488	1
HHV-6 (IE1A)	9695	314.626	375.556	201	5678	1
HHV-6 (p101 k)	9695	149.713	463.484	21	11715	1
HBV (HBc)	9695	71.24	610.159	5	14302	1
HBV (HBe)	9695	97.549	578.451	13	13906	1
HCV (Core)	9695	31.179	314.593	3	12542	1
HCV (NS3)	9695	53.024	347.801	31	13384	1
T. gondii (p22)	9695	74.733	194.781	31	7153	1
T. gondii (sag1)	9695	128.452	153.086	90	5787.8	1
HTLV-1 (gag)	9695	292.457	356.300	134	2717	1
HTLV-1 (env)	9695	33.871	41.442	27	2203	1
HIV (gag)	9695	126.77	362.583	55	10819	1
HIV (env)	9695	47.215	70.339	39	3972	1
BKV (VP1)	9695	3763.077	2706.281	3428	14978	1
JCV (VP1)	9695	792.817	1163.624	314	10975	1
MCV (VP1)	9695	2214.352	2449.434	1006	10506	1
HPV16 (L1)	9695	67.737	171.04	40	4983	1
HPV16 (E6)	9695	35.296	296.766	11	11781	1
HPV16 (E7)	9695	40.78	188.208	18	9305	1
HPV18 (L1)	9695	56.813	143.47	38	5876	1
C. trachomatis (mompD)	9695	138.612	478.048	16	8022	1
C. trachomatis (mompA)	9695	80.089	300.058	19	7870	1
C. trachomatis (tarp F2)	9695	201.311	578.011	25	9199	1
C. trachomatis (tarp F1)	9695	187.744	636.115	12	10384	1
C. trachomatis (PorB)	9695	23.29	57.587	13	2599	1
C. trachomatis (pGP3)	9695	607.845	1615.422	9	13749	1
H. pylori (CagA)	4871	994.417	2277.809	72	14106	1
H. pylori (VacA)	9695	178.468	651.847	28	10661	1
H. pylori (HP1564)	9695	500.069	1176.451	43	12055	1
H. pylori (GroEL)	9695	1021.471	2224.667	21	13150	1

H. pylori (Catalase)	9695	589.451	1874.24	41	13972	1
H. pylori (UreA)	9695	434.729	1422.169	34	14085	1

Table S3: Cohort characteristics for UK Biobank and CoLaus/PsyCoLaus serology data

Characteristic	UK Biobank	(%)	CoLaus/PsyCoLaus	(%)
**Age at recruitment**				
30-39	0	0.00	476	11.29
40-49	2250	23.41	1280	30.36
50-59	3145	32.72	1159	27.49
60-69	4158	43.26	991	23.51
70+	58	0.60	310	7.35
**Sex**				
Male	4236	44.07	1946	46.16
Female	5375	55.93	2270	53.84
**Reported ethnicity**				
White	9061	94.28	4216	100
Asian	234	2.43		
Black	139	1.45		
Other	134	1.39		
Not reported/missing	43	0.45		

Table S4: Phenotype definitions of ICD-10 derived phenotypes

See Excel spreadsheet

Table S5: Phenotype definitions of self-reported phenotypes

Phenotype group	Phenotype	Cases	Controls	UK Biobank code(s)
Allergy	Allergy (unspecified)	3584	369427	1374
Allergy	Asthma	57509	315502	1111
Allergy	Eczema	14021	358990	1452
Allergy	Hayfever	30537	342474	1387
Infection	Diphtheria	81	298210	1574
Infection	EBV	1035	298210	1567
Infection	*Helicobacter pylori*	1532	298210	1442
Infection	Hepatitis A	252	298210	1578
Infection	Hepatitis C	135	298210	1580
Infection	HSV	260	298210	1575
Infection	Measles	5560	298210	1568
Infection	Mumps	3384	298210	1569
Infection	Pertussis	1282	298210	1572
Infection	Polio	402	298210	1526
Infection	Rubella	1996	298210	1570
Infection	Scarlet fever	1012	298210	1677
Infection	*Staphylococcus aureus* (MRSA)	72	298210	1566
Infection	Tonsillitis	4893	298210	1598
Infection	Tuberculosis	2581	298210	1440
Infection	Typhoid fever	60	298210	1577
Infection	VZV (Chickenpox)	6886	298210	1571
Infection	VZV (Combined)	7475	298210	1571, 1573, 1674
Infection	VZV (Shingles)	1067	298210	1573
Inflammation	Coronary artery disease	2015	370977	1082
Inflammation	COPD	1895	371116	1112
Inflammation	Diabetes (type 1)	478	372533	1222
Inflammation	Multiple sclerosis	1738	371273	1261
Inflammation	Myocardial infarction	11592	361400	1075
Inflammation	Parkinson's	901	372110	1262
Inflammation	Psoriasis	5861	367150	1453
Inflammation	Rheumatoid arthritis	5713	367298	1464
Inflammation	Rosacea	1010	372001	1660
Inflammation	Sarcoidosis	1030	371981	1371
Inflammation	Systemic lupus erythematosus	636	372375	1381
Inflammation	Ulcerative colitis	2635	370357	1463
Other	Abnormal cervical smear	804	201181	1663
Other	Alzheimer's	137	372874	1263
Other	Cervical abnormality	1225	200760	1554, 1663
Other	Cervical intraepithelial neoplasia	433	201552	1554
Infection	Additional codes included in super-phenotype	NA	NA	1156, 1196, 1244, 1246, 1247, 1274, 1412, 1416, 1418, 1439, 1514, 1515, 1576, 1579, 1581, 1582, 1594, 1657, 1676, 1678

Table S6: Top variants for genomic regions excluding the extended MHC region associated with antibody responses to 45 pathogen-derived antigens. Betas are reported relative to the minor allele.

Chr	Region	Antigen	SNP	MAF	Beta	SE	P
3	68638922-69293121	*C. trachomatis* (mompA)	3:68985092_TAGTGACA_T	0.030	0.279	0.045	6.50×10^{-10}
		C. trachomatis (mompD)	3:68985092_TAGTGACA_T	0.030	0.262	0.045	7.10×10^{-9}
4	103390496-104199922	EBV (EA-D)	rs230493	0.355	-0.068	0.015	3.60×10^{-6}
		EBV (VCAp18)	rs1289231	0.077	0.126	0.028	7.00×10^{-6}
		EBV (ZEBRA)	rs3755867	0.332	-0.076	0.015	3.60×10^{-7}
		EBV (ZEBRA)	rs4699031	0.332	-0.076	0.015	3.60×10^{-7}
		HHV-6 (IE1A)	4:103406914_TA_T	0.366	-0.096	0.015	1.30×10^{-10}
		HHV-7 (U14)	rs4648052	0.375	-0.072	0.014	5.70×10^{-7}
		HIV (env)	rs567727578	0.468	-0.068	0.015	4.00×10^{-6}
		HPV18 (L1)	rs4648052	0.375	-0.068	0.015	3.90×10^{-6}
		HTLV-1 (env)	rs4648068	0.330	-0.075	0.015	1.00×10^{-6}
		HTLV-1 (env)	rs4648058	0.330	-0.075	0.015	1.00×10^{-6}
		KSHV (K8.1)	rs10013613	0.403	-0.074	0.014	2.50×10^{-7}
		KSHV (K8.1)	rs74462352	0.397	-0.075	0.014	2.50×10^{-7}
		T. gondii (sag1)	rs10013613	0.403	-0.067	0.014	3.20×10^{-6}
5	138114233-139053851	MCV (VP1)	rs13181561	0.265	0.155	0.016	4.90×10^{-22}
7	150011299-150484264	HTLV-1 (gag)	rs12534190	0.230	0.166	0.017	9.90×10^{-23}
11	118762073-118811315	HHV-7 (U14)	rs75438046	0.028	-0.278	0.043	9.80×10^{-11}
14	106624768-107228049	HCV (NS3)	rs75801933	0.066	0.145	0.029	6.80×10^{-7}
		HIV (env)	rs4977158	0.084	0.163	0.036	4.80×10^{-6}
		HIV (gag)	rs10150853	0.099	0.140	0.030	2.30×10^{-6}
		HIV (gag)	rs10138691	0.099	0.140	0.030	2.30×10^{-6}
		HPV16 (E6)	rs2337939	0.067	0.191	0.030	1.60×10^{-10}
		HPV16 (E7)	rs199944736	0.150	-0.113	0.021	5.90×10^{-8}
		HPV16 (E7)	rs201829121	0.150	-0.113	0.021	$5.90\times10\text{-}^{8}$
		HPV16 (L1)	rs4774155	0.070	0.136	0.028	1.60×10^{-6}
		HPV18 (L1)	rs75801933	0.066	0.134	0.029	4.10×10^{-6}
		HTLV-1 (env)	rs75801933	0.066	0.154	0.029	1.40×10^{-7}
		T. gondii (p22)	rs4977158	0.084	0.176	0.036	7.70×10^{-7}
		T. gondii (sag1)	rs4977158	0.084	0.213	0.035	1.70×10^{-9}

19	49097126-49282803	BKV (VP1)	rs681343	0.489	-0.123	0.014	3.80×10^{-18}
		JCV (VP1)	rs681343	0.489	-0.134	0.014	1.70×10^{-21}
22	23154058-23220565	*H. pylori* (UreA)	22:23169419_GT_G	0.173	-0.119	0.019	9.00×10^{-10}
22	41424903-42698602	HHV-7 (U14)	rs35211694	0.245	0.117	0.019	4.40×10^{-10}

Table S7: Top variants within the extended MHC region (chr6:25,384,361-34,366,455) associated with antibody responses to 45 pathogen-derived antigens. Betas are reported relative to the minor allele.

**Antigen**	**SNP**	**MAF**	**Beta**	**SE**	**P**
BKV (VP1)	rs4713573	0.324	0.079	0.016	4.70×10^{-7}
CMV (pp150)	rs9275015	0.300	-0.086	0.017	2.30×10^{-7}
CMV (pp52)	rs9270798	0.232	0.096	0.017	1.00×10^{-8}
C. trachomatis (mompA)	rs71536538	0.273	-0.081	0.017	3.50×10^{-6}
C. trachomatis (mompD)	rs4367411	0.201	-0.098	0.019	1.80×10^{-7}
C. trachomatis (TarpDF2)	rs112438356	0.098	-0.139	0.026	5.00×10^{-8}
C. trachomatis (TarpDF2)	rs112327275	0.098	-0.139	0.026	5.00×10^{-8}
EBV (EA-D)	rs2395192	0.460	-0.163	0.014	3.40×10^{-31}
EBV (EBNA1)	rs530423766	0.433	-0.324	0.015	6.90×10^{-97}
EBV (VCAp18)	rs9264759	0.208	0.158	0.018	4.00×10^{-19}
EBV (ZEBRA)	rs9274704	0.278	0.306	0.016	2.70×10^{-86}
HBV (HBc)	rs112275579	0.365	0.079	0.017	3.80×10^{-6}
HBV (HBe)	rs9266092	0.391	0.070	0.015	4.90×10^{-6}
HCV (NS3)	rs3997848	0.323	0.082	0.015	1.20×10^{-7}
HHV-6 (IE1A)	rs9265967	0.139	-0.134	0.021	2.30×10^{-10}
HHV-6 (IE1B)	rs28383304	0.145	0.146	0.021	1.30×10^{-12}
HHV-6 (p101k)	rs4959072	0.219	0.120	0.017	5.40×10^{-12}
HHV-7 (U14)	rs9270140	0.143	-0.163	0.021	6.20×10^{-15}
HIV (env)	rs41288885	0.059	0.153	0.031	1.20×10^{-6}
HIV (env)	6:32748889_CT_C	0.046	0.185	0.038	1.20×10^{-6}
HIV (gag)	rs3134974	0.445	-0.072	0.015	2.10×10^{-6}
HPV16 (E6)	rs35445446	0.168	0.087	0.019	6.60×10^{-6}
HPV16 (E7)	rs78274956	0.495	-0.130	0.016	1.20×10^{-16}
HPV16 (L1)	rs548129848	0.417	0.088	0.017	1.00×10^{-7}
HPV18 (L1)	rs6930081	0.468	-0.066	0.015	5.60×10^{-6}
HPV18 (L1)	rs6929819	0.468	-0.066	0.014	5.60×10^{-6}
HSV-1 (1gG)	rs116135626	0.187	0.151	0.025	1.20×10^{-9}
HSV-2 (2mgG)	rs36152293	0.337	0.107	0.018	4.60×10^{-9}
HTLV-1 (env)	rs115675626	0.088	-0.154	0.027	2.20×10^{-8}
HTLV-1 (gag	rs9271727	0.252	0.164	0.017	2.20×10^{-22}
H. pylori (CagA)	rs71534592	0.121	-0.172	0.032	5.60×10^{-8}
H. pylori (Catalase)	rs9270641	0.199	0.104	0.020	3.90×10^{-7}
H. pylori (HP1564)	rs9268541	0.058	0.146	0.030	1.30×10^{-6}
H. pylori (VacA)	rs35186928	0.370	-0.075	0.015	3.30×10^{-7}
JCV (VP1)	rs71538510	0.173	-0.323	0.019	8.50×10^{-66}
KSHV (K8.1)	rs2844535	0.274	-0.076	0.016	1.80×10^{-6}
MCV (VP1)	rs9269771	0.274	-0.249	0.017	2.70×10^{-48}
T. gondii (sag1)	rs144947706	0.369	-0.113	0.016	1.40×10^{-12}
VZV (gE/gI)	rs9273325	0.174	0.221	0.019	7.10×10^{-33}

Table S8: Top variants associated with antibody response in the UK Biobank and CoLaus datasets following meta-analysis

		UK Biobank			CoLaus			Meta-analysis (fixed-effects)		
**Antigen**	**SNP ID (UKB)**	**beta**	**SE**	**P**	**beta**	**SE**	**P**	**beta**	**SE**	**P**
EBV (EAD)	rs2395192	-0.163	0.014	3.40×10^{-31}	-0.111	0.027	4.83×10^{-5}	-0.152	0.012	3.54×10^{-34}
EBV (ZEBRA)	rs9274728	-0.306	0.016	3.70×10^{-86}	-0.323	0.032	1.83×10^{-24}	-0.309	0.014	8.74×10^{-109}
HHV6 (IE1A)	rs8192589	0.126	0.020	4.70×10^{-10}	0.092	0.037	1.41×10^{-2}	0.118	0.018	2.98×10^{-11}
HHV7 (U14)	6:32593225_CAG_C	0.198	0.026	7.20×10^{-14}	0.218	0.044	5.77×10^{-7}	0.203	0.023	2.44×10^{-19}
KSHV (K8.1)	rs2372093	-0.080	0.018	1.20×10^{-5}	-0.082	0.029	5.46×10^{-3}	-0.080	0.016	2.10×10^{-7}
T. gondii (sag1)	rs7754119	0.103	0.015	1.10×10^{-11}	0.053	0.023	2.04×10^{-2}	0.088	0.013	3.95×10^{-12}

Table S9: Published associations between *NFKB1* and disease-related traits

Published trait	Trait type	Ancestry	SNP	R^2 (rs28362491)	Study size (cases)	P	OR (del)	95% LCI	95% UCI	Ref
Allergic rhinitis	Allergy	European	rs12509403	0.556	891,367 (120,482)	1.17×10^{-15}	0.960	0.950	0.97	13
Allergic sensitisation	Allergy	European	rs4648050	0.572	24,481 (8,040)	2.03×10^{-8}	0.880	0.840	0.92	13
Combined hayfever and eczema	Allergy	White British	rs230507	0.776	346,545 (106,772)	1.04×10^{-9}	0.963	0.951	0.974	14
Tonsillectomy	Infection	European	rs230523	0.759	173,421 (60,098)	4.54×10^{-14}	1.070	1.060	1.08	15
ARDS* (<65yrs)	Inflammation	European	rs28362491	NA	NR	3.00×10^{-2}	0.106	0.014	0.825	16
Ankylosing spondylitis	Inflammation	European	rs3774937	0.751	86,475 (52,262)	7.83×10^{-10}	1.120	1.080	1.16	17
Behçet's disease	Inflammation	Turkish	rs28362491	NA	270 (89)	4.00×10^{-3}	0.556	0.380	0.811	18
Coronary artery disease	Inflammation	European	rs28362491	NA	220 (120)	1.50×10^{-2}	2.880	1.210	6.84	19
Coronary artery disease	Inflammation	India	rs28362491	NA	830 (600)	2.90×10^{-2}	1.260	1.030	1.55	20
Coronary artery disease	Inflammation	Uygur	rs28362491	NA	2020 (960)	<0.001	1.581	1.222	2.046	21
Left ventricular dysfunction in CAD*	Inflammation	India	rs28362491	NA	600 (190)	7.00×10^{-3}	2.340	1.310	4.17	20
Lung injury score in ARDS*	Inflammation	European	rs28362491	NA	103 (77)	3.00×10^{-4}	3.700	1.800	7.9	22
Mouth Ulcers	Inflammation	European	rs4699030	0.863	816,850 (145,377)	5.91×10^{-11}	1.030	1.020	1.04	23
Myocardial infarction	Inflammation	European	rs28362491	NA	253 (86)	<0.001	0.304	0.177	0.522	24
Primary biliary cholangitis	Inflammation	Japanese	rs230534	0.745	2,886 (1,381)	1.50×10^{-7}	1.340	1.200	1.49	25
Primary sclerosing cholangitis	Inflammation	European	rs3774937	0.751	37,621 (3,408)	6.11×10^{-9}	1.167	1.107	1.229	17
Systemic sclerosis	Inflammation	Iran/Turkey	rs4648133	0.572	2107 (764)	3.11×10^{-7}	1.470	1.270	1.7	26
TR schizophrenia*	Inflammation	Han Chinese	rs230529	0.897	1,610 (804)	1.74×10^{-7}	1.450	1.260	1.66	27
Ulcerative colitis	Inflammation	European	rs3774959	0.698	47,560 (17,865)	3.66×10^{-12}	1.118	1.077	1.159	28
Ulcerative colitis	Inflammation	European	rs3774937	0.751	48,626 (14,413)	1.33×10^{-11}	1.107	1.075	1.142	17
Ulcerative colitis	Inflammation	European	rs28362491	NA	1,152 (350)	4.30×10^{-3}	1.570	1.140	2.16	29
Ulcerative colitis	Inflammation	European	rs28362491	NA	282 (127)	1.10×10^{-2}	2.510	1.280	4.92	30
Cancer risk	Other	European/ Chinese	rs28362491	NA	43,000 (18222)	2.00×10^{-3}	0.890	0.830	0.96	31
**Published trait**		**Ancestry**	**SNP**	**R^2**	**Study size (cases)**	**P**	**Beta (del)**	**SE**		**Ref**
Albumin/Globulin Ratio	Other	Japanese	rs60371688	0.850	98,626	1.49×10^{-12}	0.036	0.005		32
Asthma	Allergy	White British	rs59123962	0.697	458,699	7.50×10^{-4}	-0.002	0.001		33
Combined hayfever, allergic rhinitis, and eczema	Allergy	White British	rs230504	0.777	458,699	6.90×10^{-13}	-0.007	0.001		33
Non-albumin protein	Other	Japanese	rs1585213	0.949	98,538	1.47×10^{-15}	-0.037	0.005		32
Immunogobulin levels ((A + G)/M)	Other	European	rs4648052	0.700	19,219	3.00×10^{-7}	-0.060	NR		34
Immunogobulin levels (A - M)	Other	European	rs4648052	0.700	19,219	2.30×10^{-7}	-0.070	NR		34

*ARDS: Acute respiratory distress syndrome; CAD: Coronary artery disease; TR: Treatment resistant

Table S10: Association statistics for rs28362491 and self-reported and ICD-10 derived phenotypes after exclusion of phenotypes where the regression model was poorly fitted

Phenotype group	Phenotype	OR	95% CI	P
Allergy	Allergic dermatitis (ICD)	0.957	0.747-1.227	0.729
Allergy	Allergy (unspecified) (SR)	0.986	0.940-1.033	0.547
Allergy	Asthma	0.993	0.982-1.005	0.280
Allergy	Atopic dermatitis (ICD)	0.850	0.637-1.133	0.267
Allergy	Eczema (SR)	0.981	0.957-1.005	0.123
Allergy	Hayfever	0.965	0.949-0.981	3.57×10^{-5}
Allergy	Seborrhoeic dermatitis (ICD)	0.817	0.601-1.111	0.197
Infection	*Aspergillus spp.* (ICD)	0.946	0.786-1.140	0.560
Infection	*Campylobacter spp.* (ICD)	1.001	0.885-1.132	0.986
Infection	*Candida spp.* (ICD)	0.993	0.947-1.042	0.778
Infection	*Clostridium difficile* (ICD)	1.018	0.930-1.115	0.697
Infection	CMV (ICD)	1.094	0.890-1.345	0.395
Infection	*Corynebacterium diphtheriae*	0.912	0.667-1.248	0.566
Infection	EBV	1.067	0.979-1.162	0.143
Infection	*Enterobius vermicularis* (ICD)	1.110	0.868-1.420	0.406
Infection	Enterovirus (ICD)	0.989	0.698-1.399	0.948
Infection	*Escherichia coli* (ICD)	1.026	0.984-1.071	0.230
Infection	*Giardia lamblia* (ICD)	1.026	0.708-1.485	0.894
Infection	*Haemophilus influenzae* (ICD)	1.012	0.896-1.143	0.850
Infection	*Helicobacter pylori*	1.022	0.974-1.073	0.371
Infection	Hepatitis A	1.027	0.882-1.195	0.734
Infection	Hepatitis C	1.122	0.983-1.281	0.087
Infection	HSV	1.032	0.926-1.149	0.570
Infection	Influenza (ICD)	1.120	1.000-1.253	4.90×10^{-2}
Infection	*Klebsiella pneumoniae* (ICD)	1.053	0.947-1.172	0.339
Infection	*Legionella spp*. (ICD)	1.524	1.097-2.118	1.21×10^{-2}
Infection	Measles	0.978	0.941-1.016	0.247
Infection	MOCV (ICD)	1.056	0.778-1.434	0.726
Infection	Mumps	0.996	0.948-1.045	0.862
Infection	*Neisseria meningitidis* (ICD)	0.704	0.509-0.974	3.43×10^{-2}
Infection	Norovirus (ICD)	1.208	0.966-1.511	0.098
Infection	Papillomavirus (ICD)	1.095	1.019-1.177	1.38×10^{-2}
Infection	Pertussis	0.984	0.909-1.065	0.693
Infection	*Pneumocystis jirovecii* (ICD)	1.007	0.735-1.381	0.963
Infection	Polio	0.958	0.835-1.100	0.544
Infection	*Proteus spp*. (ICD)	1.143	0.996-1.311	0.057
Infection	*Staphylococcus aureus*	1.012	0.964-1.062	0.635
Infection	Streptococcus A	1.072	0.991-1.160	0.082
Infection	Streptococcus B (ICD)	1.063	0.924-1.222	0.391
Infection	Streptococcus D (ICD)	0.966	0.792-1.177	0.729
Infection	*Streptococcus pneumoniae* (ICD)	1.068	0.956-1.193	0.246
Infection	Tonsillitis (SR)	0.976	0.937-1.016	0.235
Infection	Tuberculosis (SR)	0.984	0.931-1.041	0.576
Infection	VZV (Chicken pox)	0.983	0.950-1.017	0.313
Infection	VZV (Combined)	0.994	0.963-1.026	0.704
Infection	VZV (Shingles)	1.028	0.961-1.099	0.421
Inflammation (Other)	Coronary artery disease	1.004	0.989-1.019	0.595

Inflammation (Other)	COPD	1.016	0.991-1.042	0.206
Inflammation (Other)	Encephalomyelitis (ICD)	1.048	0.874-1.258	0.611
Inflammation (Other)	Inflammatory polyarthropathy (ICD)	0.864	0.687-1.087	0.213
Inflammation (Other)	Motor neuron disease (ICD)	1.062	0.913-1.236	0.435
Inflammation (Chronic)	Multiple Sclerosis	1.047	0.985-1.113	0.144
Inflammation (Other)	Myocardial infarction	1.006	0.985-1.028	0.550
Inflammation (Other)	Parkinson's	1.044	0.981-1.112	0.177
Inflammation (Chronic)	Psoriasis	1.044	1.005-1.083	2.55×10^{-2}
Inflammation (Chronic)	Rheumatoid Arthritis	0.986	0.955-1.018	0.389
Inflammation (Other)	Rosacea	0.979	0.908-1.055	0.577
Inflammation (Other)	Ulcerative colitis	1.079	1.037-1.124	2.15×10^{-4}
Other	Abnormal cervical smear (SR)	0.990	0.896-1.093	0.836
Other	Alzheimer's	1.109	1.012-1.215	2.65×10^{-2}
Other	Cervical abnormality (SR)	1.055	0.973-1.144	0.196
Other	Cervical intraepithelial neoplasia (SR)	1.166	1.020-1.334	2.48×10^{-2}
	Allergy	0.988	0.978-0.998	1.84×10^{-2}
	Chronic inflammation	1.020	0.998-1.042	0.075
	Infection	1.014	1.001-1.027	3.31×10^{-2}

Table S11: Association statistics for rs28362491 and mortality associated with allergy, infection, or inflammation

Phenotype	Cases	Controls	OR	95% CI	P
Allergy	131	25205	0.846	0.658-1.088	0.192
Infection	1244	24092	1.065	0.982-1.155	0.129
Inflammation	4421	20915	1.038	0.991-1.087	0.117

Table S12: Association statistics for rs28362491 and blood cell counts

Phenotype	Beta	SE	P
Leukocyte count	-9.50×10^{-5}	1.97×10^{-3}	0.960
Lymphocyte count	1.98×10^{-2}	1.95×10^{-3}	3.30×10^{-24}
Basophil count	-1.25×10^{-2}	2.08×10^{-3}	2.00×10^{-9}
Eosinophil count	6.49×10^{-3}	1.96×10^{-3}	9.30×10^{-4}
Monocyte count	-1.50×10^{-2}	1.89×10^{-3}	2.70×10^{-15}
Neutrophil count	-7.27×10^{-3}	1.99×10^{-3}	2.50×10^{-4}
RBC count	-1.04×10^{-2}	1.67×10^{-3}	4.80×10^{-10}
Platelet count	-5.79×10^{-3}	1.79×10^{-3}	1.30×10^{-3}

Table S13: Colocalisation results for GWAS and eQTL phenotype pairs

Phenotype 1	Phenotype 2	Lead SNP 1	Lead SNP 2	PP H0	PP H1	PP H2	PP H3	PP H4
EBV (EA-D)	EBV (ZEBRA)	rs230493	rs4648058	2.02×10^{-4}	3.31×10^{-3}	1.01×10^{-2}	1.64×10^{-1}	8.22×10^{-1}
EBV (ZEBRA)	HHV-6 (IE1A)	rs4648058	rs1598859	1.28×10^{-8}	6.42×10^{-7}	2.88×10^{-3}	1.43×10^{-1}	8.54×10^{-1}
HHV-6 (IE1A)	HHV-7 (U14)	rs1598859	rs4648052	9.53×10^{-8}	2.15×10^{-2}	1.82×10^{-6}	4.09×10^{-1}	5.69×10^{-1}
HHV-7 (U14)	HIV (Env)	rs4648052	rs10013613	4.71×10^{-4}	9.01×10^{-3}	6.80×10^{-3}	1.28×10^{-1}	8.55×10^{-1}
HIV (Env)	HPV18 (L1)	rs10013613	rs4648052	9.18×10^{-4}	1.33×10^{-2}	9.37×10^{-3}	1.34×10^{-1}	8.43×10^{-1}
HPV18 (L1)	HTLV-1 (Env)	rs4648052	rs4648058	5.38×10^{-4}	5.49×10^{-3}	1.31×10^{-2}	1.32×10^{-1}	8.49×10^{-1}
HTLV-1 (Env)	KSHV (K8.1)	rs4648058	rs10013613	7.84×10^{-5}	1.91×10^{-3}	9.39×10^{-3}	2.27×10^{-1}	7.61×10^{-1}
KSHV (K8.1)	*T. gondii* (sag1)	rs10013613	rs10013613	7.81×10^{-5}	9.36×10^{-3}	8.16×10^{-4}	9.59×10^{-2}	8.94×10^{-1}
HHV-6 (IE1A)	Basophil count	rs1598859	rs2168805	1.33×10^{-10}	2.99×10^{-5}	1.70×10^{-6}	3.81×10^{-1}	6.19×10^{-1}
HHV-6 (IE1A)	Eosinophil count	rs1598859	rs230507	3.71×10^{-21}	8.35×10^{-16}	6.10×10^{-7}	1.36×10^{-1}	8.64×10^{-1}
HHV-6 (IE1A)	Lymphocyte count	rs1598859	rs35680095	7.00×10^{-18}	1.58×10^{-12}	4.44×10^{-6}	9.99×10^{-1}	1.45×10^{-7}
HHV-6 (IE1A)	Monocyte count	rs1598859	rs980455	4.04×10^{-17}	9.09×10^{-12}	1.33×10^{-6}	2.97×10^{-1}	7.03×10^{-1}
HHV-6 (IE1A)	Red blood cell count	rs1598859	rs230539	3.15×10^{-12}	7.08×10^{-7}	9.36×10^{-7}	2.09×10^{-1}	7.91×10^{-1}
HHV-6 (IE1A)	Platelet count	rs1598859	rs62328536	2.49×10^{-6}	5.60×10^{-1}	4.75×10^{-7}	1.06×10^{-1}	3.33×10^{-1}
HHV-6 (IE1A)	eQTL: Monocytes (unstim)	rs1598859	rs72696119	5.34×10^{-7}	1.20×10^{-1}	1.26×10^{-6}	2.82×10^{-1}	5.98×10^{-1}
HHV-6 (IE1A)	eQTL: Monocytes (LPS2)	rs1598859	rs4698856	1.39×10^{-8}	3.14×10^{-3}	1.53×10^{-6}	3.43×10^{-1}	6.53×10^{-1}
HHV-6 (IE1A)	eQTL: Monocytes (LPS24)	rs1598859	rs28882677	1.60×10^{-8}	3.61×10^{-3}	2.10×10^{-6}	4.72×10^{-1}	5.25×10^{-1}
HHV-6 (IE1A)	eQTL: Monocytes (IFN)	rs1598859	rs4648055	9.37×10^{-12}	2.11×10^{-6}	6.76×10^{-7}	1.50×10^{-1}	8.50×10^{-1}
HHV-6 (IE1A)	eQTL: Neutrophils	rs1598859	rs4698857	6.31×10^{-8}	1.42×10^{-2}	7.72×10^{-7}	1.72×10^{-1}	8.14×10^{-1}
HHV-6 (IE1A)	eQTL: Natural killer cells	rs1598859	rs72696119	3.90×10^{-8}	8.78×10^{-3}	1.18×10^{-6}	2.63×10^{-1}	7.28×10^{-1}
Monocyte count	eQTL: Monocytes (unstim)	rs980455	rs72696119	1.44×10^{-12}	4.73×10^{-2}	3.40×10^{-12}	1.10×10^{-1}	8.43×10^{-1}
Monocyte count	eQTL: Monocytes (LPS2)	rs980455	rs4698856	2.75×10^{-14}	9.03×10^{-4}	3.02×10^{-12}	9.74×10^{-2}	9.02×10^{-1}
Monocyte count	eQTL: Monocytes (LPS24)	rs980455	rs28882677	2.15×10^{-14}	7.05×10^{-4}	2.81×10^{-12}	9.05×10^{-2}	9.09×10^{-1}
Monocyte count	eQTL: Monocytes (IFN)	rs980455	rs4648055	1.44×10^{-16}	4.73×10^{-6}	1.04×10^{-11}	3.40×10^{-1}	6.60×10^{-1}
Neutrophil count	eQTL: Neutrophils	rs2903281	rs4698857	2.33×10^{-3}	3.22×10^{-2}	2.84×10^{-2}	3.93×10^{-1}	5.44×10^{-1}
Lymphocyte count	eQTL: Natural killer cells	rs35680095	rs72696119	5.05×10^{-14}	3.20×10^{-2}	1.52×10^{-12}	9.65×10^{-1}	2.78×10^{-3}

*PP: Posterior probability

Table S14: Two-sample Mendelian Randomisations results for causal effect of blood cell counts on antibody response

Exposure	Outcome	Coef.Estimate	SE	P
RBC count	EBV (EAD)	5.696	1.370	3.21×10^{-5}
RBC count	EBV (ZEBRA)	5.955	1.364	1.27×10^{-5}
RBC count	HHV6 (IE1A)	8.292	1.397	2.92×10^{-9}
RBC count	HHV-7 (U14)	5.526	1.362	4.98×10^{-5}
RBC count	HIV (env)	5.905	1.398	2.39×10^{-5}
RBC count	HPV-18 (L1)	5.706	1.395	4.32×10^{-5}
RBC count	HTLV-1 (env)	5.660	1.396	5.05×10^{-5}
RBC count	KSHV (K8.1)	6.676	1.386	1.46×10^{-6}
RBC count	*T. gondii* (sag1)	6.065	1.384	1.17×10^{-5}
Basophil count	EBV (EAD)	4.742	1.140	3.21×10^{-5}
Basophil count	EBV (ZEBRA)	4.958	1.136	1.27×10^{-5}
Basophil count	HHV6 (IE1A)	6.903	1.163	2.92×10^{-9}
Basophil count	HHV-7 (U14)	4.601	1.134	4.98×10^{-5}
Basophil count	HIV (env)	4.916	1.164	2.39×10^{-5}
Basophil count	HPV-18 (L1)	4.750	1.162	4.32×10^{-5}
Basophil count	HTLV-1 (env)	4.712	1.163	5.05×10^{-5}
Basophil count	KSHV (K8.1)	5.558	1.154	1.46×10^{-6}
Basophil count	*T. gondii* (sag1)	5.049	1.152	1.17×10^{-5}
Neutrophil count	EBV (EAD)	8.144	1.958	3.21×10^{-5}
Neutrophil count	EBV (ZEBRA)	8.515	1.950	1.27×10^{-5}
Neutrophil count	HHV6 (IE1A)	11.856	1.997	2.92×10^{-9}
Neutrophil count	HHV-7 (U14)	7.902	1.948	4.98×10^{-5}
Neutrophil count	HIV (env)	8.444	1.998	2.39×10^{-5}
Neutrophil count	HPV-18 (L1)	8.159	1.995	4.32×10^{-5}
Neutrophil count	HTLV-1 (env)	8.093	1.997	5.05×10^{-5}
Neutrophil count	KSHV (K8.1)	9.545	1.982	1.46×10^{-6}
Neutrophil count	*T. gondii* (sag1)	8.671	1.978	1.17×10^{-5}
Monocyte count	EBV (EAD)	3.957	0.952	3.21×10^{-5}
Monocyte count	EBV (ZEBRA)	4.137	0.948	1.27×10^{-5}
Monocyte count	HHV6 (IE1A)	5.761	0.970	2.92×10^{-9}
Monocyte count	HHV-7 (U14)	3.840	0.947	4.98×10^{-5}
Monocyte count	HIV (env)	4.103	0.971	2.39×10^{-5}
Monocyte count	HPV-18 (L1)	3.964	0.969	4.32×10^{-5}
Monocyte count	HTLV-1 (env)	3.932	0.970	5.05×10^{-5}
Monocyte count	KSHV (K8.1)	4.638	0.963	1.46×10^{-6}
Monocyte count	*T. gondii* (sag1)	4.214	0.961	1.17×10^{-5}
Eosinophil count	EBV (EAD)	-9.118	2.193	3.21×10^{-5}
Eosinophil count	EBV (ZEBRA)	-9.533	2.184	1.27×10^{-5}
Eosinophil count	HHV6 (IE1A)	-13.274	2.236	2.92×10^{-9}
Eosinophil count	HHV-7 (U14)	-8.847	2.181	4.98×10^{-5}
Eosinophil count	HIV (env)	-9.453	2.237	2.39×10^{-5}
Eosinophil count	HPV-18 (L1)	-9.134	2.233	4.32×10^{-5}
Eosinophil count	HTLV-1 (env)	-9.061	2.236	5.05×10^{-5}
Eosinophil count	KSHV (K8.1)	-10.687	2.219	1.46×10^{-6}

Eosinophil count	*T. gondii* (sag1)	-9.709	2.215	1.17×10^{-5}
Lymphocyte count	EBV (EAD)	-2.988	0.719	3.21×10^{-5}
Lymphocyte count	EBV (ZEBRA)	-3.124	0.716	1.27×10^{-5}
Lymphocyte count	HHV6 (IE1A)	-4.350	0.733	2.92×10^{-9}
Lymphocyte count	HHV-7 (U14)	-2.899	0.715	4.98×10^{-5}
Lymphocyte count	HIV (env)	-3.098	0.733	2.39×10^{-5}
Lymphocyte count	HPV-18 (L1)	-2.993	0.732	4.32×10^{-5}
Lymphocyte count	HTLV-1 (env)	-2.969	0.733	5.05×10^{-5}
Lymphocyte count	KSHV (K8.1)	-3.502	0.727	1.46×10^{-6}
Lymphocyte count	*T. gondii* (sag1)	-3.181	0.726	1.17×10^{-5}
Platelet count	EBV (EAD)	10.227	2.459	3.21×10^{-5}
Platelet count	EBV (ZEBRA)	10.693	2.449	1.27×10^{-5}
Platelet count	HHV6 (IE1A)	14.889	2.508	2.92×10^{-9}
Platelet count	HHV-7 (U14)	9.923	2.446	4.98×10^{-5}
Platelet count	HIV (env)	10.604	2.510	2.39×10^{-5}
Platelet count	HPV-18 (L1)	10.246	2.505	4.32×10^{-5}
Platelet count	HTLV-1 (env)	10.163	2.507	5.05×10^{-5}
Platelet count	KSHV (K8.1)	11.987	2.489	1.46×10^{-6}
Platelet count	*T. gondii* (sag1)	10.890	2.485	1.17×10^{-5}

Table S15: Power calculations for Mendelian Randomisation analyses for causal effect of blood cell counts on antibody response

Exposure	Outcome	Power for full cohort
RBC count	EBV (EAD)	99.90%
RBC count	EBV (ZEBRA)	100.00%
RBC count	HHV6 (IE1A)	100.00%
RBC count	HHV-7 (U14)	99.80%
RBC count	HIV (env)	99.90%
RBC count	HPV-18 (L1)	99.90%
RBC count	HTLV-1 (env)	99.90%
RBC count	KSHV (K8.1)	100.00%
RBC count	*T. gondii* (sag1)	100.00%
Basophil count	EBV (EAD)	98.20%
Basophil count	EBV (ZEBRA)	98.90%
Basophil count	HHV6 (IE1A)	100.00%
Basophil count	HHV-7 (U14)	97.60%
Basophil count	HIV (env)	98.70%
Basophil count	HPV-18 (L1)	98.70%
Basophil count	HTLV-1 (env)	98.10%
Basophil count	KSHV (K8.1)	99.70%
Basophil count	*T. gondii* (sag1)	99.70%
Neutrophil count	EBV (EAD)	98.80%
Neutrophil count	EBV (ZEBRA)	99.30%
Neutrophil count	HHV6 (IE1A)	100.00%
Neutrophil count	HHV-7 (U14)	98.40%
Neutrophil count	HIV (env)	99.20%
Neutrophil count	HPV-18 (L1)	98.80%
Neutrophil count	HTLV-1 (env)	98.70%
Neutrophil count	KSHV (K8.1)	99.90%
Neutrophil count	*T. gondii* (sag1)	99.40%
Monocyte count	EBV (EAD)	99.40%
Monocyte count	EBV (ZEBRA)	99.70%
Monocyte count	HHV6 (IE1A)	100.00%
Monocyte count	HHV-7 (U14)	99.10%
Monocyte count	HIV (env)	99.60%
Monocyte count	HPV-18 (L1)	99.40%
Monocyte count	HTLV-1 (env)	99.30%
Monocyte count	KSHV (K8.1)	99.90%
Monocyte count	*T. gondii* (sag1)	99.70%
Eosinophil count	EBV (EAD)	99.00%
Eosinophil count	EBV (ZEBRA)	99.40%
Eosinophil count	HHV6 (IE1A)	100.00%
Eosinophil count	HHV-7 (U14)	98.60%
Eosinophil count	HIV (env)	99.40%
Eosinophil count	HPV-18 (L1)	99.00%
Eosinophil count	HTLV-1 (env)	98.90%
Eosinophil count	KSHV (K8.1)	99.90%

Eosinophil count	*T. gondii* (sag1)	99.50%
Lymphocyte count	EBV (EAD)	99.10%
Lymphocyte count	EBV (ZEBRA)	99.50%
Lymphocyte count	HHV6 (IE1A)	100.00%
Lymphocyte count	HHV-7 (U14)	98.70%
Lymphocyte count	HIV (env)	99.40%
Lymphocyte count	HPV-18 (L1)	99.10%
Lymphocyte count	HTLV-1 (env)	99.00%
Lymphocyte count	KSHV (K8.1)	99.90%
Lymphocyte count	*T. gondii* (sag1)	99.60%
Platelet count	EBV (EAD)	99.70%
Platelet count	EBV (ZEBRA)	99.80%
Platelet count	HHV6 (IE1A)	100.00%
Platelet count	HHV-7 (U14)	99.50%
Platelet count	HIV (env)	99.80%
Platelet count	HPV-18 (L1)	99.70%
Platelet count	HTLV-1 (env)	99.70%
Platelet count	KSHV (K8.1)	100.00%
Platelet count	*T. gondii* (sag1)	99.90%

Table S16: One-sample Mendelian Randomisations results for causal effect of blood cell counts on infection and inflammation-related disease

**Exposure**	**Outcome**	**Coef.Estimate**	**SE**	**P**
RBC count	Influenza (ICD)	2.19×10^{-3}	7.227	1.000
RBC count	*Legionella spp*. (ICD)	1.64×10^{-3}	1.289	0.999
RBC count	*Neisseria meningitidis* (ICD)	7.43×10^{-4}	0.401	0.999
RBC count	Papillomavirus (ICD)	2.09×10^{-3}	1.047	0.998
RBC count	Cervical intraepithelial neoplasia (SR)	-8.29×10^{-3}	1.079	0.994
RBC count	Alzheimer's	1.23×10^{-3}	2.051	1.000
RBC count	Hayfever	1.57×10^{-3}	1.250	0.999
RBC count	Psoriasis	2.65×10^{-3}	13.594	1.000
RBC count	Ulcerative colitis	9.41×10^{-4}	0.646	0.999
RBC count	Allergy	3.03×10^{-2}	0.430	0.944
RBC count	Infection	-4.27×10^{-4}	0.580	0.999
Basophil count	Influenza (ICD)	2.38×10^{-3}	0.819	0.998
Basophil count	*Legionella spp*. (ICD)	1.61×10^{-3}	1.019	0.999
Basophil count	*Neisseria meningitidis* (ICD)	-1.04×10^{-3}	0.543	0.998
Basophil count	Papillomavirus (ICD)	2.81×10^{-3}	0.875	0.997
Basophil count	Cervical intraepithelial neoplasia (SR)	7.32×10^{-3}	4.333	0.999
Basophil count	Alzheimer's	1.94×10^{-3}	0.582	0.997
Basophil count	Hayfever	1.88×10^{-3}	0.507	0.997
Basophil count	Psoriasis	1.89×10^{-3}	0.720	0.998
Basophil count	Ulcerative colitis	1.52×10^{-3}	5.368	1.000
Basophil count	Allergy	1.86×10^{-2}	0.341	0.957
Basophil count	Infection	7.54×10^{-3}	0.509	0.988
Neutrophil count	Influenza (ICD)	2.25×10^{-3}	7.354	1.000
Neutrophil count	*Legionella spp*. (ICD)	3.56×10^{-3}	23.505	1.000
Neutrophil count	*Neisseria meningitidis* (ICD)	1.85×10^{-3}	0.781	0.998
Neutrophil count	Papillomavirus (ICD)	2.73×10^{-3}	0.721	0.997
Neutrophil count	Cervical intraepithelial neoplasia (SR)	-1.03×10^{-3}	0.552	0.999
Neutrophil count	Alzheimer's	2.49×10^{-3}	4.994	1.000
Neutrophil count	Hayfever	1.40×10^{-3}	3.022	1.000
Neutrophil count	Psoriasis	2.02×10^{-3}	1.162	0.999
Neutrophil count	Ulcerative colitis	1.68×10^{-3}	18.612	1.000
Neutrophil count	Allergy	2.81×10^{-2}	3.910	0.994
Neutrophil count	Infection	8.18×10^{-3}	0.846	0.992
Monocyte count	Influenza (ICD)	1.62×10^{-3}	1.022	0.999
Monocyte count	*Legionella spp*. (ICD)	2.07×10^{-3}	32.528	1.000
Monocyte count	*Neisseria meningitidis* (ICD)	1.35×10^{-3}	0.575	0.998
Monocyte count	Papillomavirus (ICD)	2.89×10^{-3}	1.182	0.998
Monocyte count	Cervical intraepithelial neoplasia (SR)	1.14×10^{-3}	0.616	0.999
Monocyte count	Alzheimer's	2.49×10^{-3}	1.568	0.999
Monocyte count	Hayfever	1.99×10^{-3}	6.541	1.000
Monocyte count	Psoriasis	1.96×10^{-3}	0.768	0.998
Monocyte count	Ulcerative colitis	1.34×10^{-3}	1.337	0.999
Monocyte count	Allergy	1.84×10^{-2}	0.753	0.981
Monocyte count	Infection	7.54×10^{-3}	0.497	0.988

Eosinophil count	Influenza (ICD)	2.35×10^{-3}	1.779	0.999
Eosinophil count	*Legionella spp*. (ICD)	1.90×10^{-3}	5.564	1.000
Eosinophil count	*Neisseria meningitidis* (ICD)	1.34×10^{-3}	1.966	0.999
Eosinophil count	Papillomavirus (ICD)	2.81×10^{-3}	7.500	1.000
Eosinophil count	Cervical intraepithelial neoplasia (SR)	3.80×10^{-3}	0.465	0.993
Eosinophil count	Alzheimer's	2.12×10^{-3}	2.278	0.999
Eosinophil count	Hayfever	9.50×10^{-4}	0.456	0.998
Eosinophil count	Psoriasis	1.94×10^{-3}	10.017	1.000
Eosinophil count	Ulcerative colitis	1.45×10^{-3}	1.010	0.999
Eosinophil count	Allergy	2.90×10^{-2}	0.279	0.917
Eosinophil count	Infection	4.50×10^{-3}	5.320	0.999
Lymphocyte count	Influenza (ICD)	2.38×10^{-3}	2.949	0.999
Lymphocyte count	*Legionella spp*. (ICD)	1.94×10^{-3}	0.573	0.997
Lymphocyte count	*Neisseria meningitidis* (ICD)	1.50×10^{-3}	1.009	0.999
Lymphocyte count	Papillomavirus (ICD)	2.97×10^{-3}	1.230	0.998
Lymphocyte count	Cervical intraepithelial neoplasia (SR)	-3.99×10^{-4}	2.735	1.000
Lymphocyte count	Alzheimer's	1.76×10^{-3}	0.403	0.997
Lymphocyte count	Hayfever	2.18×10^{-3}	0.454	0.996
Lymphocyte count	Psoriasis	1.66×10^{-3}	0.784	0.998
Lymphocyte count	Ulcerative colitis	1.56×10^{-3}	0.707	0.998
Lymphocyte count	Allergy	1.96×10^{-2}	0.204	0.923
Lymphocyte count	Infection	-3.20×10^{-4}	0.476	0.999
Platelet count	Influenza (ICD)	2.08×10^{-3}	0.765	0.998
Platelet count	*Legionella spp*. (ICD)	1.75×10^{-3}	67.426	1.000
Platelet count	*Neisseria meningitidis* (ICD)	9.03×10^{-4}	0.545	0.999
Platelet count	Papillomavirus (ICD)	2.39×10^{-3}	1.139	0.998
Platelet count	Cervical intraepithelial neoplasia (SR)	6.24×10^{-4}	59.720	1.000
Platelet count	Alzheimer's	2.23×10^{-3}	1.108	0.998
Platelet count	Hayfever	2.10×10^{-3}	1.879	0.999
Platelet count	Psoriasis	1.28×10^{-3}	7.416	1.000
Platelet count	Ulcerative colitis	1.46×10^{-3}	0.668	0.998
Platelet count	Allergy	3.37×10^{-2}	13.029	0.998
Platelet count	Infection	2.13×10^{-3}	0.438	0.996

Table S17: Power calculations for Mendelian Randomisation analyses for causal effect of blood cell counts on counts on infection and inflammation-related disease

Exposure	Outcome	Power for Full cohort
RBC count	Influenza (ICD)	2.50%
RBC count	*Legionella spp*. (ICD)	2.50%
RBC count	*Neisseria meningitidis* (ICD)	2.50%
RBC count	Papillomavirus (ICD)	2.50%
RBC count	Cervical intraepithelial neoplasia (SR)	2.50%
RBC count	Alzheimer's	2.50%
RBC count	Hayfever	2.50%
RBC count	Psoriasis	2.50%
RBC count	Ulcerative colitis	2.50%
RBC count	Allergy	3.00%
RBC count	Infection	2.50%
Basophil count	Influenza (ICD)	2.50%
Basophil count	*Legionella spp*. (ICD)	2.50%
Basophil count	*Neisseria meningitidis* (ICD)	2.50%
Basophil count	Papillomavirus (ICD)	2.50%
Basophil count	Cervical intraepithelial neoplasia (SR)	2.50%
Basophil count	Alzheimer's	2.50%
Basophil count	Hayfever	2.50%
Basophil count	Psoriasis	2.50%
Basophil count	Ulcerative colitis	2.50%
Basophil count	Allergy	2.80%
Basophil count	Infection	2.60%
Neutrophil count	Influenza (ICD)	2.50%
Neutrophil count	*Legionella spp*. (ICD)	2.50%
Neutrophil count	*Neisseria meningitidis* (ICD)	2.50%
Neutrophil count	Papillomavirus (ICD)	2.50%
Neutrophil count	Cervical intraepithelial neoplasia (SR)	2.50%
Neutrophil count	Alzheimer's	2.50%
Neutrophil count	Hayfever	2.50%
Neutrophil count	Psoriasis	2.50%
Neutrophil count	Ulcerative colitis	2.50%
Neutrophil count	Allergy	2.70%
Neutrophil count	Infection	2.60%
Monocyte count	Influenza (ICD)	2.50%
Monocyte count	*Legionella spp*. (ICD)	2.50%
Monocyte count	*Neisseria meningitidis* (ICD)	2.50%
Monocyte count	Papillomavirus (ICD)	2.50%
Monocyte count	Cervical intraepithelial neoplasia (SR)	2.50%
Monocyte count	Alzheimer's	2.50%
Monocyte count	Hayfever	2.50%
Monocyte count	Psoriasis	2.50%
Monocyte count	Ulcerative colitis	2.50%
Monocyte count	Allergy	2.90%
Monocyte count	Infection	2.60%

Eosinophil count	Influenza (ICD)	2.50%
Eosinophil count	*Legionella spp*. (ICD)	2.50%
Eosinophil count	*Neisseria meningitidis* (ICD)	2.50%
Eosinophil count	Papillomavirus (ICD)	2.50%
Eosinophil count	Cervical intraepithelial neoplasia (SR)	2.50%
Eosinophil count	Alzheimer's	2.50%
Eosinophil count	Hayfever	2.50%
Eosinophil count	Psoriasis	2.50%
Eosinophil count	Ulcerative colitis	2.50%
Eosinophil count	Allergy	2.70%
Eosinophil count	Infection	2.50%
Lymphocyte count	Influenza (ICD)	2.50%
Lymphocyte count	*Legionella spp*. (ICD)	2.50%
Lymphocyte count	*Neisseria meningitidis* (ICD)	2.50%
Lymphocyte count	Papillomavirus (ICD)	2.50%
Lymphocyte count	Cervical intraepithelial neoplasia (SR)	2.50%
Lymphocyte count	Alzheimer's	2.50%
Lymphocyte count	Hayfever	2.50%
Lymphocyte count	Psoriasis	2.50%
Lymphocyte count	Ulcerative colitis	2.50%
Lymphocyte count	Allergy	3.00%
Lymphocyte count	Infection	2.50%
Platelet count	Influenza (ICD)	2.50%
Platelet count	*Legionella spp*. (ICD)	2.50%
Platelet count	*Neisseria meningitidis* (ICD)	2.50%
Platelet count	Papillomavirus (ICD)	2.50%
Platelet count	Cervical intraepithelial neoplasia (SR)	2.50%
Platelet count	Alzheimer's	2.50%
Platelet count	Hayfever	2.50%
Platelet count	Psoriasis	2.50%
Platelet count	Ulcerative colitis	2.50%
Platelet count	Allergy	2.80%
Platelet count	Infection	2.50%

Table S18: Variance explained and F statistics for rs28362491 for blood cell counts from the full UK Biobank cohort.

Phenotype	Variance	F statistic
RBC count	8.20×10^{-5}	38.739
Basophil count	7.64×10^{-5}	36.011
Neutrophil count	2.84×10^{-5}	13.399
Monocyte count	1.32×10^{-4}	62.484
Eosinophil count	2.32×10^{-5}	10.960
Lymphocyte count	2.18×10^{-4}	103.028
Leukocyte count	4.94×10^{-9}	0.002
Platelet count	2.20×10^{-5}	10.414

References

1. Brenner, N., Mentzer, A.J., Butt, J., Michel, A., Prager, K., Brozy, J., Weißbrich, B., Aiello, A.E., Meier, H.C.S., Breuer, J., et al. (2018). Validation of Multiplex Serology detecting human herpesviruses 1-5. PLoS One 13, e0209379-e0209379.
2. Brenner, N., Mentzer, A.J., Butt, J., Braband, K.L., Michel, A., Jeffery, K., Klenerman, P., Gärtner, B., Schnitzler, P., Hill, A., et al. (2019). Validation of Multiplex Serology for human hepatitis viruses B and C, human T-lymphotropic virus 1 and Toxoplasma gondii. PLoS One 14, e0210407-e0210407.
3. Dondog, B., Schnitzler, P., Michael, K.M., Clifford, G., Franceschi, S., Pawlita, M., and Waterboer, T. (2015). Hepatitis C Virus Seroprevalence in Mongolian Women Assessed by a Novel Multiplex Antibody Detection Assay. Cancer Epidemiology Biomarkers & Prevention 24, 1360-1365.
4. Kranz, L.M., Gärtner, B., Michel, A., Pawlita, M., Waterboer, T., and Brenner, N. (2019). Development and validation of HIV-1 Multiplex Serology. Journal of Immunological Methods 466, 47-51.
5. Gossai, A., Waterboer, T., Nelson, H.H., Doherty, J.A., Michel, A., Willhauck-Fleckenstein, M., Farzan, S.F., Christensen, B.C., Hoen, A.G., Perry, A.E., et al. (2016). Prospective Study of Human Polyomaviruses and Risk of Cutaneous Squamous Cell Carcinoma in the United States. Cancer Epidemiology Biomarkers & Prevention 25, 736-744.
6. Kjærheim, K., Røe, O.D., Waterboer, T., Sehr, P., Rizk, R., Dai, H.Y., Sandeck, H., Larsson, E., Andersen, A., Boffetta, P., et al. (2007). Absence of SV40 antibodies or DNA fragments in prediagnostic mesothelioma serum samples. International Journal of Cancer 120, 2459-2465.
7. Robles, C., Casabonne, D., Benavente, Y., Costas, L., Gonzalez-Barca, E., Aymerich, M., Campo, E., Tardon, A., Jiménez-Moleón, J.J., Castaño-Vinyals, G., et al. (2015). Seroreactivity against Merkel cell polyomavirus and other polyomaviruses in chronic lymphocytic leukaemia, the MCC-Spain study. Journal of General Virology 96, 2286-2292.
8. Sehr, P., Müller, M., Höpfl, R., Widschwendter, A., and Pawlita, M. (2002). HPV antibody detection by ELISA with capsid protein L1 fused to glutathione S-transferase. Journal of Virological Methods 106, 61-70.
9. Sehr, P., Zumbach, K., and Pawlita, M. (2001). A generic capture ELISA for recombinant proteins fused to glutathione S-transferase: validation for HPV serology. Journal of Immunological Methods 253, 153-162.
10. Hulstein, S.H., Matser, A., Alberts, C.J., Snijder, M.B., Willhauck-Fleckenstein, M., Hufnagel, K., Prins, M., de Vries, H.J.C., Schim van der Loeff, M.F., and Waterboer, T. (2018). Differences in Chlamydia trachomatis seroprevalence between ethnic groups cannot be fully explained by socioeconomic status, sexual healthcare seeking behavior or sexual risk behavior: a cross-sectional analysis in the HEalthy LIfe in an Urban Setting (HELIUS) study. BMC Infectious Diseases 18, 612-612.
11. Trabert, B., Waterboer, T., Idahl, A., Brenner, N., Brinton, L.A., Butt, J., Coburn, S.B., Hartge, P., Hufnagel, K., Inturrisi, F., et al. (2018). Antibodies Against Chlamydia trachomatis and Ovarian Cancer Risk in Two Independent Populations. JNCI: Journal of the National Cancer Institute 111, 129-136.
12. Michel, A., Waterboer, T., Kist, M., and Pawlita, M. (2009). Helicobacter pylori Multiplex Serology. Helicobacter 14, 525-535.
13. Waage, J., Standl, M., Curtin, J.A., Jessen, L.E., Thorsen, J., Tian, C., Schoettler, N., andMe Research, T., collaborators, A., Flores, C., et al. (2018). Genome-wide association and HLA fine-mapping studies identify risk loci and genetic pathways underlying allergic rhinitis. Nat Genet 50, 1072-1080.
14. Johansson, A., Rask-Andersen, M., Karlsson, T., and Ek, W.E. (2019). Genome-wide association analysis of 350 000 Caucasians from the UK Biobank identifies novel loci for asthma, hay fever and eczema. Hum Mol Genet 28, 4022-4041.
15. Tian, C., Hromatka, B.S., Kiefer, A.K., Eriksson, N., Noble, S.M., Tung, J.Y., and Hinds, D.A. (2017). Genome-wide association and HLA region fine-mapping studies identify susceptibility loci for multiple common infections. Nat Commun 8, 599-599.
16. Bajwa, E.K., Cremer, P.C., Gong, M.N., Zhai, R., Su, L., Thompson, B.T., and Christiani, D.C. (2011). An NFKB1 promoter insertion/deletion polymorphism influences risk and outcome in acute respiratory distress syndrome among Caucasians. PLoS One 6, e19469-e19469.

17. Ellinghaus, D., Jostins, L., Spain, S.L., Cortes, A., Bethune, J., Han, B., Park, Y.R., Raychaudhuri, S., Pouget, J.G., Hubenthal, M., et al. (2016). Analysis of five chronic inflammatory diseases identifies 27 new associations and highlights disease-specific patterns at shared loci. Nat Genet 48, 510-518.
18. Yenmis, G., Oner, T., Cam, C., Koc, A., Kucuk, O.S., Yakicier, M.C., Dizman, D., and Kanigur Sultuybek, G. (2015). Association of NFKB1 and NFKBIA polymorphisms in relation to susceptibility of Behcet's disease. Scand J Immunol 81, 81-86.
19. Seidi, A., Mirzaahmadi, S., Mahmoodi, K., and Soleiman-Soltanpour, M. (2018). The association between NFKB1 -94ATTG ins/del and NFKB1A 826C/T genetic variations and coronary artery disease risk. Mol Biol Res Commun 7, 17-24.
20. Mishra, A., Srivastava, A., Mittal, T., Garg, N., and Mittal, B. (2013). Role of inflammatory gene polymorphisms in left ventricular dysfunction (LVD) susceptibility in coronary artery disease (CAD) patients. Cytokine 61, 856-861.
21. Lai, H.-M., Li, X.-M., Yang, Y.-N., Ma, Y.-T., Xu, R., Pan, S., Zhai, H., Liu, F., Chen, B.-D., and Zhao, Q. (2015). Genetic Variation in NFKB1 and NFKBIA and Susceptibility to Coronary Artery Disease in a Chinese Uygur Population. PLOS ONE 10, e0129144-e0129144.
22. Adamzik, M., Frey, U.H., Rieman, K., Sixt, S., Beiderlinden, M., Siffert, W., and Peters, J. (2007). Insertion/deletion polymorphism in the promoter of NFKB1 influences severity but not mortality of acute respiratory distress syndrome. Intensive Care Med 33, 1199-1203.
23. Dudding, T., Haworth, S., Lind, P.A., Sathirapongsasuti, J.F., andMe Research, T., Tung, J.Y., Mitchell, R., Colodro-Conde, L., Medland, S.E., Gordon, S., et al. (2019). Genome wide analysis for mouth ulcers identifies associations at immune regulatory loci. Nat Commun 10, 1052-1052.
24. Boccardi, V., Rizzo, M.R., Marfella, R., Papa, M., Esposito, A., Portoghese, M., Paolisso, G., and Barbieri, M. (2011). -94 ins/del ATTG NFKB1 gene variant is associated with lower susceptibility to myocardial infarction. Nutr Metab Cardiovasc Dis 21, 679-684.
25. Kawashima, M., Hitomi, Y., Aiba, Y., Nishida, N., Kojima, K., Kawai, Y., Nakamura, H., Tanaka, A., Zeniya, M., Hashimoto, E., et al. (2017). Genome-wide association studies identify PRKCB as a novel genetic susceptibility locus for primary biliary cholangitis in the Japanese population. Hum Mol Genet 26, 650-659.
26. Gonzalez-Serna, D., Lopez-Isac, E., Yilmaz, N., Gharibdoost, F., Jamshidi, A., Kavosi, H., Poursani, S., Farsad, F., Direskeneli, H., Saruhan-Direskeneli, G., et al. (2019). Analysis of the genetic component of systemic sclerosis in Iranian and Turkish populations through a genome-wide association study. Rheumatology (Oxford) 58, 289-298.
27. Liou, Y.J., Wang, H.H., Lee, M.T., Wang, S.C., Chiang, H.L., Chen, C.C., Lin, C.H., Chung, M.S., Kuo, C.C., Liao, D.L., et al. (2012). Genome-wide association study of treatment refractory schizophrenia in Han Chinese. PLoS One 7, e33598-e33598.
28. Jostins, L., Ripke, S., Weersma, R.K., Duerr, R.H., McGovern, D.P., Hui, K.Y., Lee, J.C., Schumm, L.P., Sharma, Y., Anderson, C.A., et al. (2012). Host-microbe interactions have shaped the genetic architecture of inflammatory bowel disease. Nature 491, 119-124.
29. Karban, A.S., Okazaki, T., Panhuysen, C.I., Gallegos, T., Potter, J.J., Bailey-Wilson, J.E., Silverberg, M.S., Duerr, R.H., Cho, J.H., Gregersen, P.K., et al. (2004). Functional annotation of a novel NFKB1 promoter polymorphism that increases risk for ulcerative colitis. Hum Mol Genet 13, 35-45.
30. Borm, M.E., van Bodegraven, A.A., Mulder, C.J., Kraal, G., and Bouma, G. (2005). A NFKB1 promoter polymorphism is involved in susceptibility to ulcerative colitis. Int J Immunogenet 32, 401-405.
31. Wang, D., Xie, T., Xu, J., Wang, H., Zeng, W., Rao, S., Zhou, K., Pei, F., and Zhou, Z. (2016). Genetic association between NFKB1 -94 ins/del ATTG Promoter Polymorphism and cancer risk: a meta-analysis of 42 case-control studies. Sci Rep 6, 30220-30220.
32. Kanai, M., Akiyama, M., Takahashi, A., Matoba, N., Momozawa, Y., Ikeda, M., Iwata, N., Ikegawa, S., Hirata, M., Matsuda, K., et al. (2018). Genetic analysis of quantitative traits in the Japanese population links cell types to complex human diseases. Nat Genet 50, 390-400.
33. Loh, P.R., Tucker, G., Bulik-Sullivan, B.K., Vilhjalmsson, B.J., Finucane, H.K., Salem, R.M., Chasman, D.I., Ridker, P.M., Neale, B.M., Berger, B., et al. (2015). Efficient Bayesian mixed-model analysis increases association power in large cohorts. Nat Genet 47, 284-290.

34. Jonsson, S., Sveinbjornsson, G., de Lapuente Portilla, A.L., Swaminathan, B., Plomp, R., Dekkers, G., Ajore, R., Ali, M., Bentlage, A.E.H., Elmer, E., et al. (2017). Identification of sequence variants influencing immunoglobulin levels. Nat Genet 49, 1182-1191.
